# Impact of HIV infection on cervical intraepithelial neoplasia detection in pregnant and non-pregnant women in Germany: a cross-sectional study

**DOI:** 10.1007/s00404-024-07813-7

**Published:** 2024-11-07

**Authors:** Irena Rohr, Anna Sophie Skof, Michaela Heinrich-Rohr, Fabian Weiss, Jan-Peter Siedentopf, Katharina von Weizsäcker, Irene Alba Alejandre, Wolfgang Henrich, Jalid Sehouli, Charlotte K. Metz

**Affiliations:** 1 Department of Obstetrics, Charité-Universitätsmedizin Berlin, Corporate Member of Freie Universität Berlin and Humboldt-Universität zu Berlin, Augustenburger Platz 1, 13353 Berlin, Germany; 2 HPV Research Laboratory, Department of Gynecology, Charité-Universitätsmedizin Berlin, Corporate Member of Freie Universität Berlin and Humboldt-Universität zu Berlin, Augustenburgerplatz 1, 13353 Berlin, Germany; 3 Duale Hochschule Schleswig-Holstein (DHSH), Hans-Detlev-Prien-Str. 10, 24106 Kiel, Germany; 4 Department of Obstetrics and Gynecology, University Hospital, Ludwig-Maximilians-University (LMU), Marchioninistrasse 15, 81377 Munich, Germany

**Keywords:** HIV, HPV, Pregnancy, Dysplasia

## Abstract

**Purpose:**

Women living with HIV (WLWH) are frequently affected by cervical dysplasia caused by Human Papillomavirus (HPV) and invasive cervical cancer (CxCa). CxCa screening programs can include colposcopy, cytology, and HPV testing. These methods, however, have limitations in effectively stratifying cervical dysplasia. This study aimed to evaluate the applicability of an innovative mRNA-based multiplexed expression-quantifying assay in the detection and assessment of cervical dysplasia in WLWH.

**Methods:**

The QuantiGene-Molecular-Profiling-Histology Assay (QG-MPH) was used to detect and quantify HPV oncogene and cellular biomarker mRNA expression. These results were included in the Risk Score (QG-MPH RS) calculations that inform about the presence and severity of dysplasia. QG-MPH RS results were compared to the highly sensitive Multiplexed Papillomavirus Genotyping (MPG) Assay and clinical results obtained by cytology, colposcopy and histology. For a standardized nomenclature of clinical results, the clinical ASSIST Score was used.

**Results:**

Of 241 WLWH, including 96 pregnant women, a concordance between the QG-MPH RS and the ASSIST Score was found to 36.3% (49/135) in non-pregnant WLWH and 67.1% (57/85) in pregnant WLWH. The QG-MPH method demonstrated high specificity for detecting high-risk HPV (HR-HPV) genotypes and high-grade cervical dysplasia, achieving 89.6% and 82.4%, respectively, including pregnant and non-pregnant WLWH.

**Conclusion:**

The QG-MPH assay shows potential for improving the detection and management of HPV-related cervical dysplasia in WLWH, including pregnant women. Its high specificity, however, is tempered by its tendency to overestimate dysplasia severity in certain cases, indicating that further research is needed to refine its use as a reliable diagnostic tool for this high-risk population.

**Supplementary Information:**

The online version contains supplementary material available at 10.1007/s00404-024-07813-7.

## What does this study add to the clinical work

**Table:** 

The mRNA-based Quantigene test promises high specificity in detecting high-grade intraepithelial dysplasia und HR-HPV infection in pregnant and non-pregnant women living with HIV in Germany. However, its use in this special cohort seems to be influenced by HIV status and pregnancy. Therefore, larger and more comprehensive studies are crucial to enhance its diagnostic accuracy.	

## Introduction

Increasingly, studies have highlighted the elevated risk of HPV infection and its association with the incidence of cervical intraepithelial neoplasia (CIN) and progression to cervical cancer (CxCa) in women living with the Human Immunodeficiency Virus (HIV) (WLWH), including those who are pregnant [1, 2]. CxCa is the fourth most common cancer worldwide and also an Acquired Immune Deficiency Syndrome (AIDS) defining illness [3]. WLWH face a sixfold increased risk of developing CxCa during their lifetime compared to HIV-negative women [4]. Additionally, pregnancy also appears to be a risk factor for persistent HPV infection and dysplasia due to immune downregulation [5, 6].

Globally, HPV genotypes are widespread and can be broadly categorized into high-risk HPV genotypes (HR-HPV) with oncogenic potential and low-risk HPV genotypes (LR-HPV) [7–9]. However, most HPV infections are spontaneously resolved within 12–24 months [10, 11]. In about 10% of cases, HPV infections can persist and lead to CIN and, after years, to invasive CxCa. According to the World Health Organization (WHO) guidelines for screening and treatment of cervical precancer lesions for cervical cancer prevention, primary prevention therefore includes HPV vaccination for girls and boys aged between 9 and 14 years [12]. From the age of 25 years, women should be screened using triage strategies that include HPV DNA testing, cytology, and visual inspection with acetic acid (VIA). WHO guidelines further advise that WLWH should be screened more frequently than HIV-negative women for the prevention of CxCa: at least every 3–5 years. In the case of HR-HPV positivity or conspicuous cytology, more frequent check-ups and colposcopy with biopsy for histological classification are recommended [13, 14]. Current screening methods, such as cytology and colposcopy, have low sensitivity and are subject to influence by the expertise and knowledge of the clinician and analyzing doctor [15–17]. Histological classification, as obtained by colposcopically guided biopsies, is a more invasive form of diagnosis, as are subsequent therapies such as conization for high-grade dysplasia. It should also be recognized that current screening methods for the prevention of cervical cancer do not distinguish between transient, persistent intraepithelial neoplasia, and spontaneous regression. This lack of certainty in the diagnosis may lead to prolonged terms of follow-up and overtreatment [16]. In a previous trial, Skof et al*.* showed that the mRNA-based QuantiGene™ 2.0 Plex Assay (QG) had sufficient sensitivity to detect cervical dysplasia and discriminate low-risk and high-risk dysplasia in HIV-negative patients [18]. Assuming that the HPV oncogenic regions E7/E6 are upregulated in progression, but also in high-grade cervical intraepithelial neoplasia (CIN III) and CxCa, this could contribute to the detection of surveillance-requiring dysplasia and therapeutically relevant dysplasia with a less-invasive method.

The aim of this study is to evaluate the applicability of the QuantiGene Molecular Profiling and Histology Assay (QG-MPH) in the specific population of pregnant and non-pregnant WLWH for the detection of HPV infections and cervical dysplasia. The QG-MPH analyzes 19 HPV genotypes, including one LR-HPV genotype and 18 HR- or potentially HR-HPV genotypes in addition to several cellular markers, proliferation markers, stem cell markers, oncogenes, and tumor suppressor genes.

## Methods

### Study patient, sampling, and clinical evaluation

Patients were studied at the outpatient Clinic for Infectious Diseases at the Department of Obstetrics, Charité-Universitätsmedizin Berlin from October 2017 to August 2021, and at the LMU Munich from February 2021 to August 2021. Following written informed consent, pregnant and non-pregnant WLWH were included in the study. These patients underwent routine gynecological monitoring at both centers for their HIV infection and pregnancy counseling if applicable. Additionally, these women received care from external infectious disease specialists. Ethical approval was obtained from the ethical committees in Berlin (IRB number EA4/098/19) and Munich (IRB number 20-1040). Cervical cancer screening, including cervical cytology, HPV genotyping, colposcopy, and histology, was performed according to the German guidelines for the prevention of cervical cancer [14]. For all patients, a smear sample was collected and then immediately fixed in 20 mL of PreserveCyt^®^ solution (Thinprep, Hologic, Marlborough, MA, USA). Smear samples were taken for non-pregnant women with the MedGyn Pap cell^®^ brush (Illinois, USA) and for pregnant women with the more delicate PapCone^®^ brush (Hannover, Germany). From these samples, 2 mL each was used for HPV genotyping and the QuantiGeneTM 2.0 Plex Assay. Cytology and histological results were classified using the Münchner Nomenklatur III and transferred into Bethesda Nomenclature [19]. For colposcopy results, the “Rio 2011 Colposcopy Nomenclature” was used [20]. The ASSIST Score was used to standardize the results, as not all patients received a histology testing [21]. The ASSIST Score classifies the outcome as either normal, low-grade cervical intraepithelial neoplasia (LCIN) or high-grade cervical intraepithelial neoplasia (HCIN), when taking the cytological, colposcopic, and histological results into account. LCIN corresponds to CIN I, and HCIN includes CIN II, CIN III, and cervical carcinoma (CxCa) [21]. In accordance with the German–Austrian guidelines for HIV management during pregnancy and the German–Austrian guidelines for Antiretroviral Therapy of HIV-1 Infection, participants in the study were administered antiretroviral therapy (ART), which includes a variety of drug classes suitable for pregnancy [22, 23]. These guidelines recommend combination ART for both pregnant and non-pregnant women, typically consisting of two nucleoside/nucleotide reverse transcriptase inhibitors (NRTIs) combined with an integrase inhibitor (INI), a non-nucleoside/nucleotide reverse transcriptase inhibitor (NNRTI), or a protease inhibitor (PI). However, regulatory approval for most antiretroviral medication during pregnancy was limited, often due to a lack of comprehensive clinical evidence. Consequently, modifications to the treatment regimen may be necessary in cases of HIV resistance, drug intolerance, or issues with treatment adherence [22, 23].

### Multiplexed genotyping assay

HPV positivity was determined using the sensitive Multiplexed Papillomavirus Genotyping Assay (MPG), discriminating 18 HR-HPV genotypes (HPV 16, 18, 26, 31, 33, 35, 39, 45, 51, 52, 53, 56, 58, 59, 66, 68, 73, 82) and 9 LR-HPV genotypes (HPV 6, 11, 42, 43, 54, 57, 70, 72, 90). For the PCR a broad-spectrum GP5+/GP6 +-bio primer set was used, targeting the L1 gene [24, 25]. Following the PCR, the products were hybridized to Luminex suspension beads conjugated with genotype-specific probes (Luminex, Austin, TX, USA) and stained with phycoerythrin-conjugated streptavidin (SAPE). The BioPlex 200 reader (BioRad, Hercules, CA, USA) was used for read-out.

### QuantiGene molecular profiling histology assay (QG-MPH)

For mRNA quantification using the QuantiGene™ 2.0 Plex Assay (Thermo Fisher Scientific, Waltham, MA, USA), a custom plex-set called QuantiGene molecular profiling histology (QG-MPH) was designed, that targets the gene transcripts of HR-HPV genotypes, reference markers and cellular biomarkers.

A crude cell lysate of the cervical smear samples was produced using the QuantiGene™ sampling processing Kit for blood samples (Thermo Fisher, Waltham, MA, USA). 2 mL of the Thinprep containing patients’ cells was centrifuged, the supernatant was discarded, and the pellet was lysed in 100 µL of Working Lysis Mix containing 17 µg Proteinase K at 50 °C and 850 rpm for 30–40 min. Of this lysate, 20 µL was used as sample input for QG-MPH and mixed with 5 µL of the working bead mix including the probe set, capture beads, blocking reagent, and Proteinase K, according to the assay protocol. During the incubation at 54 °C and 600 rpm for 18–22 h, the target transcripts hybridized to specific DNA capture probes that were conjugated to color-coded Luminex beads. A signal amplification is followed by a series of hybridization reactions of the Pre-Amplifier, Amplifier, and Label probe. The biotinylated Label Probe was stained using SAPE. The fluorescence signals of the individual Luminex beads and specific target transcripts were detected using a BioPlex 200 Luminex reader (BioRad) and reported as Mean Fluorescence Intensity (MFI) proportional to the number of RNA molecules bound to the bead. Each measurement included three negative controls (NC). HPV positivity was defined by an MFI value above the limit of detection (LOD), defined as the mean of the three NC plus three times their standard deviation (SD). For the quantification analysis, the MFI of each individual marker was corrected for background by subtraction of the mean NC signal. The corrected MFI values were normalized to the MFI signals of Beta-Actin (ACTB) expression. Validation showed that an ACTB value of at least 100 MFI was necessary for a reliable normalization. The measurement of samples with a lower ACTB MFI value was repeated. The normalized results are presented as relative Median Fluorescence Intensity (rMFI). For Risk Score (RS) evaluation in multiple infections, the “leading HPV” was defined as the HPV genotype with the highest measured MFI value. RS was developed in a prospective trial by logistic regression and Receiver Operating Characteristic curve (ROC) analysis (International publication number: WO2020/161285 A11) [26]. rMFI values were used for the calculation of the RS results. If the rMFI value for the individual biomarker is over the respective cutoff, the marker enters the formula with a 1 and the constant is considered. Values above the cutoffs for the different borders CIN2+, CIN3+ and CxCa of 0.51, 0.19, and 0.02, respectively, were considered positive for the individual RS (26). Individual RS results were combined into one result for each patient: < CIN2 if all RSs were negative, CIN2 if only CIN2+ RS was positive, CIN3 if CIN2+ and CIN3+ RS were positive, and CxCa if all three RSs were positive. The result was rated as inconsistent if another combination of RS positivity was seen. Samples were rated as invalid if the sample showed < 100 MFI for ACTB, even after repetition. RS results were compared to the ASSIST Score results of the patients [21].

### ASSIST score

The ASSIST Score was employed to estimate the stage of dysplasia based on screening results, even when histological findings were not available. This scoring system integrates colposcopic and cytological outcomes from the patients. When histological data were available, they were directly incorporated into the ASSIST Score to provide a more comprehensive assessment and grading of dysplasia [21].

### Statistical analysis

Statistical analysis was performed using IBM^®^ SPSS^®^ Statistics release 27.0 (SPSS Inc. an IBM Company, Chicago, Illinois, USA, 2020). Descriptive data are presented using percentage frequencies. The mean with standard deviation (Range) or median with interquartile range (IQR) was calculated for continuous variables. HR-HPV genotypes were divided into three groups according to their oncogenic potential: (1) HR-HPV genotypes 16,18; (2) HR-HPV genotypes 31, 33, 35, 39, 45, 51, 52, 56, 58, 59 and (3) potentially HR-HPV genotypes 66, 68a, 68b, 26, 53, 73, 82. Detection of HR-HPV genotypes by QG-MPH and MPG among pregnant and non-pregnant WLWH was presented in relation to the QG-MPH RS results through the use of bar charts. The QG-MPH RS results were compared with the ASSIST Score results by dividing them into three groups: Missed, correct, overrated. Results are presented in percentage frequencies using bar charts [21].

## Results

### Demographic and clinical characteristics of the study population

A total of 241 WLWH were included in this study, of which 96 were pregnant. The average age was 32 years for the pregnant WLWH and 43 years for the non-pregnant WLWH. In the group of pregnant WLWH, the median duration since HIV diagnosis was 4.0 years, while among non-pregnant WLWH, it was 12.0 years. At the time of study inclusion, 95.7% (90/94) of pregnant WLWH and 95.8% (138/144) of non-pregnant WLWH were on ART. By study inclusion among pregnant WLWH, 72.0% (67/93) had a HIV viral load < 50 copies/mL, compared to 88.8% (127/143) among non-pregnant WLWH. CD4 cell counts were ≥ 350 cells/μL in 76.1% (70/92) of pregnant WLWH and in 84.7% (111/131) of non-pregnant WLWH. 45.8% (44/96) of pregnant WLWH were in the third trimester of pregnancy, 33.3% (32/96) were in the second trimester of pregnancy, and 20.8% (20/96) were in the first trimester of pregnancy (Table 1).

**Table 1:** Demographic and clinical data of the study cohort of pregnant and non-pregnant WLWH

Variables	Pregnant WLWH	Non-pregnant WLWH
Age median	32.0 IQR (18–44)	43.0 IQR (19–66)
< 25 years	16/96 (16.7)	1/145 (0.7)
≥ 25 years	80/96 (83.3)	144/145 (99.3)
Time since HIV diagnosis in years: median (IQR)	4.0 (< 1–32)	12.0 (< 1–34)
HIV viral load: mean (range)	6061.13 (0–211,000)	11,801.53 Range 0–1,350,000
< 50 copies/mL	67/93 (72.0)	127/143 (88.8)
≥ 50 copies/mL	26/93 (28.0.1)	16/143 (11.2)
CD4 cells/μL median (IQR)	504.00 (47–1318)	580.00 (20–1969)
< 350 cells/μL	22/92 (23.9)	20/131 (15.3)
≥ 350 cells/μL	70/92 (76.1)	111/131 (84.7)
ART	90/94 (95.7)	138/144 (95.8)
ART according to guidelines	84/94 (89.4)	114/144 (79.2)
Gravidity mean	3.0 Range 1–9	–
1. Trimester	20/96 (20.8)	–
2. Trimester	32/96 (33.3)	–
3. Trimester	44/96 (45.8)	–
Cytology	87/96 (90.6)	140/145 (96.6)
Normal (NILM)	55/87 (63.2)	113/140 (80.7)
Abnormal (≥ ASCUS)	32/87 (36.8)	27/140 (19.3)
ASCUS	18/32 (56.3)	13/27 (48.1)
LSIL	9/32 (28.1)	6/27 (22.2)
HSIL	5/32 (15.6)	8/27 (29.6)
Histology	8/96 (8.3)	17/145 (11.7)
Not evaluable	–	3/17 (17.6)
Normal	3/8 (37.5)	2/17 (11.8)
Abnormal	5/8 (62.5)	12/17 (70.6)
Cervicitis	3/5 (60.0)	–
CIN I	–	7/12 (58.3)
CIN II	–	2/12 (16.7)
CIN III	2/5 (40.0)	3/12 (25.0)
Colposcopy done	93/96 (96.9)	145/145 (100)
not assessable	19/93 (20.4)	68/145 (46.9)
Normal	46/93 (49.5)	56/145 (38.6)
Abnormal	28/93 (30.1)	21/145 (14.5)
Minor change	17/28 (60.7)	16/21 (76.2)
Major change	11/28 (39.3)	5/21 (9.5)
MPG HPV positive	49/95 (51.6)	60/142 (42.3)
HR-HPV positive in MPG	43/95 (45.3)	55/142 (38.7)
MPG HR-HPV 16,18	27/95 (28.4)	24/143 (16.8)
MPG HR-HPV others (31, 33, 35, 39, 45, 51, 52, 56, 58, 59)	31/95 (32.6)	37/143 (25.9)
MPG pHR-HPV (66, 68a, 68b, 26, 53, 73, 82)	12/95 (12.6)	15/143 (10.5)
MPG multiple infection	26/95 (27.4)	28/142 (19.7)
QG HPV positive	26/95 (27.4)	24/141 (17)
QG HR-HPV positive	25/95 (26.3)	23/141 (16.2)
QG HR-HPV 16,18	7/95 (7.4)	4/141 (2.8)
QG HR-HPV others (31, 33, 35, 39, 45, 51, 52, 56, 58, 59)	17/95 (17.9)	20/141 (14.2)
QG pHR HPV (66, 68a, 68b, 26, 53, 73, 82)	5/95 (5.3)	1/141 (0.7)
QG multiple infection	4/95 (4.2)	2/141 (1.4)
Calculated ASSIST score [21]	90/96 (93.8)	140/145 (96.6)
Normal	56/90 (62.2)	110/140 (78.6)
LCIN	26/90 (28.9)	22/140 (15.7)
HCIN	5/90 (5.6)	8/140 (5.7)

*WLWH* women living with HIV, *IQR* interquartile range, *HIV* human immunodeficiency virus, *ART* antiretroviral therapy, *NILM* negative for intraepithelial lesion or malignancy, *ASCUS* atypical squamous cells of undetermined significance, *LSIL* low-grade squamous intraepithelial lesion, *HSIL* high-grade squamous intraepithelial lesion, *CIN* cervical intraepithelial neoplasia, *MPG* multiplexed genotyping assay, *HPV* human papillomavirus, *HR-HPV* high-risk human papillomavirus, *pHR-HPV* potentially high-risk human papillomavirus, *QG* QuantiGene, *ASSIST* Association Studies assisted by Inference and Semantic Technologies [21], *LCIN* low grade cervical intraepithelial neoplasia, *HCIN* high grade cervical intraepithelial neoplasia

Cytology was conducted in 90.6% (87/96) of pregnant WLWH and 96.6% (140/145) of non-pregnant WLWH. The absence of cytology results in some cases was due to patient non-participation in screening or insufficient cell material for evaluation. Normal cytological findings were observed in 63.2% (55/87) of pregnant WLWH and 80.7% (113/140) of non-pregnant WLWH (Table 1).

Abnormal cytology, defined as equal or more severe than Atypical Squamous Cells of Undetermined Significance (≥ ASCUS), was found in 36.8% (32/87) of pregnant WLWH and in 19.3% (27/140) of non-pregnant WLWH. Concerning pregnant WLWH, 56.3% (18/32) had ASCUS, 28.1% (9/32) had low-grade squamous intraepithelial lesion (LSIL) and 15.6% (5/32) had high-grade squamous intraepithelial lesion (HSIL). Among the non-pregnant WLWH, 48.1% (13/27) had ASCUS, 22.2% (6/27) had LSIL and in 29.6% (8/27) HSIL was detected (Table 1).

In 28 out of 93 women (30.1%), abnormal colposcopy was detected. Among those with abnormal colposcopy findings, a colposcopic “Major Change” was observed in 39.3% (11/28) of pregnant WLWH, while 60.7% (17/28) showed a “Minor Change”.

Histological results were obtained from 8 pregnant WLWH with “Major Change”. Due to one rejection of biopsy and two inadequately representative biopsy materials, three histologic results are missing.

Histological results of non-pregnant WLWH were available from 11.7% (17/145) of participants. Abnormal histological results were found in 62.5% (5/8) of the biopsies from pregnant WLWH and in 70.6% (12/17) of the biopsies from non-pregnant WLWH.

In the group of the pregnant WLWH, 40% (2/5) had a diagnosis of CIN III and no CIN I or CIN II was found. 60% (3/5) pregnant WLWH had histologically detected cervicitis.

Regarding the abnormal histological results in biopsies of non-pregnant WLWH, 58.3% (7/12) had CIN I, 16.7% (2/12) had CIN II, and 25% (3/12) had CIN III (Table 1). CxCa was not detected in any of the participants (Table 1).

The categorization of the patients by the ASSIST Score resulted in an estimation of 62.2% (56/90) of pregnant WLWH having no dysplasia, 28.9% (26/90) with LCIN, and 5.6% (5/90) with HCIN (Table 1). The non-pregnant WLWH were categorized into 78.6% (110/140) with normal findings, 15.7% (22/140) with LCIN and 5.7% (8/140) with HCIN.

### Detection of HR-HPV genotypes in QG-MPH and MPG

The detection of HPV genotypes was conducted using both the MPG detection method and the QG-MPH Assay. The MPG method detected DNA of HPV genotypes in 51.6% (49/95) of pregnant WLWH, of which 45.3% (43/95) were HR-HPV genotypes. Among the non-pregnant WLWH, HPV genotypes were detected in 42.3% (60/142), where 38.7% (55/142) of these were HR-HPV genotypes. Specifically, the QG-MPH assay identified HR-HPV genotypes in 26.3% (25/95) of pregnant WLWH and in 16.2% (23/141) of non-pregnant WLWH.

Concerning the sensitivity and specificity of HR-HPV genotype detection, the QG-MPH exhibited a sensitivity of 35.0%, and a high specificity of 89.6% among the pregnant and non-pregnant WLWH included in this study.

### QuantiGene RS results

**Fig. 1:**
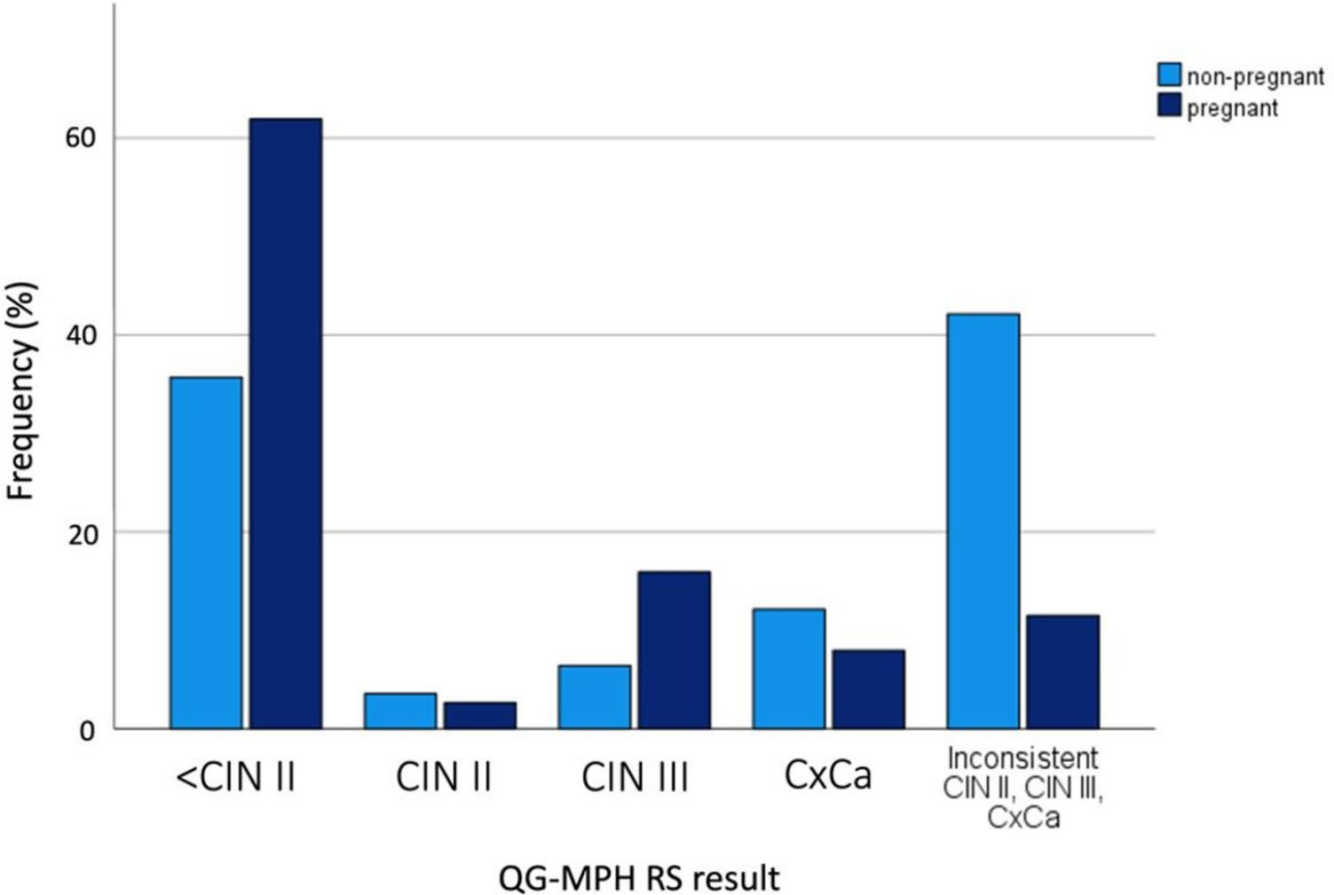
QG-MPH risk score (RS) results of pregnant and non-pregnant WLWH. The frequency in percentage is shown for the possible QG-MPH RS Results of 140 non-pregnant WLWH (light blue) and 94 pregnant WLWH (dark blue). Designed with IBM^®^ SPSS^®^ Statistics release 27.0 (SPSS Inc. an IBM Company, Chicago, Illinois, USA, 2020)

The QG-MPH RS detected a high rate of normal cervical findings up to slight cellular transformations below CIN II (< CIN II) in 64.7% (61/94) of pregnant WLWH and in 35.7% (50/140) of non-pregnant WLWH (Fig. 1). 2.1% (2/94) of pregnant WLWH and 3.6% (5/49) of non-pregnant WLWH presented a CIN II result. The QG-MPH RS result CIN III was seen in 12.8% (12/94) of pregnant WLWH and 6.4% (9/140) of non-pregnant women. The QG-MPH RS result CxCa was detected in 8.5% (8/94) of pregnant WLWH and in 12.1% (17/140) of non-pregnant women (Fig. 1). Next to these results, we noticed in 11.7% (11/94) of pregnant WLWH and in 42.1% (81/140) of non-pregnant WLWH a deviating combination of QG-MPH RS positivity leading to an inconsistent result for these samples.

**Fig. 2:**
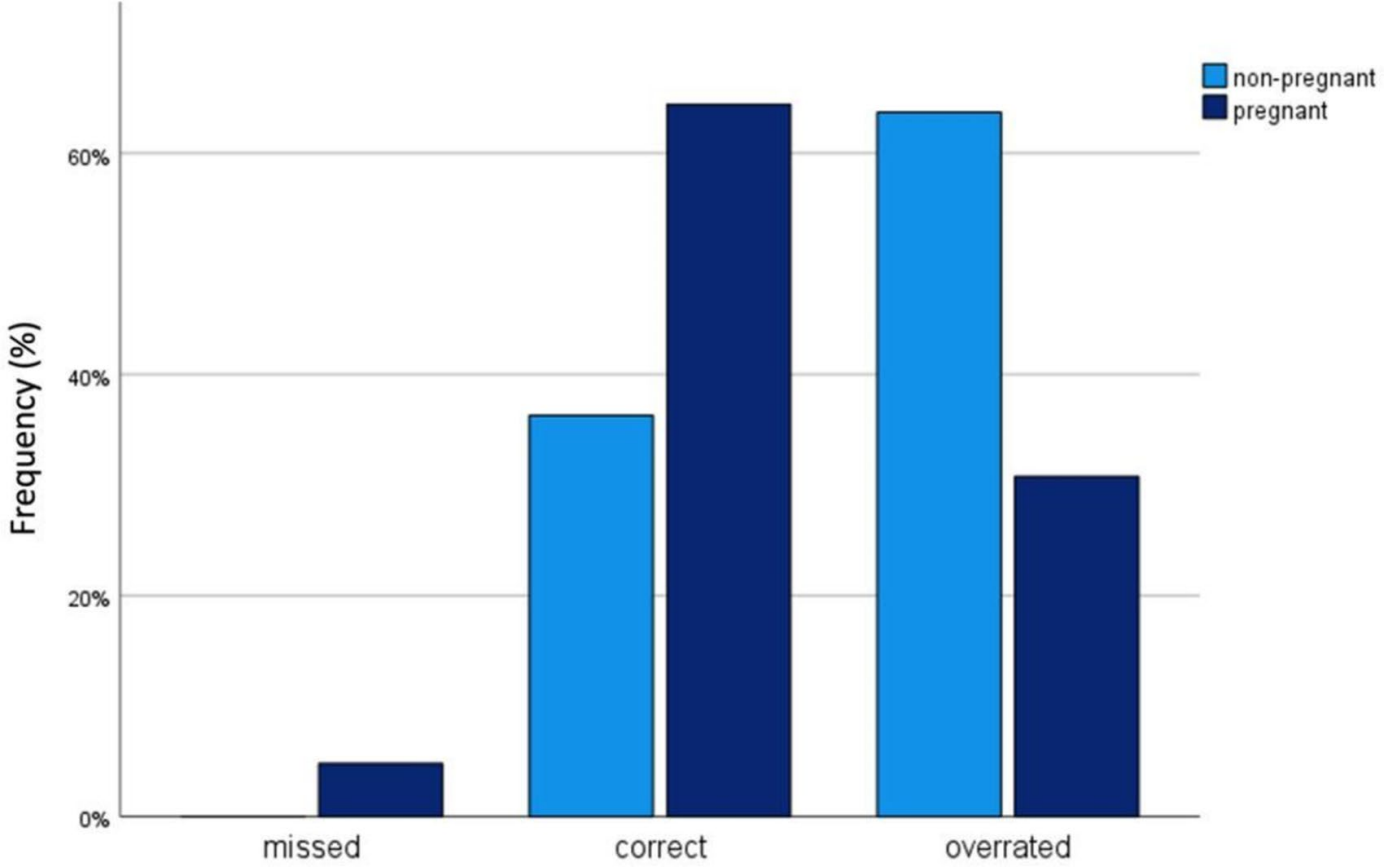
Concordance of the QG-MPH risk score (RS) results with the ASSIST Score results. QG-MPH RS results were compared to the ASSIST Score and categorized as missed, if the QG-MPH RS was lower than the ASSIST Score, as correct if it was the same and as overrated if it was higher than the ASSIST Score. The frequency is shown for non-pregnant WLWH (light blue; *n* = 135) and pregnant WLWH (dark blue; *n* = 85). Designed with IBM^®^ SPSS^®^ Statistics release 27.0 (SPSS Inc. an IBM Company, Chicago, Illinois, USA, 2020)

To evaluate the QG-MPH, RS results were compared to the dysplasia severity rated by the ASSIST Score. Findings were categorized as missed, if the QG-MPH RS was lower than the ASSIST Score, as correct if it was the same and as overrated if it was higher than the ASSIST Score. The QG-MPH missed 0% (0/135) of non-pregnant WLWH and 2.4% (2/85) of pregnant WLWH with a dysplasia indicated as a HCIN by the ASSIST Score (Fig. 2). Concordant results between the QG-MPH RS and the ASSIST Score, named in Fig. 2 as “Correct”, were found in 36.3% (49/135) of non-pregnant WLWH and in 67.1% (57/85) of pregnant WLWH. “Overrated” results from the QG-MPH RS are seen in 63.7% (86/135) of non-pregnant WLWH and in 30.6% (26/85) of pregnant WLWH.

For the detection of high-grade dysplasia, corresponding to an ASSIST Score of "HCIN", the QG-MPH RS showed a sensitivity of 53.9% and specificity of 82.4% among pregnant and non-pregnant WLWH.

When the QG-MPH RS detected “Inconsistent CIN II, CIN III, CxCa” results among non-pregnant WLWH, the ASSIST Score detected 74.6% (44/59) normal results without dysplasia, 20.3% (12/59) LCIN and 5.1% (3/59) HCIN. A similar distribution is also found in the group of pregnant WLWH with a QG-MPH RS result of “Inconsistent CIN II, CIN III, CxCa” results, where 88.9% (8/9) of the women showed normal findings in the ASSIST Score, and only 11.1% (1/9) had LCIN.

### HR-HPV detection in QG-MPH and MPG in comparison to the QG-MPH RS results

**Fig. 3:**
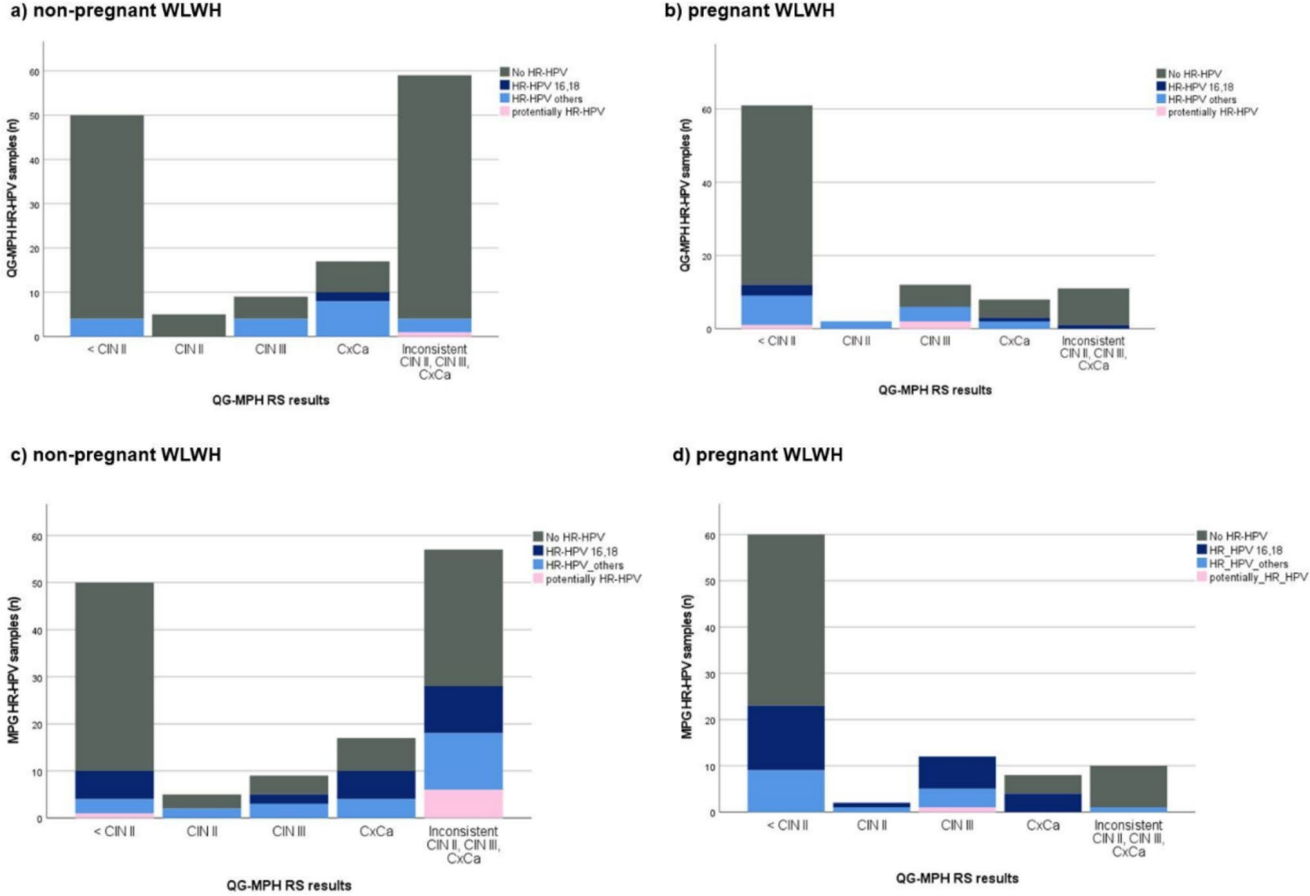
HR-HPV Detection by QG-MPH and MPG compared to the categorized samples by QG-MPH Risk Score (RS) results. The number of HPV-positive patients was plotted against the QG-MPH RS results. HR-HPV genotypes are divided into three groups: HR-HPV genotypes 16 or 18 (dark blue), HR-HPV-others 31, 33, 35, 39, 45, 51, 52, 56, 58, 59 (light blue), and potentially HR-HPV genotypes 66, 68a, 68b, 26, 53, 73, 82 (pink). **a** HR-HPV genotypes detection with QG-MPH assay among non-pregnant WLWH (*n* = 22). **b** HR-HPV genotypes detection with QG-MPH assay among pregnant WLWH (*n* = 24). **c** HR-HPV genotypes detection with the MPG among non-pregnant WLWH (*n* = 55). **d** HR-HPV genotypes detection with the MPG method among pregnant WLWH (*n* = 42). If multiple HPV genotypes could be detected in a sample, the one with the highest signal was evaluated. Designed with IBM^®^ SPSS^®^ Statistics release 27.0 (SPSS Inc. an IBM Company, Chicago, Illinois, USA, 2020)

The analysis in Fig. 3a–d respectively shows the HR-HPV detection by QG-MPH and MPG compared to the QG-MPH RS results in non-pregnant and pregnant WLWH. Examination of Fig. 3a and b reveals a higher frequency with 12.5% (3/12) of potentially HR-HPV genotypes present in pregnant WLWH compared to non-pregnant WLWH with 1.6% (1/61). This is particularly pronounced in cases where cervical dysplasia was indicated by the QG-MPH RS. In total, QG-MPH shows less HR-HPV-positive samples for both non-pregnant and pregnant WLWH. No difference in the detected HR-HPV genotypes is recognizable across the different QG-MPH RS results.

As clinically expected, with detection of dysplasia ≥ CIN II by the QG-MPH RS results, fewer HPV negative results are found in the QG-MPH assay and MPG method.

### Biomarker profile

**Fig. 4:**
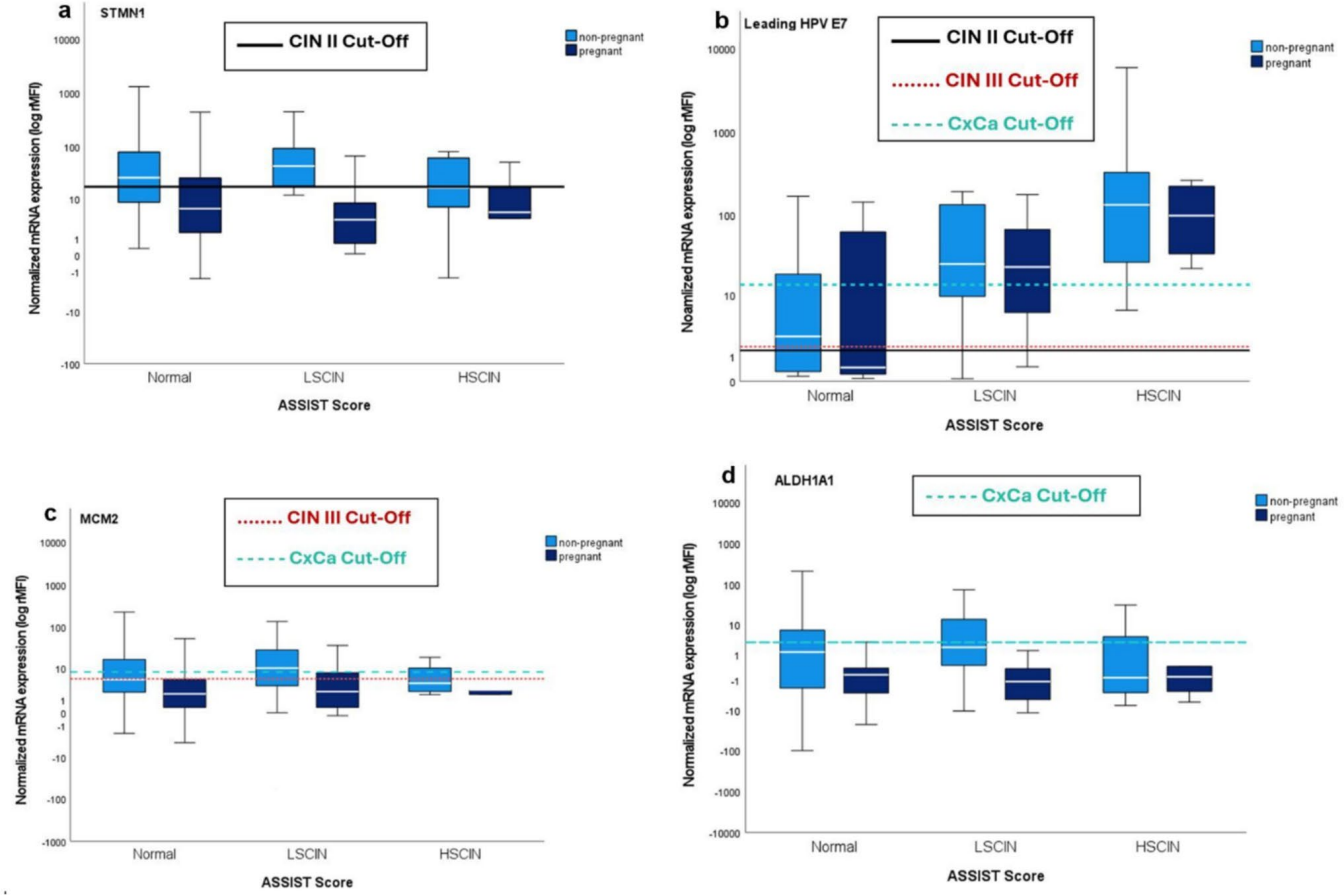
The mRNA expression levels of STMN1 (**a**), HPV E7 oncoprotein (**b**), MCM2 (**c**), and ALDH1A1 (**d**) are shown for different clinical stages as classified by the ASSIST Score. The normalized expression levels are presented as box-and-whisker plots. The MFI values are normalized to the reference gene ACTB, resulting in relative MFI (rMFI). Solid black lines represent the cut-off values used in the QG-MPH RS calculations for CIN II, the red lines for CIN III and the turquoise lines for CxCa. The analysis includes data from 140 non-pregnant WLWH (light blue) and 90 pregnant WLWH (dark blue). Designed with IBM^®^ SPSS^®^ Statistics release 27.0 (SPSS Inc. an IBM Company, Chicago, Illinois, USA, 2020)

The QG-MPH uses a distinct set of biomarkers to evaluate the risk for having a dysplasia with CIN II, CIN III or CxCa. The cut-off determination for the QG-MPH RS was performed on a HIV-negative, non-pregnant sample [26]. In the QG-MPH RS evaluation for this study, we found an increased number of overrated samples in both non-pregnant WLWH and pregnant WLWH compared to the dysplasia severity indicated by their ASSIST Score. To evaluate which biomarkers are responsible for this overrated result, the detected mRNA expression by QG-MPH was plotted against the ASSIST Score result for non-pregnant WLWH and pregnant WLWH. Shown are the results for the biomarkers Stathmin 1 (STMN1), E7 of the leading HPV genotype, MCM2, and ALDH1A1 (Fig. 4).

STMN1 is an oncoprotein and an integrated biomarker in the QG-MPH RS calculation for CIN II dysplasia, with a cut-off of 17.33 rMFI. As shown in Fig. 4a, the median STMN1 expression can be seen above the cut-off for CIN II in cases with a normal or LCIN dysplasia for non-pregnant WLWH while the median expression in cases with HCIN is below the cut-off for CIN II. For pregnant WLWH, the median mRNA expression for STMN1 is under the cut-off for all ASSIST Score grading.

The E7 expression of the leading HPV genotype does not show any major differences in expression between pregnant and non-pregnant WLWH. In the QG-MPH RS, the cut-off for E7 expression of the leading HPV genotype is 1.34 rMFI for CIN II, 1.40 rMFI for CIN III and 13.43 rMFI for CxCa. Figure 4b shows median expression of E7 above the cut-off values for CIN II and CIN III of patients with a HCIN as expected. Nevertheless, expression strength is also above the cut-off for CxCa despite no detection of CxCa in the cohorts. Also, in cases with a normal ASSIST Score, median mRNA expression is above the cut-off for CIN II and CIN III for non-pregnant WLWH. The same applies to the LCIN cases here for non-pregnant and pregnant WLWH.

MCM2 expression is shown in Fig. 4c with the cut-off values of 5.19 rMFI for CIN III and 7.96 rMFI for CxCa. Here, MCM2 expression among pregnant and non-pregnant WLWH is mostly placed below the cut-off values for both CIN III and CxCa, except for the LCIN among non-pregnant WLWH, where the median expression is situated above the cut-offs.

Biomarker expression of Aldehyde dehydrogenase (ALDH1) in pregnant and non-pregnant WLWH is mostly below the cut-off value of 2.99 rMFI for CxCa as shown in Fig. 4d. The fact that no CxCa indicators were found according to the ASSIST Score in our cohort shows that this biomarker may be for the most part usable in WLWH.

## Discussion

WLWH, whether pregnant or non-pregnant, are particularly susceptible to complications related to HPV due to the HIV-induced immunosuppression (1, 2). This compromised immune state not only increases the prevalence of HPV infections, but also accelerates progression to CIN and CxCa (4, 34). Current routine screening for cervical cancer prevention includes cytology, colposcopy with histology if necessary, and standard HPV tests. WLWH should be screened at a center with a high level of medical expertise proficient in colposcopy, and features substantial experience in managing cervical dysplasia in WLWH. Emerging mRNA-based biomarker tests, such as the QG-MPH assay, could offer potential advantages by providing minimally invasive methods to characterize cervical dysplasia lesions and its severity [18, 26].

These could be especially beneficial for high-risk groups like WLWH by enhancing prevention and early detection strategies. However, the applicability of these advanced biomarker tests in HIV-positive and pregnant women remains uncertain. The interaction between HIV, pregnancy, and biomarkers might alter the defined cut-offs within the QG-MPH RS, potentially leading to exaggeration of results. This study aims to evaluate the utility of the QG-MPH assay in detecting cervical dysplasia in HIV-positive, pregnant and non-pregnant women, while also exploring how HIV and pregnancy might influence the biomarker profiles used in the QG-MPH RS.

In our study, which involved 241 WLWH (96 were pregnant), we found that pregnant WLWH had a higher rate of abnormal cytology despite effective management of HIV with antiretroviral therapy. This indicates, as demonstrated in prior research, that physiological alterations during pregnancy might affect cervical cells and contribute to the advancement of CIN (1). When comparing HPV detection methods, the MPG method identified a higher percentage of HR-HPV genotypes compared to the QG-MPH. This discrepancy highlights the higher sensitivity of the MPG method for detecting HR-HPV. This higher sensitivity of the MPG method is expected as the MPG method is based on the detection of DNA and not on the detection of mRNA as is the QG-MPH method. It can be assumed, however, that a measurement based on mRNA will only detect the biologically relevant, replicating viruses, i.e., those that are also clinically relevant.

However, the QG-MPH assay demonstrates in our cohort high specificity for detecting HR-HPV genotypes and high-grade cervical dysplasia, respectively achieving 89.6% and 82.4% among pregnant and non-pregnant WLWH. Further, we detected an extended range of potentially carcinogenic HR-HPV genotypes (26, 53, 66, 68a, 68b, 73, 82) among pregnant WLWH when QG-MPH RS found cervical dysplasia. This result could be of critical importance as it could emphasize the need to incorporate these genotypes into routine screening procedures for pregnant immunosuppressed patients in order to better predict and manage the risk of cervical dysplasia.

German and WHO guidelines for cervical cancer prevention recommend that cervical biopsies among pregnant WLWH should be performed only when significant colposcopic abnormalities are observed (12, 13). The physiological changes during pregnancy, such as decidualization, complicate the biopsy procedure due to the risk of bleeding and preterm complications (27). Consequently, our study had limited histological data, necessitating the use of the ASSIST Score to estimate the stage of dysplasia based on cytological and colposcopic outcomes [21]. Additionally, a biopsy that was not clinically indicated was not approved by our ethics committee (ethical committees in Berlin, IRB number EA4/098/19), further limiting the availability of histological data. Nevertheless, the ASSIST Score provided a comprehensive assessment, even in the absence of histological findings [21]. Additionally, the challenges in colposcopy during cervical decidualization in pregnancy must be critically considered [27].

The QG-MPH test’s ability to detect mRNA-based biomarkers, such as STMN1, E7 oncoprotein, MCM2, and ALDH1A1, provides deeper insights into the biological status of CIN in WLWH. Each of these biomarkers has a specific role, and their expression can be influenced by HIV and pregnancy. STMN1, an oncoprotein that is crucial for cell cycle regulation, was observed to be overexpressed even in cases of normal findings or CIN I. This overexpression could be attributed to the influence of HIV, which is known to increase STMN1 levels due to phosphorylation (28, 35). Moreover, STMN1 plays a vital role during early pregnancy for embryonic implantation and decidualization, and its suppression by TNF-α during pregnancy complicates its interpretation in pregnant WLWH (36].

The E7 oncoprotein, central to HPV-induced carcinogenesis, showed no major differences in expression between pregnant and non-pregnant WLWH in our study. However, some cases exhibited E7 expression above the cut-off for cervical cancer despite only low or high-grade CIN being present (Fig. 4B).

This might be due to HIV proteins independently upregulating E7 expression, complicating its use as a diagnostic marker in HIV-infected individuals (29–31). MCM2, involved in DNA replication, is a key marker for various cancers (32, 33). In our cohort, MCM2 expression was generally below the cut-off for advanced CIN, suggesting that HIV infection might alter its expression, thus limiting its predictive value for CIN progression in WLWH. ALDH1A1, an early stem cell differentiation marker and a cancer marker, was mainly expressed below the cut-off for cervical cancer in our study, indicating no cases of cervical carcinoma [28]. Its role in the context of HIV or pregnancy is not well-documented, making its utility in predicting CIN or CxCa in WLWH unclear and warranting further research. However, in our cohort, we have no cases of cervical carcinoma.

These biomarkers offer a nuanced perspective on the risk and progression of CIN in WLWH. The influence of HIV and pregnancy on these markers emphasizes the intricate nature of diagnosing and treating CIN in this particular population. The QG-MPH test method's ability to quantify mRNA-based biomarkers provides an innovative approach to cervical dysplasia detection [18]. However, in our study, the QG-MPH RS detected cervical cancer in 8.5% (8/94) of pregnant women and 12.1% (17/140) of non-pregnant women living with HIV, figures that are notably higher than expected. Interpreting these biomarkers requires caution as the unique physiological and pathological contexts of HIV infection and pregnancy, as discussed above in detail, must be considered. These conditions may cause changes in biomarker levels that complicate result interpretation, making a tailored approach and possibly revised cut-off values necessary for this high-risk population.

Our study suggests that while the QG-MPH test shows potential, particularly in detecting a broader spectrum of high-risk HPV genotypes, its applicability and accuracy in women living with HIV require further validation.

Future research should focus on elucidating the roles of these biomarkers in different clinical settings, particularly in WLWH, to enhance the effectiveness of CIN management.

Despite these interesting findings, our study has several limitations. A larger and more comparable cohort, along with a follow-up and a control group, would have provided more robust data. Moreover, obtaining more histological results from biopsies would have allowed for a better evaluation and comparison with the QG-MPH RS results. Nevertheless, the ability of the QG-MPH assay to identify an extended range of potentially carcinogenic HR-HPV genotypes is particularly important for pregnant WLWH. This underscores the necessity of incorporating these HPV genotypes into routine screening protocols for immunocompromised patients in order to better predict and manage the risk of cervical dysplasia.

Our findings highlight the complexity of screening and diagnosing cervical dysplasia in WLWH and point to the need for tailored approaches that consider the unique challenges posed by HIV infection and pregnancy. While the QG-MPH test shows promise, with high specificity, its tendency to overestimate dysplasia severity in certain instances underscores the intricate interplay among HIV, pregnancy, and biomarker expression. Its use as a reliable diagnostic tool in WLWH requires further research and refinement.

## Supplementary Information

Below is the link to the electronic supplementary material.

Supplementary file1 (PDF 7999 kb)

## Data Availability

All available data is included in the paper.
