# Correction: Impact of HIV infection on cervical intraepithelial neoplasia detection in pregnant and non-pregnant women in Germany: a cross-sectional study

**DOI:** 10.1007/s00404-025-08217-x

**Published:** 2025-10-17

**Authors:** Irena Rohr, Anna Sophie Skof, Michaela Heinrich-Rohr, Fabian Weiss, Jan-Peter Siedentopf, Katharina von Weizsäcker, Irene Alba Alejandre, Wolfgang Henrich, Jalid Sehouli, Charlotte K. Metz

**Affiliations:** 1 Department of Obstetrics, Charité-Universitätsmedizin Berlin, Corporate Member of Freie Universität Berlin and Humboldt-Universität zu Berlin, Augustenburger Platz 1, 13353 Berlin, Germany; 2 HPV Research Laboratory, Department of Gynecology, Charité-Universitätsmedizin Berlin, Corporate Member of Freie Universität Berlin and Humboldt-Universität zu Berlin, Augustenburgerplatz 1, 13353 Berlin, Germany; 3 Duale Hochschule Schleswig-Holstein (DHSH), Hans-Detlev-Prien-Str. 10, 24106 Kiel, Germany; 4 Department of Obstetrics and Gynecology, University Hospital, Ludwig-Maximilians-University (LMU), Marchioninistrasse 15, 81377 Munich, Germany

**Correction: Archives of Gynecology and Obstetrics (2024) 310:3099–3110** 10.1007/s00404-024-07813-7

The article “Impact of HIV infection on cervical intraepithelial neoplasia detection in pregnant and non-pregnant women in Germany: a cross-sectional study”, written by Irena Rohr · Anna Sophie Skof · Michaela Heinrich‑Rohr · Fabian Weiss · Jan‑Peter Siedentopf · Katharina von Weizsäcker · Irene Alba Alejandre · Wolfgang Henrich · Jalid Sehouli and Charlotte K. Metz was originally published under exclusive license to The Author(s), under exclusive licence to Springer-Verlag GmbH Germany, part of Springer Nature 2024. As a result of the subsequent decision to publish the article under the open access model, the article’s copyright notice was changed on 25 September 2025 to © The Author(s) 2025 and the article is now distributed under a Creative Commons Attribution 4.0 International License, which permits use, sharing, adaptation, distribution and reproduction in any medium or format, as long as you give appropriate credit to the original author(s) and the source, provide a link to the Creative Commons licence, and indicate if changes were made. The images or other third party material in this article are included in the article’s Creative Commons licence, unless indicated otherwise in a credit line to the material. If material is not included in the article’s Creative Commons licence and your intended use is not permitted by statutory regulation or exceeds the permitted use, you will need to obtain permission directly from the copyright holder. To view a copy of this licence, visit http://creativecommons.org/licenses/by/4.0.

