# Supplementary material for: Impact of HIV infection on cervical intraepithelial neoplasia detection in pregnant and non-pregnant women in Germany: a cross-sectional study
Source: Arch Gynecol Obstet. 2024 Nov 7;310(6):3099–110. doi: 10.1007/s00404-024-07813-7 (PMC12534254; doi:10.1007/s00404-024-07813-7)
Supplement: Supplementary file 1 — Supplementary file1 (PDF 7999 kb) [file 404_2024_7813_MOESM1_ESM.pdf]

(12) INTERNATIONAL APPLICATION PUBLISHED UNDER THE PATENT COOPERATION TREATY (PCT)

(19) World Intellectual Property Organization
International Bureau

(43) International Publication Date
13 August 2020 (13.08.2020)

WIPO | PCT

(10) International Publication Number
WO 2020/161285 A1

(51) International Patent Classification:
C12Q 1/6886 (2018.01) *C12Q 1/70* (2006.01)

(21) International Application Number:
PCT/EP2020/053095

(22) International Filing Date:
07 February 2020 (07.02.2020)

(25) Filing Language: English

(26) Publication Language: English

(30) Priority Data:
19156203.2 08 February 2019 (08.02.2019) EP

(71) Applicant: **CHARITÉ - UNIVERSITÄTSMEDIZIN BERLIN** [DE/DE]; Chariteplatz 1, 10117 Berlin (DE).

(72) Inventor: **KAUFMANN, Andreas**; Lörracherstraße 2a, 12247 Berlin (DE).

(74) Agent: **HERTIN UND PARTNER RECHTS- UND PATENTANWÄLTE**; Kurfürstendamm 54/55, 10707 Berlin (DE).

(81) **Designated States** *(unless otherwise indicated, for every kind of national protection available)*: AE, AG, AL, AM, AO, AT, AU, AZ, BA, BB, BG, BH, BN, BR, BW, BY, BZ, CA, CH, CL, CN, CO, CR, CU, CZ, DE, DJ, DK, DM, DO, DZ, EC, EE, EG, ES, FI, GB, GD, GE, GH, GM, GT, HN, HR, HU, ID, IL, IN, IR, IS, JO, JP, KE, KG, KH, KN, KP, KR, KW, KZ, LA, LC, LK, LR, LS, LU, LY, MA, MD, ME, MG, MK, MN, MW, MX, MY, MZ, NA, NG, NI, NO, NZ, OM, PA, PE, PG, PH, PL, PT, QA, RO, RS, RU, RW, SA, SC, SD, SE, SG, SK, SL, ST, SV, SY, TH, TJ, TM, TN, TR, TT, TZ, UA, UG, US, UZ, VC, VN, WS, ZA, ZM, ZW.

(84) **Designated States** *(unless otherwise indicated, for every kind of regional protection available)*: ARIPO (BW, GH, GM, KE, LR, LS, MW, MZ, NA, RW, SD, SL, ST, SZ, TZ, UG, ZM, ZW), Eurasian (AM, AZ, BY, KG, KZ, RU, TJ, TM), European (AL, AT, BE, BG, CH, CY, CZ, DE, DK, EE, ES, FI, FR, GB, GR, HR, HU, IE, IS, IT, LT, LU, LV, MC, MK, MT, NL, NO, PL, PT, RO, RS, SE, SI, SK, SM, TR), OAPI (BF, BJ, CF, CG, CI, CM, GA, GN, GQ, GW, KM, ML, MR, NE, SN, TD, TG).

Published:
— *with international search report (Art. 21(3))*
— *with sequence listing part of description (Rule 5.2(a))*

(54) Title: A METHOD FOR DETERMINING THE SEVERITY OR GRADE OF HUMAN PAPILLOMAVIRUS (HPV)-INDUCED DYSPLASIA

(57) Abstract: The invention relates to an in vitro method for determining the severity or a grade of a human papillomavirus (HPV)-induced dysplasia or whether cervical carcinoma is present, and related materials, devices and computer-implementation of the method. The present invention comprises quantitatively determining an expression level of (i) viral and (ii) cellular messenger RNA (m RNA) in a sample obtained from the subject, wherein the determined viral m RNA encodes an HPV oncoprotein E6 and/or E7, and wherein the determined cellular m RNA comprises m RNA of at least one cellular proliferation marker, of at least one cancer stem cell marker, and of at least one tumor marker, and deducing from the quantity of said viral m RNA and said cellular m RNA the severity or a grade of the dysplasia or whether cervical carcinoma is present in the subject.

WO 2020/161285 A1

A METHOD FOR DETERMINING THE SEVERITY OR GRADE OF HUMAN PAPILLOMAVIRUS (HPV)-INDUCED DYSPLASIA

DESCRIPTION

The invention is in the technical field of in vitro molecular diagnostics. The invention relates to an in vitro method for determining the severity or a grade of a human papillomavirus (HPV)-induced dysplasia or whether cervical carcinoma is present, and related materials, devices and computer-implementation of the method.

The present invention therefore relates to an in vitro method for determining the severity or a grade of a human papilloma virus (HPV)-induced dysplasia or the presence of cervical carcinoma in a subject, comprising quantitatively determining an expression level of (i) viral and (ii) cellular messenger RNA (mRNA) in a sample obtained from the subject, wherein the determined viral mRNA encodes an HPV oncoprotein E6 and/or E7, and wherein the determined cellular mRNA comprises mRNA of at least one cellular proliferation marker, of at least one cancer stem cell marker, and of at least one tumor marker, and deducing from the quantity of said viral mRNA and said cellular mRNA the severity or a grade of the dysplasia or whether cervical carcinoma is present in the subject.

In some embodiments a method is provided for determining a human papilloma virus (HPV) infection in a subject and/or identifying the HPV genotype in a subject having an HPV infection and/or diagnosing the presence of a dysplasia in a subject having a HPV infection and/or determining the severity of the dysplasia in a subject. Determining means evaluating, detecting, and to determine the grading. In some embodiments, the invention enables grading of an HPV-induced dysplasia into a cervical intraepithelial neoplasia (CIN) grade based on molecular techniques. In some embodiments the invention enables prognosis of human papillomavirus (HPV)-induced dysplasia developing, or whether cervical carcinoma will develop, or whether a subject is at risk of developing cervical carcinoma from an HPV-induced dysplasia.

The present invention therefore relates to a method for the detection of precancerous and cancerous stages of anogenital dysplasia, in particular but not exclusively at cervical sites. The method is based on the quantitative evaluation of the expression of viral and cellular mRNA coding for validated biomarkers that are mis-regulated (preferably upregulated) in dysplastic tissue, which are selected and combined in the invention to assess the progression state of a malignant cell. These biomarkers comprise viral transcripts of oncogenes E6 and/or E7 of HPV genotypes (e.g. HPV6, 16, 18, 26, 31, 33, 35, 39, 45, 51, 52, 53, 56, 58, 59, 66, 68, 73, 82), viral spliced transcript markers, cellular transcripts of biomarkers like $p16^{ink4a}$ (syn. CDKN2A), proliferation markers mKi-67, MCM2, Topo2a, Stathmin (syn. Oncoprotein 18), stem cell markers p63, ALDH1A1, ALDH1L1, Sox2, Nanog, Pouf5FI (syn. Oct3/4), tumor markers BIRC5 (Syn. Survivin), TERT (human telomerase-reverse transcriptase), p53, squamocolumnar junction immunophenotype markers Keratin 7, Keratin 17, AGR2 (anterior gradient 2), CD63, GDA (Guanin Deaminase), MMP7 (Matrix Metalloproteinase 7), and cellular gene transcripts for normalization and as internal controls as indicated below.

The mRNA detection and quantification is utilized diagnostically for determining the progression stage of dysplasia, preferably by employing predetermined cut off values for the level of expression in a multiplex analysis.

BACKGROUND OF THE INVENTION

Cancer of the cervix uteri (cervical carcinoma) is the second most common malignant cancerous disease in women worldwide. It develops as a consequence of infections by high-risk Human Papillomaviruses (HR-HPV) via premalignant stages called cervical intraepithelial neoplasia (CIN) (Figure 1). Low-risk HPV induces dysplasia that rarely becomes invasive but is a common and disfiguring type of disease. Both groups of HPV can induce CIN1 lesions (mild dysplasia) where at most one third of the epithelium is altered. HR-HPV can induce higher grades of CIN, i.e. CIN2 (moderate dysplasia) with up to two thirds of the epithelium altered and CIN3 (severe or high-grade dysplasia) with an alteration of up to the entire epithelium. CIN2 and CIN3 have a considerable but still different risk for progression into invasive disease giving rise to carcinoma. Often these dysplastic stages are subsumed as CIN2+, however, for the present application it is important to consider the different risk for progression and clinical consequences of the different dysplastic stages that until now are subsumed in the same diagnostic category. In addition to HR-HPV infections other factors are involved in cervical carcinogenesis. Cellular progression to more transformed phenotypes is associated with alterations in viral and cellular gene expression. Certain biomarker proteins are used by pathologists to better identify and describe the stage of underlying dysplasia, such as immunohistological staining for p16, Ki-67, or Stathmin-1 (Oncoprotein 18) (Figure 2).

Cervical cancer and pre-cancer screening relies on cytological sampling from the epithelial surface of the cervix uteri. Cellular morphological alterations are detected microscopically by the PAP test (cytology according to Papanicolaou). This subjective method has a high failure rate because few altered cells have to be identified and evaluated in a background of numerous normal cells by cytopathologists. The weakness of the PAP test results in a sensitivity of 53% for the detection of CIN2+, while the specificity is 96.3% (Cuzick 2006 Int. J. Cancer, 119:1095-1101).

The association of true CIN and cervical cancer with HR-HPV infection, apart from very few exceptions, led to the development and introduction of molecular test methods for screening. These methods most often detect viral nucleic acid (DNA) by PCR-based amplification (e.g., Cobas by Roche, Onclarity by BectonDickinson) and few by probe-based hybridization techniques (e.g., HybridCaptureII by Qiagen, Cervista by Hologic). Also amplifying techniques to detect viral mRNA have been developed (e.g. Aptima by Hologic). Recently, oncoprotein-based immunological assays have been developed (e.g. OncoE6 Cervical Test by Arbor Vita; recomwell HPV E7 by Mikrogen). Initially, HR-HPV was detected as a pool of 12 to 14 genotypes classified by the WHO as carcinogenic.

The invention of molecular tests for the detection of HPV and the establishment of molecular markers correlating with dysplasia were important steps in the amelioration of modern screening algorithms. Since HR-HPV infections have been shown to be carcinogenic and the prerequisite of cellular transformation in almost all cervical lesions and cancers, the screening can now specifically target the pathology’s origin.

PCR-based HPV DNA tests display a sensitivity of above 95% and a specificity of approximately 90% for the detection of CIN2+. It is problematic, however, that the positive predictive value for detecting histologically confirmed dysplasia (CIN2+) is only about 20% (Cuzick 2006 Int. J. Cancer, 119:1095-1101). This is due to many women being infected by HPV without developing any dysplastic lesions. These women would not need a close follow up or further diagnostic procedures despite testing positive for HPV. There is a need for improved screening methods that can discriminate infection from CIN, and within the continuum of progression those that need therapy or not, and identify patients with invasive carcinoma.

Therefore, a screening program cannot be based on mere HPV detection. In a second step, a diagnosed HPV infection would have to be characterized for its potential of transformation. The official screening programs now offer HPV testing as a first step, but then refer HPV-positive patients to the traditional diagnostics of PAP smears and colposcopy. Up to now, molecular markers that have been shown to correlate with transformation and to describe the biological process of dysplasia are only used as add-ons for cytology and histology. As described above, very recent innovations in HPV diagnostics integrate single markers, such as the viral oncoproteins E6 and E7 into HPV detection representing a first movement towards molecular characterization and thereby risk stratification of underlying HPV infections.

Other innovations are specific promoter methylation signatures and microRNA expression that correlate with progressed stages. However, detection of such alterations is cumbersome (e.g., HPV-risk assay by Self-Screen B.V., The Netherlands; or GynTect by Oncgnostics, Germany).

With the insight that persistent infection by HPV genotypes is a prerequisite for dysplasia development, and that different risk of progression to later CIN stages or cervical cancer is conferred by different genotypes, HPV genotype discriminating methods and determination of CIN stage are becoming central to cervical cancer diagnostics and prognostics. Genotyping methods for the most carcinogenic types (like HPV16 or HPV18), but also full genotype identification of individual 14 to 18 HR-HPV for epidemiological studies and characterization of multiple infections have been developed and have shown advantages over pooled HR-HPV detection.

Evans et al ("HPV E6/E7 RNA In Situ Hybridization Signal Patterns as Biomarkers of Three-Tier Cervical Intraepithelial Neoplasia Grade", PLoS ONE, vol. 9, no. 3, 2014) teaches combined in situ hybridization of E6/E7 and p16 mRNAs in different CIN grades. US2017/204481 teaches detection of HPV E6/E7 and p16 mRNA in clinical sample of cervical neoplasia using flow-cytometry-based methods. The combination of markers employed in the present invention is not disclosed and in situ hybridization and flow-cytometry represent more technically challenging and less straightforward approaches compared to the quantitative molecular tools employed in the present invention.

US 2004/202996 teaches methods of categorizing HPV-induced cervical neoplasia and cancer in a subject, comprising detecting expression of HPV nucleic acid levels and detecting a protein marker expression in the sample. Dual determination of nucleic acids and protein also represents a significantly more laborious method compared to the present invention. Wang et al. ("Diagnostic performance of HPV E6/E7, hTERT, and Ki67 mRNA RT-qPCR assays on formalin-fixed paraffin embedded cervical tissue specimens from women with cervical cancer", Experimental and Molecular Pathology Press, vol. 98, no. 3, 2015) teaches the combined mRNA determination of E6/E7 mRNA, TERT mRNA and Ki67 mRNA in cervical tissue samples. The combination of

markers employed in the present invention is not disclosed therein and reversal of paraffin embedding is a possible but sub-optimal approach to diagnosis/prognosis in a clinical setting.

Despite these advancements, there remains a need in the art for reliable, accurate and straightforward means for determining HPV infection, and more importantly determining the grade or severity of an HPV-induced dysplasia, or whether cervical cancer is evident, especially from a routine cervical smear sample using a technically versatile and simple methodology, thereby enabling an improved prognosis of risk for cervical cancer and/or more effective treatment management.

SUMMARY OF THE INVENTION

In light of the difficulties in the prior art, the technical problem underlying the present invention is the provision of improved or alternative means for determining the grade or severity of an HPV-induced dysplasia, or whether cervical cancer is evident. A further object of the invention is the provision of means that reliably and simultaneously can detect HR-HPV infection and identify the specific genotype(s), identify prevalent dysplasia, discriminate dysplastic stages for therapy decision, and identify invasive disease of cervical carcinoma.

The solution to the technical problem of the invention is provided in the independent claims. Preferred embodiments of the invention are provided in the dependent claims.

The invention therefore relates to an in vitro method for determining the severity or a grade of a human papilloma virus (HPV)-induced dysplasia or the presence of cervical carcinoma in a subject, comprising:

a) quantitatively determining an expression level of (i) viral and (ii) cellular messenger RNA (mRNA) in a sample obtained from the subject, wherein
    i) the determined viral mRNA encodes an HPV oncoprotein E6 and/or E7, and
    ii) wherein the determined cellular mRNA comprises:
        - mRNA of at least one cellular proliferation marker,
        - mRNA of at least one cancer stem cell marker, and
        - mRNA of at least one tumor marker, and
b) deducing from the quantity of said viral mRNA and said cellular mRNA the severity or a grade of the dysplasia or whether cervical carcinoma is present in the subject.

The invention is therefore characterized by the combined quantitative measurement of viral and cellular (human/host) messenger RNA transcripts of established marker molecules. The assessment of this combination of markers in a quantitative manner enables reliable determination of dysplasia severity or grading (i.e. CIN grading) or the determination of the presence of cervical carcinoma. This method therefore enables avoiding examination of cytological samples from the epithelial surface of the cervix uteri, as commonly obtained from and assessed microscopically in a PAP test.

The cellular proliferation markers, cancer stem cell markers and tumor markers are known to a skilled person and may be selected and employed without undue effort. Each of these marker groups, and preferred markers, are provided in detail below.

The unique combination of markers, comprising an HPV oncoprotein E6 and/or E7, a proliferation marker, cancer stem cell marker and tumor marker, enables an accurate and reliable determination of the severity or grading of a dysplasia, or determining whether cervical carcinoma is present.

In one embodiment, the method comprises normalizing the determined expression level of the viral and cellular mRNA to the expression level of the at least one housekeeping gene. The invention therefore encompasses assessment of absolute or normalized expression levels of the markers in a quantitative fashion. Normalized levels considering the expression of a housekeeping gene is preferred.

In one embodiment of the method, the severity or grade of dysplasia is determined by comparing the quantified (absolute and/or normalized) expression levels of viral and cellular mRNA to predetermined threshold values for severities or grades of HPV-induced dysplasia and the presence of cervical carcinoma.

In one embodiment of the method, quantified (absolute and/or normalized) expression levels of viral and cellular mRNA indicate a severity or grade of dysplasia or the presence of cervical carcinoma when said levels of mRNA are above predetermined statistically established threshold values, wherein an expression level

- above a first threshold corresponds to stage CIN2,
- above a second threshold corresponds to stage CIN3, and
- above a third threshold corresponds to cervical carcinoma.

In one embodiment of the method, the viral mRNA encoding an HPV oncoprotein E6 and/or E7 is selected from the group consisting of spliced mRNA E6*I, E1C, and E1^4.

In one embodiment of the method, the viral mRNA encoding an HPV oncoprotein E6 and/or E7 is selected from the group consisting of a HPV genotype selected from the group comprising HPV 6, 16, 18, 26, 31, 33, 35, 39, 45, 51, 52, 53, 56, 58, 59, 66, 68, 73 and 82.

In one embodiment of the method, viral mRNA encoding HPV E6 and/or E7 is determined for multiple potential HPV genotypes (infections), and the strongest HPV genotype is employed in deducing the severity or grade of the dysplasia, wherein the strongest HPV genotype is the only HPV genotype detected in the assay or the HPV genotype with the highest expression when multiple HPV genotypes (infections) are present.

In one embodiment of the method, at least one cellular proliferation marker is selected from the group consisting of P16, MCM2, Topo2a, STMN1, Ki-67 (mKi-67), preferably consisting of P16, STMN1 and MCM2.

In one embodiment of the method, at least one cancer stem cell marker is selected from the group consisting of Sox, Nanog, POU5FI/Oct3/4, ALDH1A1 and ALDH1L1, preferably ALDH1A1.

In one embodiment of the method, the at least one tumor marker is selected from the group consisting of BIRC5, TERT and p53, preferably BIRC5 and TERT.

In one embodiment of the method, the following markers are employed to determine a CIN2+ grade of dysplasia: strongest HPV genotype, HPV16 E6*I, HPV16 E1^E4, CDKN2A/P16, and STMN1.

In one embodiment of the method, the following markers are employed to determine a CIN3+ grade of dysplasia: strongest HPV genotype, HPV16 E6*I, HPV 16 E1^E4, P16 and MCM2.

In one embodiment of the method, the following markers are employed to determine the presence of cervical carcinoma: strongest HPV genotype, BIRC5, TERT, ALDH1A1 and MCM2.

As is demonstrated in the examples and detailed description below, the specific markers mentioned above, and in particular the combinations of the specific markers mentioned above, enable an accurate and reliable determination of dysplasia severity or stage, or whether cervical carcinoma is present. The method may therefore be defined in some embodiments by the specific markers employed for each of the cellular proliferation marker, cancer stem cell marker and tumor marker. In preferred embodiment, the specific markers mentioned under these terms may be combined.

The method may in some embodiments be defined by the selection of markers employed for each CIN stage. A key CIN stage determination is, for example, the determination between CIN stage 2, and any higher stage, for example either CIN3 or the presence of carcinoma. As is disclosed in detail below, the inventor has identified particular groups of markers elected from the groups of cellular proliferation markers, cancer stem cell markers and tumor markers, which enable especially reliable determination of CIN2 stage, CIN3 stage or the presence of cancer. Combinations of these stage-specific markers may therefore in some embodiments be preferred. In some embodiments, the same markers are employed in determining CIN2, CIN3 and/or the presence of carcinoma.

In a preferred embodiment, the in vitro method comprises:

a) quantitatively determining an expression level of (i) viral and (ii) cellular messenger RNA (mRNA) in a sample obtained from the subject, wherein

   i. the determined viral mRNA encodes an HPV oncoprotein E6 and/or E7, selected from the group consisting of the strongest HPV genotype, HPV16 E6*I and HPV16 E1^E4, wherein the strongest HPV genotype is the only HPV genotype or the HPV genotype with the highest expression level when multiple HPV genotypes (infections) are present, and

   ii. the determined cellular mRNA comprises:

      - mRNA of at least one cellular proliferation marker, selected from the group consisting of P16, STMN1 and MCM2, and

      - mRNA of at least one cancer stem cell marker, including ALDH1A1, and

      - mRNA of at least one tumor marker, selected from the group consisting of BIRC5 and TERT, and

      - mRNA of at least one housekeeping gene, and

b) deducing from the quantity of said viral mRNA and said cellular mRNA the severity or a grade of the dysplasia or whether cervical carcinoma is present in the subject, wherein

i. the following markers are employed to determine a CIN2+ grade of dysplasia: strongest HPV genotype, HPV16 E6*I, HPV16 E1^E4, P16, and STMN1,

ii. the following markers are employed to determine a CIN3+ grade of dysplasia: strongest HPV genotype, HPV16 E6*I, HPV 16 E1^E4, P16 and MCM2, and

iii. the following markers are employed to determine the presence of cervical carcinoma: strongest HPV genotype, BIRC5, TERT, ALDH1A1 and MCM2,

wherein

c) the severity or grade of dysplasia is determined by comparing the quantified expression levels of viral and cellular mRNA, preferably normalized to the expression level of the housekeeping gene, to predetermined thresholds for severities or grades of HPV-induced dysplasia and the presence of cervical carcinoma.

The above-described embodiment represents one specific and preferred embodiment of the method, demonstrated to enable effective determination of dysplasia grade or the presence of carcinoma.

In one embodiment, the method comprises quantitatively determining an expression level of an HPV oncoprotein E6 and/or E7, selected from the group consisting of the strongest HPV genotype, HPV16 E6*I and HPV16 E1^E4, wherein the strongest HPV genotype is the only HPV genotype or the HPV genotype with the highest expression level when multiple HPV genotypes (infections) are present, and mRNA of P16, STMN1 and MCM2, ALDH1A1, BIRC5 and TERT, wherein the expression level of each marker is compared to predetermined threshold values for severities or grades of HPV-induced dysplasia and the presence of cervical carcinoma.

In one embodiment of the method, the quantitative determining of the expression of viral and cellular mRNA in the sample of a subject is performed as single step method.

In one embodiment of the method, the quantitative determining of the expression of viral and cellular mRNA in the sample of a subject is performed using solid phase-bound probe-directed capture of a target mRNA, preferably QuantiGene, or RT-qPCR. As shown in the examples, multiple quantitative molecular techniques suitable for assessing mRNA levels may be employed in order to carry out the invention. Preferred methods are discussed in detail below.

In one embodiment of the method, the method comprises additionally determining the HPV type in said subject having an HPV infection.

In one embodiment of the method, the method comprises additionally predicting the risk of the subject developing cervical carcinoma. Preferred embodiments of risk prediction are discussed below and supported by the examples. The prognostic approach of the present invention encompasses in some embodiments a determination of risk of a subject developing cervical carcinoma by determining e.g. the type and/or stage of dysplasia. For example, by determining the stage or type of HPV-induced dysplasia, effective predictive statement may be made regarding cancer risk. In other embodiments, the invention encompasses a predictive method in

which the various markers are assessed and compared to established threshold values for particular risk groups.

In one embodiment of the method, the amount of viral and cellular mRNA, preferably the normalized expression level of said viral and cellular mRNA, is introduced into a mathematical algorithm that combines said amounts and provides a score value suitable for determining the severity and/or a grade of the dysplasia or whether cervical carcinoma is present in the subject.

A further aspect of the invention relates to a computer readable storage medium, associated software and other computer-implementation of the method described herein.

In one aspect, a computer readable storage medium is provided, comprising instructions to configure a processor to perform a method and/or algorithm of mathematical evaluation for the generation of a mathematical model based on the quantitative mRNA expression levels determined in a method according to any one of the preceding claims for the characterization of a sample based on evaluation of the mRNA expression of the markers, wherein characterization means analysis and/or retrieving predictive information and/or profiling based on molecular mRNA expression and/or comparing results of quantitative mRNA expression analysis according to a standard.

In one embodiment of the computer readable medium, dichotomization is used to determine cut-off values for the severities or grades of HPV-induced dysplasia or the presence of cervical carcinoma according to the method described herein, comprising grouping samples into clinical groups/clinical scores.

In one embodiment of the computer readable storage medium, the medium is suitable for analyzing data obtained from cervical smear samples, and wherein the clinical groups/clinical scores comprise:

- Clinical group 0, is defined as HPV negative and histologically without pathological findings,
- Clinical group 1 is defined as HPV positive and histologically without pathological findings,
- Clinical group 2 is defined as HPV positive and histologically CIN1 and
- Clinical group 3 is defined as HPV positive and histologically CIN2 and
- Clinical group 4 is defined as HPV positive and histologically CIN3 and
- Clinical group 5 is defined as HPV positive and histologically cancerous,

wherein these clinical groups are used for dichotomization into clinical thresholds,

- particularly wherein clinical threshold CIN2+ is defined as separating the clinical groups 0-2 from the clinical groups 3-5, and
- clinical threshold CIN3+ is defined as separating the clinical groups 0-3 from the clinical groups 4-5, and
- the clinical threshold of carcinomas is defined as separating the clinical groups 0-4 from the clinical group 5.

In one embodiment, the computer readable storage medium comprises instructions to configure a processor to perform a method and/or algorithm of mathematical evaluation for the processing of quantitative mRNA expression levels (based on raw or relativized data) obtained by a method as described herein, wherein predictive values are calculated for the respective markers that exceed the predictive value of single marker cut-offs, and a risk stratification score is calculated based on the quantities of the cellular and viral mRNA and a mathematical evaluation combining the predictive values of analyzed cellular and viral markers, preferably wherein said marker values are dichotomized into cut-off values for the stratification of the risk of the subject to develop a malignant transformation.

Further aspects of the invention:

In further aspects, the invention relates to:

I. An in vitro method of detecting (a) the presence and/or (b) the type of HPV mRNA in a sample obtained from a subject, and/or deducing (c) the presence and/or (d) severity or grade of a HPV-induced dysplasia in said subject, and/or (e) deducing the presence of an invasive disease of cervical carcinoma, wherein said in vitro method comprises the following steps:

a) quantitative determination of the expression of (i) viral and (ii) cellular mRNA in said sample of a subject, wherein

i) the viral mRNA is the mRNA of at least one HPV oncoprotein E6 and/or E7, wherein the HPV oncoprotein E7 and/or E6 mRNA is derived from an HPV type selected from the group comprising HPV types 6, 16, 18, 26, 31, 33, 35, 39, 45, 51, 52, 53, 56, 58, 59, 66, 68, 73, and 82, and

ii) wherein the cellular mRNA is mRNA of

- at least one cellular biomarker selected from the group comprising $p16^{ink4a}$, MCM2, Topo2a, Stathmin/oncoprotein 18, mKi-67 (Ki 67), and/or
- mRNA of at least one tumor stem cell marker selected from the group comprising Sox, Nanog, POU5FI/Oct3/4, ALDH1A1, and/or ALDH1L1, and/or
- mRNA of at least one marker of the immune phenotypes selected from the group comprising MMP7, AGR2, GDA, Keratin 7, Keratin 17, CD63, and/or p63, and/or
- mRNA of at least one tumor marker selected from the group comprising BIRC5/Survivin, Telomerase (TERT), and p53, and
- mRNA of at least one housekeeping gene (e.g. those described throughout the present disclosure),

b) deducing from the presence and/or quantity of said viral mRNA and said cellular mRNA (a) whether a HPV infection is present in said subject and/or (b) the type of HPV in said subject having a HPV infection and (c) the presence of a HPV-induced dysplasia in said subject having a HPV infection and (d) the severity or grade of the dysplasia in a subject having a HPV-induced dysplasia and (e) whether invasive disease of cervical carcinoma is present.

Said method, which is described in more detailed embodiments below can be used to classify and/or triage patients in accordance with the deduced presence, severity or grade of dysplasia so that patients can be most efficiently therapeutically treated.

II. The in vitro method according to aspect I), wherein additionally the presence and quantity of HPV spliced mRNA markers (E6*I, E1C, E1^4) is determined, wherein the HPV spliced mRNA is selected from the group comprising the HPV types 6, 16, 18, 26, 31, 33, 35, 39, 45, 51, 52, 53, 56, 58, 59, 66, 68, 73, and/or 82.

III. The in vitro method according to aspects I) and/or II), wherein said quantitative determination of said mRNA of at least one housekeeping gene is used for the relativization of said quantities of viral and cellular mRNA to the cellularity in said sample of said subject and wherein said housekeeping gene is selected from the group comprising beta-Actin, UBC, EIF4E2, and HPRT1, particularly from beta-Actin and/or UBC.

IV. The in vitro method according to any of the preceding aspects, wherein said sample is selected from the group comprising smears from a body surface, cytological smears, fine needle aspirates, body excretions, blood or serum samples, tissue biopsies, fresh frozen or formalin-fixed paraffin-embedded tissue materials (FFPE) and cultured cellular material, particularly smears from body surfaces.

V. The in vitro method according to any of aspects I) to IV), wherein the viral and/or cellular mRNA is selected from the group comprising:

a) mRNA of at least one HPV oncoprotein E6 and/or E7, and/or E1C, and/or E1^E4

b) mRNA of $p16^{ink4a}$, Ki-67, Stathmin, MCM2, Topo2A, BIRC5, TERT, ALDH1A1, and/or Keratin 17.

VI. The in vitro method according to any of aspects I) to V), wherein the quantitative expression of viral and cellular mRNA comprises the following steps:

- providing a device or a set of reagents comprising:
    - a probe-directed capture molecule specifically hybridizing to and allowing quantitative detection of mRNA of at least one HPV oncoprotein E6 and/or E7, wherein the HPV oncoprotein E7 and/or E6 mRNA is derived from the group comprising the HPV types 6, 16, 18, 26, 31, 33, 35, 39, 45, 51, 52, 53, 56, 58, 59, 66, 68, 73, and 82, and
    - a probe-directed capture molecule specifically hybridizing to and allowing quantitative detection of mRNA of at least one of the following cellular marker:

        a) mRNA of at least one cellular biomarker selected from the group comprising $p16^{ink4a}$, MCM2, Topo2a, Stathmin/oncoprotein 18, and/or mKi-67 (Ki-67), and/or

b) mRNA of at least one tumor stem cell marker selected from the group comprising Sox, Nanog, POU5FI/Oct3/4, ALDH1A1, and/or ALDH1L1, and/or

c) mRNA of at least one marker of the immune phenotypes selected from the group comprising MMP7, AGR2, GDA, Keratin 7, Keratin 17, CD63, and/or p63, and/or

d) mRNA of at least one tumor marker selected from the group comprising BIRC5/Survivin, Telomerase (TERT), and p53, and

e) mRNA of at least one housekeeping gene,

- bringing said device or said set of reagents in contact with a sample of said subject under conditions allowing hybridization (e.g. QuantiGene method, RT-qPCR or other methods involving a hybridization step) of said above-mentioned probe-directed capture molecules with the mRNA present in said sample of said subject
- determining quantitatively the viral mRNA of said at least one HPV oncoprotein E6 and/or E7 and of said cellular mRNA as defined in a) to e).

VII. A method according to any of aspects I) to VI), wherein the detection of quantitative expression of viral and cellular mRNA comprising the following steps:

- providing a device or a set of reagents comprising:
- at least one or more probe-directed capture molecule(s) specifically hybridizing to and allowing quantitative detection of mRNA of at least one HPV oncoprotein E6 and/or E7 and
- a probe-directed capture molecule specifically hybridizing to and allowing quantitative detection of mRNA of p16^{ink4a}, and/or
- a probe-directed capture molecule specifically hybridizing to and allowing quantitative detection of mRNA of Ki-67, and/or
- a probe-directed capture molecule specifically hybridizing to and allowing quantitative detection of mRNA of Stathmin, and/or
- a probe-directed capture molecule specifically hybridizing to and allowing quantitative detection of mRNA of MCM2, and/or
- a probe-directed capture molecule specifically hybridizing to and allowing quantitative detection of mRNA of Topo2A, and/or
- a probe-directed capture molecule specifically hybridizing to and allowing quantitative detection of mRNA of BIRC5, and/or
- a probe-directed capture molecule specifically hybridizing to and allowing quantitative detection of mRNA of ALDH1A1, and/or
- optionally a probe-directed capture molecule specifically hybridizing to and allowing quantitative detection of mRNA of TERT, and/or

- a probe-directed capture molecule specifically hybridizing to and allowing quantitative detection of mRNA of P53.

VIII. A method according to any of aspects I) to VII), wherein the quantitative detection of the expression of viral and cellular mRNA in the sample of a subject is performed as single step method.

IX. A method according to any of aspects I) to VIII), wherein the quantitative determination of the expression of viral and cellular mRNA in the sample of a subject includes the evaluation of a negative control in each assay, preferably a set of three.

X. A method according to any of aspects I-IX), wherein the absolute and/or relative amount of HPV mRNA of at least one HPV oncoprotein E6 and/or E7 is/are correlated with the presence of HPV infection and/or severity or grade of dysplasia, and provided said subject has a HPV infection, determining if said relative amount of HPV mRNA of said at least one HPV oncoprotein E6 and/or E7 is/are above a predetermined statistically established threshold value.

XI. A method according to any of aspects I) to X), wherein the test result of said subject is to be considered positive for at least one HPV genotype infection when the non-relativized (raw) mRNA level (MFI) of said sample is:

- above a threshold 1 that is preferably the median of MFI-signals of the set of one, two or three negative controls for the HPV-genotype 6, only as an example, the threshold may be defined as 35.25 MFI, and/or
- above a threshold 2 that is preferably the median of MFI-signals of the set of three negative controls for the HPV-genotype 16, only as an example, the threshold may be defined as 32.25 MFI , and/or
- above a threshold 3 that is preferably the median of MFI-signals of the set of three negative controls for the HPV-genotype 18, only as an example, the threshold may be defined as 54.75, and/or
- above a threshold 4 that is preferably the median of MFI-signals of the set of three negative controls for the HPV-genotype 26, only as an example, the threshold may be defined as 59.50, and/or
- above a threshold 5 that is preferably the median of MFI-signals of the set of three negative controls for the HPV-genotype 31, only as an example, the threshold may be defined as 31.75, and/or
- above a threshold 6 that is preferably the median of MFI-signals of the set of three negative controls for the HPV-genotype 33, only as an example, the threshold may be defined as 28.25, and/or
- above a threshold 7 that is preferably the median of MFI-signals of the set of three negative controls for the HPV-genotype 35, only as an example, the threshold may be defined as 33.75, and/or

- above a threshold 8 that is preferably the median of MFI-signals of the set of three negative controls for the HPV-genotype 39, only as an example, the threshold may be defined as 28.75, and/or
- above a threshold 9 that is preferably the median of MFI-signals of the set of three negative controls for the HPV-genotype 45, only as an example, the threshold may be defined as 64.75, and/or
- above a threshold 10 that is preferably the median of MFI-signals of the set of three negative controls for the HPV-genotype 51, only as an example, the threshold may be defined as 38.75, and/or
- above a threshold 11 that is preferably the median of MFI-signals of the set of three negative controls for the HPV-genotype 52, only as an example, the threshold may be defined as 27.75, and/or
- above a threshold 12 that is preferably the median of MFI-signals of the set of three negative controls for the HPV-genotype 53, only as an example, the threshold may be defined as 26.75, and/or
- above a threshold 13 that is preferably the median of MFI-signals of the set of three negative controls for the HPV-genotype 56, only as an example, the threshold may be defined as 29.25, and/or
- above a threshold 14 that is preferably the median of MFI-signals of the set of three negative controls for the HPV-genotype 58, only as an example, the threshold may be defined as 25.25, and/or
- above a threshold 15 that is preferably the median of MFI-signals of the set of three negative controls for the HPV-genotype 59, only as an example, the threshold may be defined as 26,75, and/or
- above a threshold 16 that is preferably the median of MFI-signals of the set of three negative controls for the HPV-genotype 66, only as an example, the threshold may be defined as 40.75, and/or
- above a threshold 17 that is preferably the median of MFI-signals of the set of three negative controls for the HPV-genotype 68, only as an example, the threshold may be defined as 23.75, and/or
- above a threshold 18 that is preferably the median of MFI-signals of the set of three negative controls for the HPV-genotype 73, only as an example, the threshold may be defined as 55.50, and/or above a threshold 19 that is preferably the median of MFI-signals of the set of three negative controls for the HPV-genotype 82, only as an example, the threshold may be defined as 31.25.

As known in the art, the determination of the threshold value generally depends on the type of method that is used, very often also under specific products that are used, which means that referring to a specific threshold value would not be appropriate. This does not mean that a person of skill in the art would not be able to determine a respective

threshold value taking into account the method that is used, the reagents that are used, available controls to determine a reasonable threshold value and the like.

XII. A method according to any of aspects I) to XI), wherein a normalized amount of HPV mRNA of at least one HPV oncoprotein E6 and/or E7 is correlated with the presence of HPV infection or severity or grade of dysplasia, and wherein dysplasia is present if said normalized amount of HPV mRNA of at least one HPV oncoprotein E6 and/or E7 is above a predetermined statistically established threshold value in said subject, wherein

- above a Threshold 1 corresponds to a mRNA level found in cervical intraepithelial neoplasia (CIN) stage CIN1, and
- above a Threshold 2 corresponds to a mRNA level found in CIN2, and
- above a Threshold 3 corresponds to a mRNA level found in CIN3, and
- above a Threshold 4 corresponds to a mRNA level found in cancer.

It is noted that the severity of grade of dysplasia is advantageously also determined in consideration of the expression of cellular biomarkers, e.g. p16, STMN, etc., which are expressed above a respectively predetermined statistically established threshold value.

XIII. A method according to any of aspects I) to XII), wherein the severity of dysplasia is determined by the quantified normalized amount of HPV mRNA of at least one HPV oncoprotein E6 and/or E7 that is grouped into severity categories wherein said groups are separated by predetermined thresholds.

XIV. A method according to any one of the preceding aspects wherein said sample is selected from the group comprising smear from a body surface, cytological smears, fine needle aspirate, body excretions, blood or serum sample, tissue biopsy, fresh frozen or formalin-fixed paraffin-embedded tissue material (FFPE), and cultured cellular material, preferably a smear from a body surface.

XV. A method of any of the preceding aspects, wherein the amount of viral and cellular mRNA, particularly the normalized amount of said viral and cellular mRNA, is introduced into a mathematical algorithm that combines said amounts and provides a score value suitable for deducing whether a HPV infection is present in said subject, and for determining the HPV type in said subject having an HPV infection, and determining whether an HPV-induced dysplasia is present in said subject having a HPV infection, and determining the severity grade of the dysplasia in a subject having a HPV-induced dysplasia, and determining whether invasive disease of cervical carcinoma is present.

XVI. A method of any of the preceding aspects, wherein the viral mRNA is selected from the group comprising the HPV types 16, 18, 31, 33, 45, 52, and 58, particularly of HPV types 16, 18, 31, 33, and 45.

XVII. A device for performing a method according to any of aspects I) to XVI) comprising:

- a probe-directed capture molecule specifically hybridizing to and allowing quantitative detection of mRNA of at least one HPV oncoprotein E6 and/or E7 by probe hybridization, wherein the HPV oncoprotein E7 and/or E6 mRNA is

selected from the group comprising the HPV types 6, 16, 18, 26, 31, 33, 35, 39, 45, 51, 52, 53, 56, 58, 59, 66, 68, 73, and/or 82, and

- a probe-directed capture molecule specifically hybridizing to and allowing quantitative detection of mRNA of at least one of the following cellular marker:

a) mRNA of at least one cellular biomarker that is preferably selected from the group comprising p16^{ink4a}, MCM2, Topo2a, Stathmin/oncoprotein 18, mKi-67, , and/or

b) mRNA of at least one tumor stem cell marker that is preferably selected from the group comprising Sox, Nanog, POU5FI/Oct3/4, ALDH1A1, and/or ALDH1L1, and/or

c) mRNA of at least one marker of the immune phenotypes that is preferably selected from the group comprising MMP7, AGR2, GDA, Keratin 7, Keratin 17, CD63, and/or p63, and/or

d) mRNA of at least one tumor marker that is preferably is selected from the group comprising Survivin/BIRC5, Telomerase, and/or p53, and

e) mRNA of a housekeeping gene,

wherein optionally, said device allows for conducting RT-qPCR or a probe hybridization assay.

XVIII. A device according to aspect XVII) comprising in addition a probe-directed capture molecule specifically hybridizing to and allowing determination of HPV E1C (syn. E1^E2), E1^E4 HPV mRNA that is selected from the group comprising the HPV types 6, 16, 18, 26, 31, 33, 35, 39, 45, 51, 52, 53, 56, 58, 59, 66, 68, 73, and 82.

XIX. A device according to aspect XVII) to XVIII) comprising in addition a probe-directed capture molecule specifically hybridizing to and allowing quantitative detection of mRNA of a housekeeping gene.

XX. A device according to any of claims XVII) to XIX) comprising further a probe-directed capture molecule specifically hybridizing to and allowing quantitative detection of mRNA of at least one HPV oncoprotein E6 and/or E7, and/or E1^E2, E1^E4, and mRNA of p16^{ink4a}, and mRNA of mKi-67, and mRNA of Stathmin, and mRNA of MCM2, and mRNA of Topo2A, and mRNA of BIRC5, and mRNA of TERT, and mRNA of ALDH1A1, and mRNA of KRT17, and mRNA of P53.

XXI. A computer readable storage medium comprising instructions to configure a processor to perform a method and/or algorithm of mathematical evaluation for the generation of a mathematical model based on the data values retrieved by a method according to any one of aspects I) to XVI) for the characterization of a sample based on evaluation of the mRNA expression profiles of the markers, wherein characterization means analysis and/or retrieving predictive information and/or profiling based on molecular mRNA expression and/or comparing results of quantitative mRNA expression analysis according to a standard.

Retrieving predicting information and/or profiling refers to the diagnosis and/or

classification of the disease stage/grade associated with a given clinical sample and, in particular, “profiling” means how the disease will develop (prediction), i.e. the expression profile points towards a progressing disease. Thus, although a dysplasia stage may look like a CIN precancerous lesion for the pathologist studying a sample histologically, the biomarkers already indicate the expression pattern of CxCa, so that a prediction or earlier detection of cervix carcinoma by the expression profile is possible, which means that it is possible to predict also whether or not a given lesion will develop into cancer. Standards can be adequately chosen by experts performing the diagnosis and/or classification of a given sample.

XXII. A computer readable storage medium comprising instructions to configure a processor to perform a method and/or algorithm of mathematical evaluation for the processing of the data of quantitative mRNA expression analysis retrieved by a method according to any one of I) to XVI), wherein in said algorithm:

a) The median of values for each marker analyzed for preferably the set of negative controls, particularly of three negative controls, is calculated, and

b) The retrieved negative-control-median value or comparable value (e.g. a threshold value in RT-qPCR) of each respective marker is subtracted from the respective quantitative value of a marker detected in said sample for the reduction of the background signal, and

c) The values obtained in step b. are divided by the value of a housekeeping gene to relativize/normalize the data measured in step a) to determine the relativized/normalized MFI.

Alternatively, it is possible to use the individual values obtained in a single measurement rather than using a median of several measurements. Further, if a given value may be regarded as generally “fixed”, such value may also be used.

XXIII. A computer readable storage medium comprising instructions to configure a processor to perform a method and/or algorithm of mathematical evaluation for the processing of the data of quantitative mRNA expression analysis retrieved by a method according to any one of claims I) to XVI), wherein dichotomization is used to determine cut-off values according to the steps in any one of aspects XXI and/or XXII) comprising grouping samples into clinical groups/clinical scores.

XXIV. A computer readable storage medium comprising instructions to configure a processor to perform a method and/or algorithm of mathematical evaluation for the processing of the data of quantitative mRNA expression analysis according to aspect XXIII), wherein cervical samples are evaluated and said clinical groups/clinical scores comprise:

- Clinical group 0, which is HPV negative and histologically without pathological findings, and
- Clinical group 1 is defined as HPV positive and histologically without pathological findings, and
- Clinical group 2 is defined as HPV positive and histologically CIN1 and

- Clinical group 3 is defined as HPV positive and histologically CIN2 and
- Clinical group 4 is defined as HPV positive and histologically CIN3 and
- Clinical group 5 is defined as HPV positive and histologically cancerous,

wherein these clinical groups are used for dichotomization into clinical thresholds.

XXV. A computer readable storage medium comprising instructions to configure a processor to perform a method and/or algorithm of mathematical evaluation for the processing of the data of quantitative mRNA expression analysis according to aspect XXIV), wherein

- clinical threshold infection is defined as separating the clinical groups 0 from the clinical groups 1-5, and
- clinical threshold CIN1+ is defined as separating the clinical groups 0-1 from the clinical groups 2-5, and
- clinical threshold CIN2+ is defined as separating the clinical groups 0-2 from the clinical groups 3-5, and
- clinical threshold CIN3+ is defined as separating the clinical groups 0-3 from the clinical groups 4-5, and
- The clinical threshold of carcinomas is defined as separating the clinical groups 0-4 from the clinical group 5.

It is noted that the detection and classification of above clinical threshold infections separating clinical groups 0 from those in groups 1 to 5 as well as clinical threshold CIN1+ from clinical groups 2 to 5 may be important to avoid overtreatment.

XXVI. A computer readable storage medium comprising instructions to configure a processor to perform a method and/or algorithm of mathematical evaluation for the processing of the data of quantitative mRNA expression analysis according to any one of aspects XXIV) or XXV), wherein the dichotomized evaluation, particularly, the determination of cut-off values, of the data retrieved by any method according to aspects I) to XVI) is used for ROC-analyses on the quantitatively determined and/or calculated relativized data selected according to the following steps:

a) all cut-off values of one marker and one clinical threshold are put in order according to their Youden-Index, which is the sum of sensitivity and specificity retrieved from ROC-analyses,

b) selecting cut-offs with the highest Youden-index,

c) evaluation of individual ROC-curves of each marker for each clinical threshold and evaluated for selected sections (e.g. cut-offs of specifically high sensitivity or specificity that can potentially add informative value when combined with other cut-offs or put differently. While one marker gives a high sensitivity for detection of a given CIN lesion, another marker may contribute a higher specificity. This can be detected in the ROC analyses by the course of the individual graphs. Combining two complementary markers will improve the overall sensitivity and specificity of the assay and lead to a better risk score. (see Figure 13-15).

XXVII. A computer readable storage medium comprising instructions to configure a processor to perform a method and/or algorithm of mathematical evaluation for the processing of the data of quantitative mRNA expression analysis based on raw or relativized data retrieved by a method according to any of I) to XVI) and XXI) to XXVI) for the calculation of predictive values of respective markers exceeding the predictive value of single marker cut-offs and to calculate a risk stratification score based on the quantities of the cellular and viral mRNA and a mathematical evaluation combining the predictive values of analyzed cellular and viral markers, particularly wherein said marker values are dichotomized into cut-off values for the stratification of the risk of subject to develop a malignant transformation and/or to deduce whether dysplasia or malignant transformation or cancer is present.

Subject matter of the present invention is also a method for diagnosing a HPV infection in a subject and identifying the HPV genotype(s) in a subject having a HPV infection and diagnosing the presence of a HPV induced dysplasia and determining the severity or grade of the dysplasia in a subject having a HPV-induced dysplasia and diagnosing invasive disease of cervical carcinoma comprising the following steps:

- quantitative determination of the expression of viral and cellular mRNA in the sample of a subject,
- wherein the viral mRNA is the mRNA of at least one HPV oncoprotein E6 and/or E7, wherein the HPV oncoprotein E7 and/or E6 mRNA is selected from the group comprising the HPV types 6, 16, 18, 26, 31, 33, 35, 39, 45, 51, 52, 53, 56, 58, 59, 66, 68, 73, and 82, and
- wherein the cellular mRNA is
    a) mRNA of at least one cellular biomarker that is preferably selected from the group comprising $p16^{ink4a}$ (syn. CDKN2A), Ki67/mKi-67, MCM2, Topo2a, Stathmin (syn. Oncoprotein 18), and
    b) mRNA of at least one tumor stem cell marker that is preferably selected from the group comprising p63, Sox2, Nanog, POU5FI/Oct3/4, ALDH1A1, ALDH1L1 and
    c) optionally mRNA of at least one marker of the squamocolumnar junction immunophenotypes that is preferably selected from the group comprising MMP7 (Matrix Metalloproteinase 7), AGR2 (anterior gradient 2), GDA (Guanin Deaminase), Keratin 7, Keratin 17, CD63, and
    d) mRNA of at least one tumor marker that is preferably selected from the group comprising BIRC5 (syn. Survivin), TERT (human telomerase-reverse transcriptase), p53 and
    e) cellular gene transcripts for internal control and normalization to the sample's cellularity: Actin-beta, Ubiquitin, HPRT, EIF4E2 (or any other housekeeping gene as mentioned below), and

deducing from the presence and/or amount of the viral mRNA and cellular mRNA whether a HPV infection is present in said subject and the HPV type in said subject having a HPV infection and deducing whether a HPV-induced dysplasia is present in said subject having a HPV infection, and/or deducing the severity or grade of the dysplasia in a subject having a HPV-induced dysplasia, and/or whether invasive disease of cervical carcinoma is present.

The present invention can be used for the detection and characterization of HPV-related lesions at any anatomical site, particularly also oropharyngeal and anogenital (anal, penile, vulval, vaginal, cervical) sites. Cervical lesions being a highly important health issue and therefore requiring systematic screening have served as the principle site for establishing the present invention. The invention contains a screening method that is based on a standard cervical smear followed by molecular evaluation via existing platforms.

As mentioned above, the present invention can be used to classify patients into the herein disclosed risk groups, e.g. in triaging methods to identify those requiring a given treatment and/or further monitoring.

In some embodiments, the invention relates to a method for treating subjects identified and/or classified using the method of the present invention. Methods for treating HPV are known to a skilled person and may be employed accordingly. When treatment is needed, abnormal cells in the cervix may be removed. Treatments might include cryosurgery, loop electrosurgical excision procedure, electrocautery, laser therapy, or applying medicated agents (i.e. cream) directly to the affected area. Early treatment in the stages designated herein may enable surprisingly beneficial recovery, thus showing the benefit of the present invention.

Panel descriptions

1) HPV E7 and E6 oncogene expression

The present invention uses quantitative analysis of expression level of the HPV oncogenes E6 and E7. These molecules play a key role in the malignant potential of HPV infection. As mentioned above, analysis of E6 and E7 oncoprotein is already used by very recently developed HPV tests. In this invention we use detection of mRNA coding for the oncoproteins or cellular proteins. The detection of mRNA has the advantage over DNA detection (e.g. by PCR) to only identify true infections and will not be false positive due to viral DNA material deposit from HPV positive partners. In addition, the crucial difference in the analysis used for the present invention lies in the quantitative aspect. While mere genotype-specific qualitative detection can only proof or rule out the presence of a viral infection, the analysis of expression quantity, as embodied in the present invention, allows a conclusion on the risk profile of said infection. Upregulation of E7 is a hallmark feature of malignant transformation in HPV infection. As shown below, E7 proves to be a strongly significant marker in the discrimination of different stages of dysplasia.

Included HPV genotypes are HPV 6, 16, 18, 26, 31, 33, 35, 39, 45, 51, 52, 53, 56, 58, 59, 66, 68, 73, and/or 82. HPV 6 represents the most common LR-HPV genotype and is responsible for appx. 90% of genital warts and is also present in many CIN1 lesions. This could be complemented by additional LR-HPV types. The HPV genotypes 16 and 18 are responsible for 70% of cervical cancers worldwide and together with HPV 31, 33, and 45 are the most carcinogenic ones. The second HR-HPV group comprises HPV 35, 39, 51, 52, 56, 58, 59, 68,

and 73 that have an intermediate carcinogenic potential. HR-HPV types 26, 53, 66, and 82 are rarely but occasionally found in cervical cancer. However, these types are more prevalent in CIN lesions. For each of the genotypes the genotype-specific oncogene E7 mRNA sequences are used as probes for detection of prevalent infection. In addition, spliced mRNA sequences (E6*I, E1C (syn. E1^E2) and E1^E4) are detected of specific genotypes to enhance detection and to be used as a biomarker of progression.

In one embodiment of the invention in said method in addition viral HPV spliced mRNA is quantitatively determined and used for the deduction of whether a HPV infection is present in said subject and identifying the HPV type in said subject having a HPV infection and deducing whether a HPV induced dysplasia is present in said subject having a HPV infection and the severity or grade of the dysplasia in a subject having a HPV induced dysplasia and, wherein the HPV spliced mRNA is selected from the group comprising the HPV types 6, 16, 18, 26, 31, 33, 35, 39, 45, 51, 52, 53, 56, 58, 59, 66, 68, 73, and 82.

In particular embodiments, the present invention relates to splice products of the most carcinogenic types (HPV16, 18, 31, 33, 45, 52, and 58), which have been identified and characterized.

Quantification of the oncogene expression, related as a ratio to the sample's cellularity by normalization to a housekeeping gene expression level, is used to evaluate the HPV positivity and the stage of the dysplasia (risk profile of said infection if an infection is present).

The following panels of groups of biomarkers are included in the diagnostic method in order to detect and describe the dysplastic lesion. Their incorporation is a novelty in the screening test and allows stage discrimination. This comprises the added value of the IVD.

2) Biomarkers associated with cellular proliferation

Markers for cellular proliferation are known to a person skilled in the art and in the present invention are defined by their established technical meaning, namely a molecular marker associated with cells undergoing proliferation, preferably cancerous proliferation. Cellular biomarker panel including $p16^{INK4a}$ (syn. p16, CDKN2A), Ki-67 (mKi-67), Stathmin-1 (STMN, Oncoprotein 18), MCM2 (minichromosome maintenance deficient 2), and Topo2A (Topoisomerase 2A). Hereinafter, the biomarker $p16^{INK4a}$ is occasionally referred to as "p16". As regulators of cellular growth these markers have been shown to be associated with cellular transformation and malignant proliferation. P16, STMN, and Ki-67 are already used in some forms of diagnostics of HPV-related transformation. However, up to now, only qualitative measurement, i.e. immunohistochemical staining (e.g. CINtec®, CINtec®PLUS, Roche), is used in diagnostic methods but no quantitative mRNA analysis is done. The present invention offers a groundbreaking novel use of these validated markers. P16 is upregulated when HR-HPV is prevalent; the strength is correlated to the cell number positive for HPV in the dysplastic field. STMN is strongly overexpressed in progressed CIN3 lesions. MCM2 is the strongest predictor of high-grade lesion found by logistic regression. Thus, the biomarkers p16, MCM2, STMN are identified as strongest predictors of a certain stage.

3) Stem Cell Markers / Cancer Stem Cell Markers

Markers for cancer stem cells are known to a person skilled in the art and in the present invention are defined by their established technical meaning, namely a molecular marker associated with a

cancer stem cell, preferably a marker that is overexpressed in cancer stem cells. The following stem cell markers have been selected and showed specificity for progressed dysplastic stages: P63 (TP63, Tumor protein p63), ALDH1A1 (aldehyde dehydrogenase 1 family, member A1), ALDH1L1 (aldehyde dehydrogenase 1 family, member L1, formyltetrahydrofolate dehydrogenase), Sox2 (SRY-box containing gene 2), Nanog (Nanog homeobox), and POU5F1 (Oct3/4, POU domain, class 5, transcription factor 1). The dedifferentiation of formerly differentiated cells and therefore the restart of undifferentiated expression patterns (i.e. stem cell expression profiles) or the uncontrolled proliferation of tissue stem cells are key characteristics of malignantly transformed cells. As shown below, some of the included stem cell markers have significant informative value for the characterization of HPV infections and dysplasia identification and were therefore included in the algorithm of the present invention. This concerns in particular but not exclusively ALDH1A1 that is regarded a ubiquitous cancer stem cell marker. It showed to be the biomarker with high value for detecting invasive disease. In addition, Sox2, Nanog, and Pouf5F1 confer pluripotency and stemness characteristics to cells, are expressed in cancer stem cells, and are markedly upregulated in invasive disease. Thus, in particular embodiments of the invention, ALDH1A1 may be included as strongest and generic cancer stem cell marker.

4) Biomarkers of the squamocolumnar junction zone

Markers for the squamocolumnar junction zone are known to a person skilled in the art and in the present invention are defined by their established technical meaning, namely a molecular marker associated with the squamocolumnar junction zone, preferably a marker that is overexpressed in the squamocolumnar junction zone. A certain well characterized cell population found at the squamocolumnar junction zone of the cervix has been the target of research based on the hypothesis that those cells might be most susceptible to HPV-induced transformation and, therefore, are regarded the cells dysplasia originates from. These cells have been characterized by the following markers that are comprised in the biomarker panel of the present invention: AGR2 (anterior gradient 2), GDA (Guanin-Deaminase), CD63 (CD63 antigen, melanoma 1 antigen, MLA1; ME491; LAMP-3; OMA81H; TSPAN30), MMP7 (Matrix Metalloproteinase 7), Krt7 (Keratin 7), and Krt17 (Keratin 17). These markers have a minor contribution for the characterization of the dysplasia. Same expression patterns have been found in other regions of transformative epithelia, like in the anal linea dentata and therefore bear potential for future use in alternative IVD use.

5) Tumor marker

Markers for tumors are known to a person skilled in the art and in the present invention are defined by their established technical meaning, namely a molecular marker associated with tumors, preferably a marker that is overexpressed in tumors, more preferably in cervical carcinoma. Invasive carcinoma is characterized by unique features, i.e. resistance to apoptosis and potential for unlimited cell division. These features are gained during the progression to invasive disease by re-expression of embryonal genes and are hallmarks of malignant disease. Included in this invention are BIRC5 (Survivin); TERT (Telomerase-reverse transcriptase); P53 (tumor protein p53, Li-Fraumeni syndrome). While BIRC5 and TERT particularly highly contribute to the detection of invasive disease, p53 is generally upregulated in HR-HPV infected cells due to the interference of E6 with p53 protein degradation. In addition, in HPV negative carcinomas p53 is generally mutated and functionally compromised, often accompanied by an overexpression.

The most informative marker for the current cervical screening application is BIRC5. Thus BIRC5 together with TERT are preferably used to identify invasive disease.

6) Cellular expression markers of cellularity

To normalize the expression and as markers for the quality of the sample the following markers were used for determination of cellularity (housekeeping markers):

Beta-Actin (ß-Actin, ACTB), HPRT1 (Hypoxanthin-Guanin-Phosphoribosyltransferase 1), UBC (Ubiquitin), EIF4E2 (syn. eukaryotic translation initiation factor 4E-like 3, 4EHP; IF4e; 4E-LP; EIF4EL3). However, other markers may also be used for normalization that have been tested for their expression. These markers include, but are not limited to:

ATP6V1A (ATPase, H+ transporting, lysosomal 70kDa, V1 subunit A, NM_001690),

B2M (Beta-2-microglobulin, NM_004048),

GAPDH (Glyceraldehyde-3-phosphate dehydrogenase, NM_002046),

GUSB (Glucuronidase, beta, NM_000181),

HMBS (Hydroxymethylbilane synthase, NM_000190),

LDHA (Lactate dehydrogenase A, NM_005566),

PGK1 (Phosphoglycerate kinase 1, NM_000291),

POLR2A (Polymerase (RNA) II (DNA directed) polypeptide A 220kDa, NM_000937) ,

PPIA (Peptidylprolyl isomerase A (cyclophilin A), NM_21130),

PPIB (Peptidylprolyl isomerase B (cyclophilin B), NM_000942),

RPL13A (Ribosomal protein L13A, NM_012423),

RPL19 (Ribosomal protein L19, NM_000981),

RPL32 (Ribosomal protein L32, NM_000994),

RPLP0 (Ribosomal protein, large, P0 (human), NM_001002),

RPS3 (Ribosomal protein S3, NM_001005),

RPS18 (Ribosomal protein S18, NM_022551),

RPS20 (Ribosomal protein S20, NM_001023),

RPS23 (Ribosomal protein S23, NM_001025),

RPS29 (Ribosomal protein S29 NM_001032),

TBP (TATA box binding protein, NM_003194),

TFRC (Transferrin receptor (p90, CD71), NM_003234),

TXN2 (Thioredoxin 2, NM_012473),

IL12A (interleukin 12A, Gene ID: 3592),

IL17B (interleukin 17B, Gene ID: 27190),

IL8 (Interleukin 8, CXCL8, Gene ID: 3576),

LDHA (lactate dehydrogenase A, Gene ID: 3939),

SERPINE1 (serpin family E member 1, Gene ID: 5054), and

TGFB1 (transforming growth factor beta 1, Gene ID: 7040).

The quantitative measuring, of a marker for cellularity (i.e. a marker expressed in a highly constant manner by human cells that, therefore, can be taken as a surrogate marker for the number of cells contained in said sample) is needed to counterbalance difference in cellularity that would lead to incomparable absolute values of measured gene expression. ACTB was shown to be the most stable marker of cellularity and therefore was used for further calculations. As described below, the ratio was calculated of absolute marker expression (unit: Mean Fluorescence Intensity, MFI) and absolute expression of the housekeeping gene ACTB (unit: Mean Fluorescence Intensity, MFI); to obtain a unit of ratio result: relative (rel.) MFI. ACTB also served as a quality check point for the evaluated sample. Samples containing very low levels of cellularity (e.g. due to insufficient cervical sampling) have eventually to be excluded and have to be resampled and retested in the setting of clinical routine diagnostics.

In a preferred embodiment of the present invention the expression of viral and cellular mRNA is quantitatively determined selected from the group comprising: mRNA of at least one HPV oncoprotein E6 and/or E7, and mRNA of HPV splice markers, and mRNA of p16^{Ink4a}, and mRNA of Stathmin, and mRNA of MCM2, and mRNA of BIRC5, and mRNA of ALDH1A1.

In one embodiment of the invention in said method mRNA of a housekeeping gene is also quantitatively determined and used for the normalization of the amount of viral and cellular mRNA in the sample of said subject and said housekeeping gene is preferably selected from the group comprising ACTB (beta-Actin), UBC, EIF4E2, HPRT1, or of any other of the above markers in particular ACTB and UBC.

Methodical Embodiments of the Present Invention

The present invention embodies a two-step procedure wherein Step One consists of the retrieval of data based on the sample of a subject (i.e. by quantitative analysis of the expression of viral and cellular mRNA) and wherein Step Two consists of the mathematical evaluation of the retrieved data with the aim to deduce 1) genotype-specific HPVdetection and if said sample is tested positive for HPV, and/or 2) risk stratification of said infection based on molecular expression profiling of cellular biomarkers with weighting of expression strength. The methods of the two steps are to be conducted as described below.

Step One: The Analytical Assay (Quantitative mRNA Analysis)

Quantitative determination of mRNA means determining the relative amount or concentration of mRNA in a sample, preferably in a multicellular sample. Quantitative determination of mRNA means also determining the normalized amount or concentration of mRNA in a sample that is preferably a multicellular sample, wherein said absolute amount or concentration maybe normalized to take into account the number of cells in the sample (cellularity). The mRNA detection is already informative on HPV positivity. This holds true for both, hybridization

techniques already used for the well-established HPV test HybridCaptureII (Cuzick et al., Br. J. Cancer 2013, 108(4):908-13) and for RT-qPCR techniques like described by Lamarcq et al. (J Mol Diagn 2002, 4:97–102).

The relative or normalized mRNA amount related to cellularity (housekeeping genes) is important for the dysplasia stage discrimination. The term relative amount and normalized amount is used synonymously throughout the application. The determined absolute or relative or normalized amount of mRNA maybe expressed as a measured value in mean fluorescence intensity (MFI) that is correlating to probe-bound mRNA from the sample, depending on the detection method used and depending on the detection label used in said method. A normalization calculation maybe for instance as follows: Value of oncogene E6 or E7 MFI divided by value of MFI of the housekeeping gene (e.g. ACTB, and/or UBC) relating it to cellularity of the sample. For convenience the resulting value maybe multiplied by a factor, e.g. 100. The latter maybe useful if working with algorithms and/or scores. The absolute and relative amount of HPV oncogene E6 and/or E7 mRNA maybe used to decide if an infection is present. The height of the value of relative amount of HPV oncogene E6 and/or E7 mRNA exceeding predetermined thresholds corresponds to the presence of a certain dysplastic stage correlating to the histologic grade of the CIN or cancer.

mRNA-based HPV -Detection

Technical cut-offs for HPV positivity

For the technical evaluation of HPV positivity of a certain HPV genotype in a sample a technical cut-off was arbitrarily chosen that is the MFI plus 3 standard deviations of the 3 background control samples consisting of the analytical reagents with addition of pure lysis buffer (containing or not containing proteinase K) instead of a clinical sample. If a sample contains HPV probes reacting with a MFI higher than the cut-off MFI value for this specific HPV genotype it is further evaluated as being genotype HPV positive.

Clinical cut-offs for HPV positivity

Since a quantitative assay is used for obtaining data from clinical samples, clinical cut-offs have been determined to define genotype-specific HPV positivity that is clinically meaningful. For the present invention, these cut-offs were determined by ROC-analyses using and comparing to genotyping results from the HPV genotyping assay Multiplexed Genotyping (MPG) (Schmitt M et al., J Clin Microbiol. 2008; 46(3):1050-9) as gold standard for statistical evaluation. As MPG offers a dichotomous result of “positive” or “negative” for HPV detection a value combining sensitivity and specificity (i.e. Youden-index) can be attributed to each HPV test result of the quantitative assay. For the QuantiGene 2.0 assay (ThermoFisher) (Figure 3) as one method for quantitative marker expression profiling in the present invention, a cut-off from HPV positivity (unit MFI) has been defined for each HPV type included in the target panel of the present invention. The cut-offs are documented in Table 1. Equivalent cut-off ct-values can be determined for RT-qPCR procedures and used analogously. Cut-off values may need to be adapted for different reagent lots.

Table 1: Cut-offs defining HPV positivity based on the quantitative assay QuantiGene™ with its sensitivity, specificity, and Youden-Index as compared to the results of Multiplex Genotyping (MPG). N = 1403.

HPV-type	n[1)]	Cut-off (MFI)	Sensitivity (%)	Specificity (%)	Youden-Index
**6**	26	35.25	38.5	99.6	0.381
**16**	448	32.25	65.8	97.1	0.629
**18**	75	54.75	78.7	95.3	0.740
**26**	1	59.50	100.0	99.9	0.999
**31**	120	31.75	81.7	95.0	0.767
**33**	61	28.25	65.6	91.0	0.566
**35**	25	33.75	84.0	99.6	0.836
**39**	56	28.75	66.1	95.2	0.613
**45**	41	64.75	61.0	93.2	0.542
**51**	86	38.75	54.7	99.3	0.540
**52**	107	27.75	59.8	96.8	0.566
**53**	62	26.75	59.7	93.0	0.527
**56**	78	29.25	60.3	98.7	0.577
**58**	45	25.25	84.4	97.1	0.815
**59**	51	26.75	64.7	92.1	0.568
**66**	60	40.75	33.3	96.5	0.298
**68**	24	23.75	79.2	78.7	0.579
**73**	30	55.50	80.0	99.6	0.796
**82**	24	31.25	66.7	99.0	0.657

MFI = mean fluorescence intensity

[1)] as compared to the results of Multiplex Genotyping (MPG), multiple infections are possible

Correlation of Oncogene Expression with Grade of Dysplasia

The scientific work that serves as the basis for the present invention shows that the height of the value (i.e. expression level) of the relative amount of HPV oncogene E6 and/or E7 mRNA corresponds to the presence of a certain dysplastic stage, i.e. correlates to the histologic grade of

the CIN or cancer. Figure 4 shows relative Mean Fluorescence Intensity (rMFI) of any strongest HPV genotype as well as exemplified for biomarkers (Fig. 1A) in relation to the stage of dysplastic progression. "Strongest" HPV genotype is defined as the one HPV genotype result with the highest net rMFI value in the case of multiple HPV infections. The same correlation is seen for HPV16 E7 as well as the splice markers HPV16E6*1 and HPV16E1^E4 (Fig. 1B) and E1C (not shown). As used herein, the discrimination between „stronger/weaker" HPV types is used to identify at least one (e.g., the strongest) of potentially multiple HPV types to put its MFI data into the formula. In other words, the "strongest HPV genotype" is the HPV genotype with strongest oncogene expression in multiple infections".

Spearman's rank correlation was used to analyze the correlation between the expression levels of the oncogenes with the stages of dysplasia. The calculated marker "strongest HPV", i.e. the one with highest rMFI value, showed the highest level of correlation (s. Table 2).

Table 2: Spearman's rank correlation coefficient (r_S) for the correlation between HPV oncogene expression (viral biomarker) and stages of dysplasia (n = 1135); exemplified for HPV16.

**Viral Oncogene Biomarker**	**r_s**
**Strongest HPV (E7)**	0,543***
**HPV 16 E7**	0,391***
**HPV 16 E6 (E6*I)**	0,385***

****p ≤ 0,001*

Table 3 shows the median of E7 (i.e. of the strongest HPV type and separately of HPV16) and HPV oncogene E6 (i.e. of HPV 16) expression levels over the different clinical stages of dysplasia (i.e. clinical groups: normal, CIN1, CIN2, CIN3, cervical cancer).

Table 3: Median of HPV oncogene expression of E7 (E7-expression of the strongest HPV type and of HPV type 16E7 and HPV16E6 (n = 1135).

	**Clinical Groups**				
**Viral Oncogene Biomarker (Median of rMFI)**	**Normal**	**CIN1**	**CIN2**	**CIN3**	**Cervical carcinoma**
**Strongest HPV E7**	0.67	1.05	2.02	2.27	4.015
**HPV 16 E7**	1.13	0.61	1.63	2.22	3.23
	0.98	0.09	1.65	2.03	3.28

HPV 16 E6*1

The mathematical median was used for the proof of principle that HPV oncogene expression correlates with the clinical stages of dysplasia. Since the marker expression levels in said samples over the clinical stages cannot be described by a curve of normal distributions, more complex statistical analyses had to be performed to allow precise expression profiling of the different clinical stages of dysplasia. The statistical methods are explained below.

For statistical evaluation, the expression of the oncogene E7 was used for correlation wherein in infections with multiple HPV genotypes the genotype that showed the highest level of oncogene E7 expression was chosen for calculation (s. above establishment of the calculated marker "strongest HPV"). Fig. 5-7 show the ROC curves of the viral markers for the three most relevant clinical thresholds.

QuantiGene principle

The quantitative determination of the expression of viral and cellular mRNA in the sample of a subject may be conducted by several methods. One of them uses the probe hybridization. The QuantiGene 2.0 method uses a solid phase-bound probe-directed capture of a specific target mRNA. This "capture probe" formed complex is then detected and quantified by a second probe set "detector probe" binding specifically to the captured mRNA and being detected by the branched DNA technology with corresponding fluorescent label that can be quantified and is directly proportional to the bound mRNA.

Branched DNA (bDNA) technology constitutes a method of signal amplification (as opposed to target amplification). bDNA molecules bind specifically to a determined target sequence. This sequence is introduced in the detector probe sequence. bDNA by hybridization to complementary sequences builds up a multimeric structure that enables a multiplied reporter fluorochrome binding thus introducing a reporter label. The signal of a given target is enhanced (rather than a target being amplified) in order to create a signal that is strong enough to be detected. Since bDNA multimer formation is standardized the technology thereby produces a signal that is proportional to the absolute amount of initially captured target molecules and allows quantitative analysis of a given target.

In one preferred embodiment of the present invention the quantitative determination of mRNA of at least one HPV oncoprotein and/ or HPV spliced mRNA and/or mRNA of a housekeeping gene and/or mRNA of a cellular biomarker and / or mRNA of a tumor stem cell marker and/or mRNA of a marker of the immune phenotype and/or mRNA of a tumor marker is conducted in a single step by using multiplexing techniques (e.g. Luminex-based analysis).

Preferred Methodological Embodiment: Luminex Assay

A method of quantitatively determining viral and cellular mRNA maybe conducted as follows: Cellular material from a clinical sample (or an experimental sample) is lysed to liberate the cellular constituents (e.g., mRNA) and to stabilize, or by isolating mRNA from a sample. This mRNA containing material is incubated with surface-bound oligonucleotides, e.g. beads that capture the specific mRNA by hybridization over a certain time interval and extract it from the lysate. The bound mRNA is detected by binding of label extender oligonucleotides and the

specificity is enhanced by blocking potentially interfering target sequences by blocking oligonucleotides. Label extender oligonucleotides hybridize to a branched DNA signal amplification system that confers a fluorescent signal to the complex that is proportional to the captured target sequences on the surface-support. The amount of fluorescence signal can be measured by a suitable device and quantified, since the signal is directly proportional to the amount of mRNA. Such devices may be commercially available, e.g. by Luminex (https://www.luminexcorp.com/instruments/). The extent of the fluorescence is expressed as mean fluorescence intensity (MFI) of several parallel measurements. The different target sequences capture-surfaces are multiplexed in a single assay format.

The device used for the methods of the present invention maybe equipped to excite fluorochromes and measure emitted fluorescence in a quantifying way. This can be a flow-cytometric device passing microspheres in a stream by a laser beam and photomultiplier, or a device taking images of immobilized probe-target-label complexes. The device maybe equipped with a computer and software calculating the MFI of a certain class if probe-target-label complexes. Values are given out in a mathematical table (e.g., Excel spread sheet).

Thus, in one embodiment of the present invention the expression of viral and cellular mRNA is quantitatively determined, e.g., using RT-qPCR, comprising the following steps:

- providing a device or a set of reagents comprising:
    - o a probe-directed capture molecule for specifically hybridizing to and allowing determination of mRNA of at least one HPV oncoprotein E6 and/or E7 by probe hybridization, wherein the HPV oncoprotein E7 and/or E6 mRNA is selected from the group comprising the HPV types 6, 16, 18, 26, 31, 33, 35, 39, 45, 51, 52, 53, 56, 58, 59, 66, 68, 73, and 82, and
    - o a probe-directed capture molecule for specifically hybridizing to and allowing determination of mRNA of at least one of the following cellular marker:
        a) mRNA of at least one cellular biomarker that is preferably selected from the group comprising p16^{ink4a}, MCM2, Topo2a, Stathmin/oncoprotein 18, mKi-67, and
        b) mRNA of at least one tumor stem cell marker that is preferably selected from the group comprising Sox, Nanog, POU5FI/Oct3/4, ALDH1A1, and ALDH1L1, and
        c) mRNA of at least one marker of the immune phenotypes that is preferably selected from the group comprising MMP7, AGR2, GDA, Keratin 7, Keratin 17, CD63, and p63, and
        d) mRNA of at least one tumor marker that preferably is selected from the group comprising BIRC5/Survivin, Telomerase (TERT), and, p53 and/or
        e) mRNA of at least one housekeeping cellular marker (e.g., Actin-ß),

- bringing said device into contact with a sample of said subject allowing hybridization of said above-mentioned probe-directed capture molecules with the mRNA present in said sample of said subject,
- determining quantitatively the mRNA of said at least one HPV oncoprotein E6 and/or E7 and determining quantitatively the mRNA of the above-identified panel marker according to a); b); c); d), and e).

The provided device or set of reagents may comprise in addition to the above cited viral mRNA or alternatively to the above cited of mRNA of at least one HPV oncoprotein E6 and/or E7, a probe-directed capture molecule for specifically hybridizing to and allowing determination of HPV splice marker mRNA that is selected from the group comprising the HPV types 6, 16, 18, 26, 31, 33, 35, 39, 45, 51, 52, 53, 56, 58, 59, 66, 68, 73, and 82.

The provided device may comprise, in addition to the above-cited mRNA, a probe-directed capture molecule for specifically hybridizing to and allowing determination of mRNA of a housekeeping gene as defined above.

In a preferred embodiment, the expression of viral and cellular mRNA is quantitatively determined comprising the following steps:

- providing a device or a set of reagents comprising:
- a probe-directed capture molecule for specifically hybridizing to and allowing determination of mRNA of at least one HPV oncoprotein E6 and/or E7, and
- a probe-directed capture molecule for specifically hybridizing to and allowing determination of mRNA of at least one HPV oncoprotein splice marker, and
- a probe-directed capture molecule for specifically hybridizing to and allowing determination of mRNA of $p16^{ink4a}$, and
- a probe-directed capture molecule for specifically hybridizing to and allowing determination of mRNA of Stathmin, and
- a probe-directed capture molecule for specifically hybridizing to and allowing determination of mRNA of MCM2, and
- a probe-directed capture molecule for specifically hybridizing to and allowing determination of mRNA of ALDH1A1, and
- a probe-directed capture molecule for specifically hybridizing to and allowing determination of mRNA of BIRC5, and
- a probe-directed capture molecule for specifically hybridizing to and allowing determination of mRNA of TERT.

In a preferred embodiment, the quantitative determination of the expression of viral and cellular mRNA in the sample of a subject is performed as single step method. The different target sequences capture-surfaces maybe multiplexed in a single assay format. This means more than one target can be measured at the same time. In our assay, all targets are measured at the same time.

According to the present invention said sample is selected from the group comprising smear from a body surface, fine needle aspirate, body excretions, blood or serum sample, tissue biopsy, fresh frozen or formalin-fixed paraffin-embedded tissue material (FFPE) and cultured cellular material, preferably smear sample, fresh biopsy, or FFPE from a body surface to be tested for HPV or dysplasia.

In one embodiment of the present invention the amount of viral and cellular mRNA, preferably the normalized amount of said viral and cellular mRNA, is introduced into an mathematical algorithm that combines mathematically said amounts and gives as an output a score value which is used to deduce whether a HPV infection is present in said subject and the HPV type in said subject having a HPV infection and deducing whether a HPV-induced dysplasia is present in said subject having a HPV infection and deducing the severity or grade of the dysplasia in a subject having a HPV-induced dysplasia and whether invasive disease of cervical carcinoma is present.

Examples for algorithms (following below) (Step 2).

In one preferred embodiment of the present invention, establishment of this risk stratification score is calculated according to the following steps wherein

- each clinical threshold is to be examined separately in order to allow dichotomization
- markers that show a significant AUC of ROC-analysis are used for univariate logistic regression
- the markers that prove to have the highest OR in defining a clinical threshold retrieved from univariate logistic regression are included in a multivariate logistic regression
- forward step multivariate logistic regression is performed
- The regression function of the multivariate binary logistic regression as shown in formula 1 is used as base for the establishment of the risk score.

Formula 1: Model of the regression function of the multivariate binary logistic regression calculating the probability (p) of the dependent variable y as a function of the independent variables x1 to xn (i.e. mRNA-levels of single markers).

$$p(y) = \frac{1}{1 + e^{-(a + b_1 \cdot x_1 + b_2 \cdot x_2 + \dots + b_n \cdot x_n)}}$$

a = **mathematical constant** *of the multivariate regression*

b = **standardized coefficient b** *of each variable x*

x = value of each independent variable

In further embodiments of the above method of generating a risk stratification score

i. values for a and b are retrieved from multivariate logistic regression,

ii. x1 to xn being defined 1 if a predefined cut-off is being exceeded by the according measured value of a given sample, and

iii. x1 to xn being defined 0 if a predefined cut-off is not being exceeded by the according measured value of a given sample.

In further embodiments of the present invention the following risk stratification scores are used for molecular expression profiling, i.e. for evaluating on the marker mRNA expression profile the risk of malignant transformation within a present HPV infection and/or assessing the probability of the presence of dysplasia or cancer and/or evaluating the stage of dysplasia

Formula 2:

iv) <u>threshold CIN2+</u> (clinical thresholds are used according to claim 15): based on evaluation according to claim 1-10 of the markers strongest HPVE7, HPV16 E6, HPV16 E1^E4, $p16^{ink4a}$ and Stathmin according to the following formula

$$\mathbf{p(CIN2+)} = \frac{\mathbf{1}}{\mathbf{1} + \mathbf{e}^{-(-1.430 + 1.364 \cdot x_1 + 0.718 \cdot x_2 + 0.612 \cdot x_3 + 0.928 \cdot x_4 + 0.413 \cdot x_5)}}$$

x_1 = 0 or 1, if strongest HPV < or > 1.34 rel.MFI
x_2 = 0 or 1, if HPV 16 E6*I < or > 1.17 rel.MFI
x_3 = 0 or 1, if HPV 16 E1^E4 < or > 1.00 rel.MFI
x_4 = 0 or 1, if p16 < or > 6.47 rel.MFI
x_5 = 0 or 1, if stathmin < or > 17.33 rel.MFI

Formula 3:

v) <u>threshold CIN3+:</u> based on evaluation according to the above methods of the markers: HPV 16 E6, HPV 16 E1^E4, strongest HPV, p16, MCM2 according to the following formula

$$\mathbf{p(CIN3+)} = \frac{\mathbf{1}}{\mathbf{1} + \mathbf{e}^{-(-2.396 + 0.697 \cdot x_1 + 0.736 \cdot x_2 + 0.794 \cdot x_3 + 1.097 \cdot x_4 + 0.582 \cdot x_5)}}$$

x_1 = 0 or 1, if HPV 16 E6*I < or > 1.34 rMFI
x_2 = 0 or 1, if HPV 16 E1^E4 < or > 1.05 rMFI
x_3 = 0 or 1, if strongest HPV < or > 1.40 rMFI
x_4 = 0 or 1, if p16 < or > 11.10 rMFI
x_5 = 0 or 1, if MCM2 < or > 5.19 rMFI

Formula 4:

vi) threshold to carcinoma: based on evaluation according to the above methods of markers: strongest HPV, BIRC5, ALDH1A1, TERT, and MCM2 according to the following formula

$$p(CxCa) = \frac{1}{1 + e^{-(-6.563 + 1.391 \cdot x_1 + 1.227 \cdot x_2 + 1.444 \cdot x_3 + 1.166 \cdot x_4 + 1.142 \cdot x_5)}}$$

x_1 = 0 or 1, if MCM2 < or > 7.96 rMFI
x_2 = 0 or 1, if BIRC5 < or > 1.52 rMFI
x_3 = 0 or 1, if ALDH1A1 < or > 2.99 rMFI
x_4 = 0 or 1, if TERT < or > 0.49 rMFI
x_5 = 0 or 1, if strongest *HPV < or > 13.43 rMFI*

In a further embodiment, the present invention relates to a computer readable storage medium comprising instructions to configure a processor to perform a method and/or algorithm of mathematical evaluation for the processing of the data of quantitative mRNA expression analysis, a method and/or algorithm of mathematical evaluation that allows the definition of at least one respective most valuable cut-off of the risk score that is used as a dichotomous test decision whether a clinical status as defined by a clinical threshold is reached or not as evaluated by means of quantitative mRNA analysis performed via any method according to the present invention.

In another embodiment of the present invention, the risk score cut-offs are calculated using ROC-analyses wherein

a. the values of the risk score are evaluated for their sensitivity and specificity based on the clinical score of the examined clinical threshold and

b. the most significant cut-offs are determined via the Youden-Index.

Methodological Alternatives

Alternative methods to the above described procedure allowing mRNA quantification are RT-qPCR, Nanostring, and Affymetrics chip analyses (Scerri et al., Methods. 2018: S1046-2023(18)30168-3). They can in principle also quantify the expression level of genes by measuring and evaluating specific mRNA frequency in a sample. From the quantification cut-off levels can be deduced that are informative for dysplasia stages. For example, by RT-qPCR the relative concentration of amplified cDNA amplicon of a certain mRNA species can be measured and the relative expression of a target mRNA versus a housekeeping mRNA calculated. For this method RNA has to be isolated from a sample and subjected to cDNA synthesis and then qPCR amplification. This qPCR can be multiplexed for up to 5 targets. RT-qPCR is evaluated by detecting the ct-value (cycle threshold), i.e. the cycle number where the measured read-out

fluorescence exceeds for the first time the background fluorescence of a sample. From this value a relation to a housekeeping gene expression can be calculated and thus a relative expression level determined.

By Nanostring individual localized mRNA molecules are identified by color-fluorochrome-barcoded probes and optically counted for quantification of the target mRNA. By Affymetrics chip hybridization relative brightness of a target mRNA hybridized to a spotted probe can be used to quantify expression strength.

Step Two: Mathematical Evaluation (Establishment of the Analytical Algorithm)

In the present invention, the raw data (unit: Mean Fluorescence Intensity, MFI and relative MFI, rMFI, ct values or other read out values of mRNA quantitation methods) is used for the evaluation of HPV positivity and strength of HPV oncogene and cellular biomarker expression. The strength of expression correlates to stage of dysplasia. By combining expression values of several biomarkers an improved prediction for the presence of a dysplastic stage can be obtained.

For the quantitative evaluation of biomarker expression, the raw data is edited according to the following steps:

1. Subtraction of the signal background (arithmetic average of the negative controls that have to be included in each assay) of all markers including housekeeping genes
2. Normalization (devision by) to the expression signal of a housekeeping gene (here preferable used Actin-β).
3. Multiplication by factor 100 (or else what is suitable to facilitate data handling)

By implementing step 1-3 the unit of MFI is changed to relative MFI (rMFI).

4. rMFI < 0 are to be set to 0.

For the proof of principle of the present invention, clinical samples (cervical smears) were used and grouped into clinical groups according to the histological diagnosis. Groups were defined as shown in Table 4. “HPV negative” was defined according to a negative test result of the comparative measurement method by MPG.

Table 4: Definition of Clinical Score

Clinical Score	Definition
**0**	HPV negative, histologically without pathological findings
**1**	HPV positive, histologically without pathological findings
**2**	HPV positive, histologically CIN1
**3**	HPV positive, histologically CIN2
**4**	HPV positive, histologically CIN3
**5**	HPV positive, histologically Carcinoma

CIN = cervical intraepithelial neoplasia

In one preferred embodiment of the present invention, “Strongest HPV” is to be introduced as a calculated marker based on a sample being HPV positive and having an HPV infection with multiple genotypes. The HPV genotype showing the highest level of E7 expression (rMFI) is then to be defined as “Strongest HPV”. In HPV-negative samples, “Strongest HPV” is to be set to 0. Statistical evaluation has been performed with the software SPSS Statistics Version 23 for Windows (IBM, Armonk, USA) und Excel 2010 (Microsoft Corp., Redmond, USA).

Rank Correlation

In the present invention, the Kendall rank correlation coefficient was used to evaluate the degree of statistical correlation between marker expression (rMFI) and stage of dysplasia (i.e. clinical score). According to the Kendall’s tau coefficient significantly correlating markers were selected (threshold for positive selection is a significance level of correlation of $p < 0.001$). The statistical correlation amongst markers was assessed with the Spearman's rank correlation coefficient. Threshold for positive selection was a significance level of correlation of $p < 0.001$.

Selecting Marker Expression Cut-offs: ROC-Analyses

In order to define thresholds of marker expression that differentiate stages of dysplasia with the highest possible accuracy (highest possible sensitivity and specificity) Receiver Operating Characteristics (ROC) – analyses were performed. ROC-analyses allowed the reduction of the molecular test results (unit: rMFI) into a binary test result. Each clinical threshold (e.g. ≤CIN3 vs. CIN3 +) had to be evaluated separately for each marker. The panel of markers had to be pre-selected according to its informative value via Kendall’ tau correlation coefficient. For each marker and each relevant clinical threshold, a selection of most valuable cut-offs was selected by means of ROC-analyses. These most valuable cut-offs were selected according to the following principles:

1. All possible cut-offs for one marker and one clinical threshold were put in order according to their Youden-index (i.e. the sum of sensitivity and specificity) retrieved from ROC-analyses. Cut-offs with the highest Youden-index were selected.

2. The individual ROC-curve of each marker for each clinical threshold was evaluated for specifically valuable sections (e.g. cut-offs of specifically high sensitivity or specificity that can potentially add informative value when combined with other cut-offs). Therefore, alternative marker combinations would also be possible depending on alternative patients, e.g. pregnant women, HIV positive subjects, different tissue origin of smear sample.

The overall informative value of one marker or of marker combinations for one clinical threshold can be evaluated by means of the Area under Curve (AUC) of the ROC-analysis.

Combining Markers: Logistic Regression

The selection of cut-offs of all markers that have been proven to be significant in defining one clinical threshold were then combined into one model by the statistical means of logistic regression. In the present invention, logistic regression allows the selection of marker cut-offs that mutually add value and thereby allow the characterization of a given clinical threshold with a greater accuracy than single marker cut-offs. Forward-step regression has been used.

Calculating a Risk Score

The present invention allows a binary final test result that defines a given sample based on its molecular expression profile as above or below a given clinical threshold and/or defines a given sample into two groups according to the threshold “high risk of relevant dysplasia vs. low risk of relevant dysplasia”. In order to allow a binary final test result, the combination of marker cut-offs – as selected via multivariate analysis of the logistic regression were combined into one risk score. The multivariate binary logistic regression provides the mathematical constant a and the standardized coefficient b that are to be used in the establishment of the regression function (see formula 1). The regression formula allows determination of the probability of y (defined as disease / classification as “high risk of certain disease stage”) as a function of the independent variables x_1 to x_n (defined as marker expression levels, unit rMFI) multiplied. The variables x_1 to x_n (i.e. the single and selected marker expression levels) are each multiplied by the standardized coefficient b and thereby weighted according to their informative power in predicting the probability of y (i.e. disease / classification as “high-risk of certain disease stage”).

Generic Formula 1: Model of the regression function of the multivariate binary logistic regression calculating the probability of the dependent variable y as a function of the independent variables x_1 to x_n.

$$p(y) = \frac{1}{1 + e^{-(a + b_1 \cdot x_1 + b_2 \cdot x_2 + a + b_n \cdot x_n)}}$$

a = mathematical constant *of the multivariate regression*

b = standardized coefficient b *of each variable x*

x = value of each independent variable

As a last step, cut-offs of the height of probability of y can be evaluated defining categories of clinical follow-up algorithms and or clinical treatment. These cut-offs are to be found via ROC-analyses that calculate the sensitivity and specificity of a given value of p(y) in describing a clinical threshold, such as ≤CIN3/CIN3+. Preferred calculations based on preferred biomarkers are given as an example:

Formula 2:

a. threshold CIN2+ (clinical thresholds are used according to claim 15): based on evaluation according to claim 1-10 of the markers strongest HPVE7, HPV16 E6, HPV16 E1^E4, p16 and Stathmin according to the following formula

$$p(\mathrm{CIN2}+) = \frac{1}{1 + e^{-(-0.430 + 1.364 \cdot x_1 + 0.718 \cdot x_2 + 0.612 \cdot x_3 + 0.928 \cdot x_4 + 0.413 \cdot x_5)}}$$

x_1 = 0 or 1, if strongest HPVE7 < or > 1.34 rMFI

*x_2 = 0 or 1, if HPV16 E6*I < or > 1.17 rMFI*
x_3 = 0 or 1, if HPV16 E1^E4 < or > 1.00 rMFI
x_4 = 0 or 1, if p16 < or > 6.47 rMFI
x_5 = 0 or 1, if stathmin < or > 17.33 rMFI

Formula 3:

b. threshold CIN3+: based on evaluation according to claim 1-10 of the markers strongest HPVE7, HPV16 E6, HPV16 E1^E4, p16, MCM2 according to the following formula

$$p(\mathrm{CIN3}+) = \frac{1}{1 + e^{-(-2{,}396 + 0{,}697 \cdot x_1 + 0.736 \cdot x_2 + 0.794 \cdot x_3 + 1.097 \cdot x_4 + 0.582 \cdot x_5)}}$$

*x_1 = 0 or 1, if HPV 16 E6*I < or > 1.34 rMFI*
x_2 = 0 or 1, if HPV 16 E1^E4 < or > 1.05 rMFI
x_3 = 0 or 1, if strongest HPVE7 < or > 1.40 rMFI
x_4 = 0 or 1, if p16 < or > 11.10 rMFI
x_5 = 0 or 1, if MCM2 < or > 5.19 rMFI

Formula 4:

c. threshold to carcinoma: based on evaluation according to claim 1-10 of the markers strongest HPVE7, BIRC5, ALDH1A1, TERT, and MCM2 according to the following formula

$$p(\mathrm{CxCa}) = \frac{1}{1 + e^{-(-6.563 + 1.391 \cdot x_1 + 1.227 \cdot x_2 + 1.444 \cdot x_3 + 1166 \cdot x_4 + 1.142 \cdot x_5)}}$$

x_1 = 0 or 1, if MCM2 < or > 7.96 rMFI
x_2 = 0 or 1, if BIRC5 < or > 1.52 rMFI
x_3 = 0 or 1, if ALDH1A1 < or > 2.99 rMFI
x_4 = 0 or 1, if TERT < or > 0.49 rMFI
x_5 = 0 or 1, if strongest HPVE7 < or > 13.43 rMFI

Example of severity determination of HPV infection and stage of dysplasia

The following marker and marker panels and the use of certain threshold values are examples how to determine the severity of a dysplasia. According to the invention a normalized amount of

HPV mRNA of at least one HPV oncoprotein E6 and/or E7 is correlated with the presence of HPV infection or severity or grade of dysplasia:

- Wherein said subject has a HPV infection if said normalized amount of HPV mRNA of at least one HPV oncoprotein E6 and/or E7 is above a threshold value of the negative control, and/or
- Above a Threshold 1 that is the mean fluorescence intensity of mRNA level found in CIN1, and
- Above a Threshold 2: that is the mean fluorescence intensity of mRNA level found in CIN2, and
- Above a Threshold 3: that is the mean fluorescence intensity of mRNA level found in CIN3, and
- Above a Threshold 4: that is the mean fluorescence intensity of mRNA level found in cancer

In one embodiment of the invention a normalized amount of HPV mRNA of at least one HPV oncoprotein E6 and/or E7 is correlated with the presence of HPV infection or severity or grade of dysplasia, and wherein dysplasia is present if said normalized amount of HPV mRNA of at least one HPV oncoprotein E6 and/or E7 is above a predetermined threshold in said subject.

In embodiments of the present invention, the severity of dysplasia is determined by the quantified normalized amount of HPV mRNA of at least one HPV oncoprotein E6 and/or E7 that is grouped into severity categories wherein said groups are separated by predetermined thresholds.

In another embodiment the detection of HPV infection is conducted in conjunction with a combined biomarker expression above a certain pre-defined threshold.

- Infection defining markers: detectable expression of HPV oncoprotein E6 or E7 and/ or HPV spliced mRNA and of $p16^{ink4a}$ above baseline, while baseline expression of other markers in the panel that would be higher expressed in more progressed stages (e.g. Stathmin in CIN3, BIRC5 in cancer). Baseline is the mean expression level in non-HPV infected healthy tissue;
- CIN1 defining markers: detectable and increased (as compared to infection) expression of HPV oncoprotein E6 or E7 and/ or HPV spliced mRNA and of increased expression (baseline and infection) of $p16^{ink4a}$, while baseline expression of other markers in the panel:
- CIN2 defining markers: detectable and further increased (as compared to infection and CIN1) expression of HPV oncoprotein E6 or E7 and/ or HPV spliced mRNA and of $p16^{ink4a}$, while baseline expression of other markers in the panel;
- CIN3 defining markers: detectable and further increased (as compared to infection and CIN1 and CIN2) expression of HPV oncoprotein E6 or E7 and/ or HPV spliced mRNA and of $p16^{ink4a}$, and increased expression of Stathmin, MCM2, or Topo2a, and low level increase of cancer stem marker ALDH1A1 and Sox, while baseline expression of other markers (Tumor and cancer stem cell) in the panel;

- Cervical Carcinoma defining markers: detectable and further increased (as compared to infection and CIN1 and CIN2 and CIN3) expression of HPV oncoprotein E6 or E7 and/ or HPV spliced mRNA and of p16^{ink4a}, and increased expression of Stathmin, MCM2, Topo2a, and high-level increase of cancer stem cell marker ALDH1A1 and Sox, and Nanog and Pou5FI and tumor markers Survivin/BIRC5 and TERT and p53.

The detection of mRNA for the HPV oncogene E6 and E7 and HPV spliced mRNA and cellular biomarker and cellular housekeeping genes is achieved by a method using specific oligonucleotides as capture and detector probe. The oligonucleotides are commercially available from ThermoFisher from the QuantiGene 2.0 RNA Probe Set Catalog: http://cdn.panomics.com/products/gene-expression/qg2psc.

As used herein, a threshold that has been predefined according to the algorithm defined in the presented invention ROC-analyses are performed using the raw data or relative values retrieved by any method according to any one of the preceding embodiments using genotyping results from a MPG method or any other established method as gold standard for the statistical evaluation. ROC-analyses can be used for dichotomization of said data analyzing preferably each HPV genotype separately defining HPV genotype-specific cut-off values that allow the most precise definition of HPV positivity as predefined by the result of the gold standard method.

FIGURES

The present invention is further described by reference to the following non-limiting figures. The figures describe non-limiting and practical embodiments, presented for further illustration of the invention.

Figure 1: Progression of cervical dysplasia to invasive cancer and use of biomarker expression to identify HPV infection and stage of dysplastic alterations ("molecular histology"). Cervical epithelium shows characteristic cellular and histological alterations during progression from normal, HPV infected, premalignant to malignant stages. Regression and progression is possible until development of invasive cancer. The long term interval is allowing for screening and treatment. Identification of dysplastic stage can be done by quantification of biomarker expression (here mRNA quantification). A prerequisite for cervical disease development is infection by HR-HPV types. The expression strength of the strongest HPV type detected is an informative parameter on lesion progression. In addition, characteristic biomarkers become up regulated during certain steps of progression like Stathmin in CIN3 and tumor markers BIRC5 and TERT in invasive cervical cancer. Expression has to be normalized to the cellularity of a given sample what is achieved by quantification of housekeeping gene expression.

Figure 2: Schematic representation of the use of stage specific biomarkers and strength of expression for dysplasia detection. The relation of the biomarker expression to the expression of the housekeeper ACTB allows for comparison of different smears. ACTB is expressed continuously in different dysplastic stages. Expression of the different biomarkers is induced differently depending of the progressive stage of the dysplasia. Therefore, these biomarkers can be used differentially in the respective risk score formulas for the individual dysplastic stages. Some of the markers are expressed dependently and can replace each other.

Figure 3: QuantiGene 2.0 assay platform. Multiplexed detection and quantification of mRNA by QuantiGene 2.0 assay platform. Colour-coded Luminex beads are used as scaffold for capture

probes for specific mRNA sequences. mRNA is captured by hybridization and detected by detector probes that in turn bind label extender and branched DNA (bDNA) giving rise to a signal that is directly proportional to the bound mRNA. (modified from: Scerri et al., Methods. 2018: S1046-2023(18)30168-3).

Figure 4: Median of HPV oncogene and biomarker expression for different stages of cervical intraepithelial neoplasia (CIN) and cervical carcinoma. A) hrHPV-positive women (n = 954) B) HPV16-positive women (n = 328). For better mathematical evaluation and presentation the natural logarithm (Ln) of the relative mean fluorescence intensity (rMFI) is used. The median relative expression (as Ln for compressed presentation) shows how different markers increase with progressive cervical disease. While some markers show a continuous increase (like „strongest HPV“ and “p16”) others show increased expression in certain stages (like Stathmin in CIN3 and BIRC5 in cervical cancer). Most markers show enhanced expression as of CIN3 as the biological hallmark of enhanced proliferation and malignant transformation.

Figure 5: ROC-analysis of the viral markers HPV16 E6, E7, and strongest HPV (selected from all 18 HR-HPV) for the clinical threshold CIN2+ (n = 1135).

Figure 6: ROC-analysis of the viral markers HPV16 E6, E7, and strongest HPV for the clinical threshold CIN3+ (n = 1135).

Figure 7: ROC-analysis of the viral markers HPV16 E6, E7, and strongest HPV for the clinical threshold Cervical Cancer (n = 1135).

Figure 8: The ROC curves show a positive contribution (area > 0.5, represented by the diagonal reference line) for most markers but with different area for the discrimination of normal versus HPV-infected epithelium.

Figure 9: The ROC curves show a positive contribution (area > 0.5, represented by the diagonal reference line) for most markers but with different area for the discrimination of non-dysplastic versus dysplastic epithelium.

Figure 10: The ROC curves show a positive contribution (area > 0.5, represented by the diagonal reference line) for most markers but with different area for the discrimination of ≤CIN1 versus CIN2+ epithelium.

Figure 11: The ROC curves show a positive contribution (area > 0.5, represented by the diagonal reference line) for most biomarkers but with different area. The combination of strongestHPV.1 with $p16^{ink4a}$ and Stathmin and MCM2 will increase the AUC as compared to the individual biomarkers because these peak in different regions of the curves.

Figure 12: The ROC curves show a positive contribution (area > 0.5, represented by the diagonal reference line) for most biomarkers but with different area. All biomarkers have higher AUC than in the analyses before proving their stronger expression in invasive disease. Proliferation associated (MCM2, Topo2a, MKi67) and tumor markers (BIRC5, TERT) and cancer stem cell markers (ALDH1A1) gain importance.

Figure 13: The ROC curves for discrimination of ≤CIN1 versus CIN2+show a higher area AUC of the biomarker combinations as compared to the strongestHPV.1 alone. While the addition of p16 (risk345scorekonHPV4) adds to the AUC the further addition of MCM2 (risk345scorekonHPV4a) does not add significantly at this dysplasia threshold.

Figure 14: At this threshold for discrimination of ≤CIN2 versus CIN3+ “p16^{ink4a}” has a higher AUC than „StrongestHPV.1“as most HPV positive cases are more evenly distributed between the two groups compared, while strength of p16 expression is markedly enhanced in dysplasia of higher grade.

The addition of proliferation-associated markers (STMN, MCM2) and HPV splice markers further enhances the AUC of “risk45scoreHPV” significantly.

Figure 15: While “StrongestHPV detection” for discrimination of ≤ CIN3 versus CxCa (N.B. here differences in quantification of expression) achieves already a high AUC the proliferation-associated biomarker MCM2 has an increased AUC and the biomarker combination of both “risk5scoreHPV” has a significantly higher AUC.

Figure 16: A) The values of the QuantiGene, relativized to the reference gene ACTB and logarithmised to base two, are plotted against the ΔCq (quantification cycle)-values of the RT-qPCR. Shown is the data for the following marker: STMN1, MCM2, KI67, BIRC5, ALDH1A1, HPV16-E7 and HPV18-E7. The data for each marker is divided into normal histology (grey circle), CIN I (dark grey rhombus), CIN II (dark grey circle), CIN II (black triangle) and CxCa (grey square). B) The table shows the calculated correlation coefficient from the data shown in A for each marker (CIN = cervical intraepithelial neoplasia; CxCa = cervical carcinoma).

Figure 17: Correlation of QuantiGene versus RT-qPCR results in corresponding samples. The values of the QuantiGene, normalized to ACTB, are plotted against the Δct values of the RT-qPCR, also normalized to ACTB. The data of each marker are divided into normal histology and HPV negative, normal histology and HPV positive, CIN I, CIN II, CIN III and CxCa. n=74

Figure 18: Biomarker expression correlated to dysplasia stages. Median Biomarker expression measured by RT-qPCR (Δct values) in different stages of the cervical dysplasia (CIN) and CxCa.

Figure 19: ROC curves of the analysed biomarkers for the CIN2+ threshold.

Figure 20: ROC curves of the analyzed biomarkers for the CIN3+ threshold.

Figure 21: ROC curves of the analyzed biomarkers for the CxCa threshold.

EXAMPLES

The present invention is further described by reference to the following non-limiting examples. The examples describe non-limiting and practical embodiments, presented for further illustration of the invention.

Contribution of individual biomarkers to risk score for different dysplastic stages

In order to evaluate the value of each biomarker for the identification of a certain stage of dysplasia a ROC analysis was done with data of well characterized clinical samples. The AUC was taken as the measure of quality of each biomarker to discriminate and to identify the different stages under comparison (Figs. 8-12).

Normal epithelium vs HPV-infected epithelium: Group 0 vs. 1-5 (analysis score 15)

HPV and biomarker contribution for the differentiation of the two groups 0 vs 1-5 is presented in Fig. 8.

Table 5:

Case Processing Summary

Score15[a]	Valid N (listwise)
Positive[b]	954
Negative	181
Missing	303

Larger values of the test result variable(s) indicate stronger evidence for a positive actual state.

a. The test result variable(s): Strongest HPV.1 has at least one tie between the positive actual state group and the negative actual state group.

b. The positive actual state is 1-5.

Table 6:

Area Under the Curve for group 0 vs. 1-5

Test Result Variable(s)	Area	Asymptotic Sig.[b]	Asymptotic 95% Confidence Interval	
			Lower Bound	Upper Bound
StrongestHPV.1	.943	.000	.930	.956
p16	.712	.000	.672	.753
STMN1	.601	.000	.556	.646
MCM2	.622	.000	.579	.665
Topo2A	.615	.000	.571	.660
MKi67	.622	.000	.579	.665
p63	.580	.001	.533	.627
ALDH1A1	.524	.302	.476	.573
ALDH1L1	.573	.002	.525	.622
SOX2	.645	.000	.600	.689
NANOG	.543	.067	.494	.591
BIRC5	.647	.000	.602	.692

TERT	.572	.002	.525	.619
HPV16-E7	.709	.000	.673	.746
HPV16-E14	.720	.000	.684	.756
HPV16-E6I	.701	.000	.664	.738

The test result variable(s): StrongestHPV.1, MCM2, Topo2A, MKi67, SOX2, BIRC5, TERT, HPV16-E7, HPV16-E1^4, HPV16-E6*I has at least one tie between the positive actual state group and the negative actual state group.

b. Null hypothesis: true area = 0.5

Result: Biomarkers with AUC <0.5 have been excluded from the presentation (AGR2, MMP7, GDA, CD63, POU5FI). All biomarkers with AUC >0.5 contribute to the discrimination of the two stages “normal epithelium” vs “HPV infected epithelium”. The highest AUC is seen with strongestHPV.1 which is the main criterium discriminating the two stages. This is also seen with the HPV splice markers. Next p16^{ink4a} shows a high AUC as it is induced by HR-HPV infection. In contrast, the tumor markers and cancer stem cell markers show very low AUC as most samples were not from progressed patients.

Non-dysplastic vs dysplastic epithelium: Group 0/1 vs. 2-5 (analysis score2345)

HPV and biomarker contribution for the differentiation of the two groups 0-1 vs 2-5 is presented in Fig. 9.

Table 7:

Case Processing Summary

Score2345	Valid N (listwise)
Positive[a]	777
Negative	358
Missing	303

Larger values of the test result variable(s) indicate stronger evidence for a positive actual state.

a. The positive actual state is 2-5.

Table 8:

Area Under the Curve for Group 0/1 vs. 2-5

Test Result Variable(s)	Area	Asymptotic Sig.[b]	Asymptotic 95% Confidence Interval	
			Lower Bound	Upper Bound
StrongestHPV.1	.795	.000	.766	.824
p16	.712	.000	.680	.744

STMN1	.590	.000	.556	.625
MCM2	.603	.000	.569	.637
Topo2A	.595	.000	.560	.629
MKi67	.597	.000	.563	.632
p63	.562	.001	.525	.598
ALDH1L1	.539	.036	.501	.576
SOX2	.571	.000	.535	.608
BIRC5	.579	.000	.544	.615
TERT	.550	.006	.514	.586
HPV16-E7	.673	.000	.642	.705
HPV16-E1^4	.667	.000	.635	.699
HPV16-E6I	.667	.000	.635	.699

The test result variable(s): Strongest HPV.1, p16, Topo2A, MKi67, SOX2, BIRC5, TERT, HPV16-E7, HPV16-E1^4, HPV16-E6I has at least one tie between the positive actual state group and the negative actual state group. Statistics may be biased.

b. Null hypothesis: true area = 0.5

Result: Biomarkers with AUC <0.5 have been excluded from the presentation (ALDH1A1, NANOG, AGR2, MMP7, GDA, CD63, POU5FI). All biomarkers with AUC >0.5 contribute to the discrimination of the two stages “non-dysplastic epithelium” vs “dysplastic epithelium”. Their contribution is highly significant. The highest AUC is seen with StrongestHPV.1 where the extent of oncogene E7 expression but also HPV splice markers are the strongest criteria for discriminating the two stages. Next p16^{ink4a} shows a high AUC as it is induced by E7 of HR-HPV. In contrast the tumor markers and cancer stem cell markers show relatively low AUC as most samples were not from progressed patients.

Mild dysplasia (≤CIN1) vs high grade dysplasia (CIN2+): Group 0-2 vs. 3-5 (analysis score345)

HPV and biomarker contribution for the differentiation of the two groups 0-2 vs 3-5 is presented in Fig. 10.

Table 9:

Case Processing Summary

Score345	Valid N (listwise)
Positive[a]	644
Negative	491

Missing	303

Larger values of the test result variable(s) indicate stronger evidence for a positive actual state.

a. The positive actual state is 3-5.

Table 10:

Area Under the Curve for Group 0-2 vs. 3-5

Test Result Variable(s)	Area	Asymptotic Sig.[b]	Asymptotic 95% Confidence Interval	
			Lower Bound	Upper Bound
StrongestHPV.1	.767	.000	.739	.795
p16	.733	.000	.704	.762
STMN1	.631	.000	.599	.663
MCM2	.630	.000	.598	.662
Topo2A	.613	.000	.580	.645
MKi67	.615	.000	.582	.647
p63	.587	.000	.554	.621
ALDH1L1	.568	.000	.534	.602
SOX2	.563	.000	.529	.597
BIRC5	.583	.000	.550	.616
TERT	.528	.108	.494	.561
HPV16-E7	.687	.000	.657	.718
HPV16-E1^4	.671	.000	.640	.702
HPV16-E6I	.675	.000	.644	.706

The test result variable(s): StrongestHPV.1, p16, MCM2, Topo2A, MKi67, SOX2, BIRC5, TERT, HPV16-E7, HPV16-E1^4, HPV16-E6I has at least one tie between the positive actual state group and the negative actual state group.

b. Null hypothesis: true area = 0.5

Result: Biomarkers with an AUC <0.5 have been excluded from the presentation (ALDH1A1, NANOG, AGR2, MMP7, GDA, CD63, POU5FI). All biomarkers with AUC >0.5 contribute to the discrimination of the two stages “mild dysplasia” vs “high grade dysplasia”. Their contribution is highly significant. The highest AUC is seen with strongestHPV.1 where the extent of oncogene E7 expression but also HPV splice markers are the strongest criteria for discriminating the two

stages. Next p16[ink4a] shows a high AUC as it is induced by E7 of HR-HPV. In contrast, the tumor markers and cancer stem cell markers show relatively low AUC as most samples were not from progressed patients (very few cervical cancer patients were included).

Discrimination of ≤CIN2 vs CIN3+: Group 0-3 vs. 4-5 (analysis score45)

This represents an especially important distinction because potentially discriminating dysplasia that need not to be treated urgently (CIN2) from dysplasia that is obligatory treated (CIN3+). HPV and biomarker contribution for differentiation of the two groups 0-3 vs 4-5 is presented in Fig. 11.

Table. 11:

Case Processing Summary

Score45[a]	Valid N (listwise)
Positive[b]	370
Negative	765
Missing	303

Larger values of the test result variable(s) indicate stronger evidence for a positive actual state.

a. The test result variable(s): StrongestHPV.1 has at least one tie between the positive actual state group and the negative actual state group.

b. The positive actual state is 4-5.

Table 12:

Area Under the Curve for Group 0-3 vs. 4-5

Test Result Variable(s)	Area	Asymptotic Sig.[b]	Asymptotic 95% Confidence Interval Lower Bound	Asymptotic 95% Confidence Interval Upper Bound
StrongestHPV.1	.707	.000	.677	.738
p16	.747	.000	.716	.778
STMN1	.708	.000	.676	.741
MCM2	.693	.000	.659	.727
Topo2A	.695	.000	.662	.728
MKi67	.699	.000	.666	.732
p63	.646	.000	.612	.681
ALDH1A1	.563	.001	.526	.599
ALDH1L1	.595	.000	.561	.630

SOX2	.581	.000	.546	.616
POU5FI	.521	.245	.485	.557
BIRC5	.633	.000	.598	.668
TERT	.567	.000	.531	.603
HPV16-E7	.713	.000	.679	.747
HPV16-E1^4	.706	.000	.672	.740
HPV16-E6I	.717	.000	.682	.751

The test result variable(s): StrongestHPV.1, MCM2, Topo2A, MKi67, p63, ALDH1A1, SOX2, BIRC5, TERT, HPV16-E7, HPV16-E1^4, HPV16-E6I has at least one tie between the positive actual state group and the negative actual state group.

b. Null hypothesis: true area = 0.5

Result: Biomarkers with a AUC <0.5 have been excluded from the presentation (NANOG, AGR2, MMP7, GDA, CD63). All biomarkers with AUC >0.5 contribute to the discrimination of the two stages "≤CIN2" vs "CIN3+". Their contribution is highly significant. The highest AUC is seen with p16^{ink4a} followed by Stathmin and strongestHPV.1 and HPV splice markers. HPV oncogene expression is upregulated at this threshold. Also, tumor markers (BIRC5, TERT) and proliferation-associated markers (MCM2, Topo2a, MKi67) gain value to identify high grade lesions (CIN3+). Biomarkers gain importance because most samples in both groups compared are HPV positive.

CIN3 vs Cervical Cancer: Group 0-4 vs. 5 (analysis score5)

HPV and biomarker contribution for the differentiation of the two groups 0-4 vs 5 is presented in Fig. 12.

Table 13:

Case Processing Summary

Score5[a]	Valid N (listwise)
Positive[b]	40
Negative	1095
Missing	303

Larger values of the test result variable(s) indicate stronger evidence for a positive actual state.

a. The test result variable(s): STMN1 has at least one tie between the positive actual state group and the negative actual state group.

b. The positive actual state is 5.

Table 14: Area Under the Curve

Test Result Variable(s)	Area	Asymptotic Sig.[b]	Asymptotic 95% Confidence Interval	
			Lower Bound	Upper Bound
StrongestHPV.1	.838	.000	.783	.892
p16	.687	.000	.599	.776
STMN1	.871	.000	.811	.930
MCM2	.901	.000	.849	.953
Topo2A	.886	.000	.836	.937
MKi67	.895	.000	.845	.945
p63	.778	.000	.710	.846
CD63	.630	.005	.532	.728
ALDH1A1	.807	.000	.720	.893
ALDH1L1	.632	.004	.539	.725
SOX2	.675	.000	.577	.773
NANOG	.800	.000	.730	.869
POU5FI	.785	.000	.704	.866
BIRC5	.879	.000	.817	.940
TERT	.846	.000	.784	.908
HPV16-E7	.742	.000	.654	.830
HPV16-E1^4	.692	.000	.607	.776
HPV16-E6I	.810	.000	.749	.872

b. Null hypothesis: true area = 0.5

Result: At this threshold towards invasive disease the tumor markers (BIRC5, TERT) gain importance with a higher AUC than strongestHPV.1 because they are specifically expressed at this stage. The proliferation markers (MCM2, Topo2a, MKi67, STMN) as a hallmark of malignant disease show high AUC as well.

Example - Contribution of biomarker combinations to risk score for different dysplastic stages

In order to demonstrate the superiority of combination of different biomarkers as compared to the individual biomarkers a ROC analysis was performed and the AUC and sensitivity and specificity for a given stage of dysplasia detection calculated.

Discrimination of mild dysplasia (≤CIN1) vs high grade dysplasia (CIN2+): Group 0-2 vs. 3-5 (analysis score345)

The value of “strongestHPV.1” (score 345) is compared to the combination with “StrongestHPV.1” plus “p16^{ink4a}” (risk345scorekonHPV4) and to a combination of “StrongestHPV.1”, “p16^{ink4a}”, plus MCM2 (risk345scorekonHPV4a). HPV and biomarker contribution for the differentiation of the dysplasia groups is presented in Fig. 13.

Table 15: Case Processing Summary

Score345[a]	Valid N (listwise)
Positive[b]	644
Negative	491
Missing	303

Larger values of the test result variable(s) indicate stronger evidence for a positive actual state.

a. The test result variable(s): Riskscore345konHPV4 has at least one tie between the positive actual state group and the negative actual state group.

b. The positive actual state is 3-5.

Table 16: Area Under the Curve

Test Result Variable(s)	Area	Asymptotic Sig.[b]	Asymptotic 95% Confidence Interval Lower Bound	Asymptotic 95% Confidence Interval Upper Bound
StrongestHPV.1	.767	.000	.738	.795
Risk345scorekonHPV4	.798	.000	.772	.824
Risk345scorekonHPV4a	.799	.000	.773	.825

The test result variable(s): StrongestHPV.1, Risk345scorekonHPV4, Risk345scorekonHPV4a has at least one tie between the positive actual state group and the negative actual state group.

a. Under the nonparametric assumption

b. Null hypothesis: true area = 0.5

Table 17:

	Cut off	Sensitivity	Specificity
StrongestHPV.1	0	89.9%	45.4%
Risk345scorekonHPV4	33	90.1%	43.8%
Risk345scorekonHPV4a	35.35	90.1%	47.0%

Result: The analysis using only „strongestHPV.1" has an AUC of 0.767 while the individual AUC of p16 was 0.733 and of MCM2 was 0.630 (see "Area Under the Curve for Group 0-2 vs. 3-5"). Combining "strongestHPV.1" with p16 in "risk345scorekonHPV4" increases the AUC to 0.798 and thus contributes to the sensitivity from 89.9% to 90.1% albeit reduces specificity from 45.4% to 43.8%. Further addition of MCM2 ("risk345scorekonHPV4a") does not enhance sensitivity but enhances specificity from 43.8% to 47%.

Discrimination of ≤CIN2 vs CIN3+: group 0-3 versus 4-5 (analysis score 45)

A comparison of the value of „strongest HPV.1" to p16^{ink4a} alone "p16", and to a combination of "StrongestHPV.1" combined with the biomarkers p16, STMN, MCM2, HPV16E61 and HPV16E1^4. HPV and biomarker contribution for the differentiation of the dysplasia groups is presented in Fig. 14.

Table 18:

Case Processing Summary

Score45	Valid N (listwise)
Positive[a]	370
Negative	765
Missing	303

Larger values of the test result variable(s) indicate stronger evidence for a positive actual state.

a. The positive actual state is 4-5.

Table 19:

Area Under the Curve ≤CIN2 vs CIN3+ for biomarker combinations

Test Result Variable(s)	Area	Asymptotic Sig.[b]	Asymptotic 95% Confidence Interval	
			Lower Bound	Upper Bound
StrongestHPV.1	.707	.000	.676	.738
p16	.747	.000	.716	.778
Risk45scoreHPV	.820	.000	.794	.846

The test result variable(s): StrongestHPV.1, p16 has at least one tie between the positive actual state group and the negative actual state group.

b. Null hypothesis: true area = 0.5

Table 20:

	Cut off	Sensitivity	Specificity
StrongestHPV.1	0.59	90%	42.9%

p16	3.5	90%	32.4%
Risk45scoreHPV	22.57	90%	53.3%

Result: For the given threshold to discriminate ≤CIN2 vs CIN3+ is the most important as it may influence therapy decision making. The comparison of “StrongestHPV.1” to “p16” alone or a biomarker combination “Risk45scoreHPV” (combined “StrongestHPV.1” with the biomarkers p16, STMN, MCM2, HPV16E61, and HPV16E1^4) shows significantly increased AUC values than for the two best individual markers. While “StrongestHPV.1” indicates just a prevalent HPV infection that can be present in both dysplasia stages, the enhanced expression of “p16” is indicative for a transforming infection by HR-HPV. Increased proliferative activity (STMN, MCM2) and HPV splice marker expression are hallmarks of more severe disease. When the sensitivity was set to 90% for the sake of comparison it becomes obvious that remarkable specificity gains are achieved in the marker combination.

Discrimination of ≤CIN3 vs Cervical Cancer: group 0-4 versus 5 (analysis score5)

In the discrimination of non-invasive to invasive disease the two single biomarkers “strongestHPV.1” and “MCM2” were compared to a combination (“risk5scoreHPV”) calculated from the biomarker combination “StrongestHPV.1”, MCM2, ALDH1A1, NANOG, and HPV16E61. HPV and biomarker contribution for the differentiation of the dysplasia group and invasive cancer is presented in Fig. 15.

Table 21:

Case Processing Summary

Score5	Valid N (listwise)
Positive[a]	40
Negative	1095
Missing	303

Larger values of the test result variable(s) indicate stronger evidence for a positive actual state.

a. The positive actual state is 5.

Table 22:

Area Under the Curve ≤CIN3 vs Cervical Cancer for biomarker combinations

Test Result Variable(s)	Area	Asymptotic Sig.[b]	Asymptotic 95% Confidence Interval	
			Lower Bound	Upper Bound
StrongestHPV.1	.838	.000	.783	.892
MCM2	.901	.000	.849	.953

Risk5scoreHPV	.942	.000	.910	.973

b. Null hypothesis: true area = 0.5

Table 23:

	Cut off	Sensitivity	Specificity
StrongestHPV.1	5.1	90%	56.7%
MCM2	3.61	90%	60.8%
Risk5scoreHPV	2.4	90%	84.7%

Result: At this threshold discriminating non-invasive from invasive disease the proliferation-associated biomarker MCM2 has a higher AUC than the “strongestHPV.1”. The addition of tumor markers (ALDH1A1, NANOG) and HPV splice marker increase the AUC significantly. With the sensitivity set to 90% for the sake of comparison it can be appreciated that the specificity is markedly increased by the combination of the biomarkers in “risk5scoreHPV”.

Summary

With respect to the contribution of individual biomarkers, the ROC analysis of individual biomarkers has demonstrated the individual contribution to the ability to distinguish between disease stages or grades. Occasionally, the markers correlate, however, it could be shown that the “Strongest HPV” as well as $p16^{ink4a}$ are the most reliable biomarkers for the identification of lower grade lesions. However, they are not independent from each other, since P16 is upregulated by the expression of HR-HPV E7. They are good markers for lower grade stages or grades as long as proliferation specific and tumour specific markers are not upregulated.

Further, the proliferation associated biomarkers (MCM2, Topo2a, mKi-67, STMN) seem to be very close to each other in the ROC curve region. The third group of biomarkers relates to tumour or tumour stem cell markers (ALDH1A1, Sox, Nanog, Pou5FI, BIRC, TERT). These markers are expressed considerably stronger in lesions that are progressing and approaching the CxCA stage or have become invasive disease.

In comparison to the statistical power of the single marker “Strongest HPV“ alone, or all in view of the best biomarkers alone (p16 or MCM2), combinations of the risk scores of these markers in the ROC analysis show better results with a higher AUC and increased sensitivity and specificity. As some markers may be functionally redundant and not independent from each other, as discussed above, not all of them show an additive contribution, but may be exchanged against one another. The combination of the best markers of the individual groups increases sensitivity and specificity of the classification of samples into their respective disease grades.

Example RT-qPCR

The comparable detection of mRNA quantity by an alternative to the QuantiGene method (here RT-qPCR) is shown as an input value for the calculation of risk score values (to be adapted).

<u>RNA-Isolation:</u>

Total RNA of liquid-based cervical cytology samples was isolated using the QiaSymphony (Qiagen). The protocol includes a DNAse treatment step. The RNA content of each sample was calculated using a micro volume spectrometer (NanoDrop, ThermoFisher).

Reverse Transcription:

For the first-strand cDNA synthesis, we used the Promega GoScript kit with a combination of random primers and oligo (dT)-primers. The reaction condition and thermocycling program used are shown in tab. 24.

Tab. 24: A) Reaction condition of the cDNA synthesis using the Promega GoScript Kit.
B) Thermocycling program.

A)

Component	*Amount/concentration*
5x Go Script Reaction Buffer	4 µl
MgCl2	4 µl (5 mM)
PCR Nucleotid Mix	1 µl
Random primers	0.5 µl (0.5 ng)
Oligo (dT) primers	0.5 µl (0.5 ng)
GoScript Reverse Transcriptase	1 µl
RNase Inhibitor	0.5 µl
Template RNA	100 ng
RNAse-free water	Add. 20 µl

B)

Temperature (°C)	*Duration (min.)*
25	10
42	30
70	10
4	∞

RT-qPCR:

For the quantification of the cDNA, a SYBR-Green dye-based assay was used. The reaction efficiency was calculated for each primer pair. Using a standard curve, all amplification efficiencies were in the acceptable range of 90-110%. The specificity of the reaction was reviewed by melting curve analysis for every single reaction. Table 25 shows the reaction condition and thermocycling program for the qPCR using the Promega GoTaq (SYBR-Green) Master Mix and the 7500 fast Real-Time PCR System (Applied Biosystems). The whole cDNA was diluted 1:5 in RNAse-free water before use.

Tab. 25: A) Reaction conditions of the qPCR using the GoTaq (SYBR Green) Master Mix.
B) Thermocycling program of the 7500 fast Real-Time System.

A)

Component	*Amount/concentration*
GoTaq qPCR MasterMix	12.5 µl
Primer forward	2.5 µl (0.2 µM)
Primer reverse	2.5 µl (0.2 µM)
RNAse-free water	2.5 µl
cDNA	5 µl (diluted cDNA)

B)

Temperature (°C)	*Duration (min.)*	
95	2	
95	0:15	40x
60	1	
60-95	-	

For further analysis, we used the automatically calculated threshold and quantification cycle from the analysis software (Applied Biosystems HRM Software). For ΔCq-Calculation, the Cq from the reference gene (ACTB) was subtracted from the Cq of the sample. The Cq for a sample without calculable Cq was set to 40 for further analysis.

The relative expression values obtained by QuantiGene and RT-qPCR of the same samples comprising stage 0 to 5 were correlated for markers E7 for HPV16, E7 for HPV18, STMN, MCM2, mKi-67, BIRC5, and ALDH1A1. Correlation coefficients were generally >0.5. This represents a "good" correlation of expression strength measured by the two alternative methods.

Description of RT-qPCR method and validation

RT-qPCR validation of 10 informative biomarkers for risk score development:

Validation of the quantification of biomarkers with the QuantiGene by the gold standard RT-qPCR. The quantification of biomarkers by the QuantiGene is comparable to the quantification by RT-qPCR. The measured RT-qPCR data can be used to differentiate between the clinical stages.

Method:

Samples representing different stages of dysplasia progression were identified and measured both by QuantiGene and by RT-qPCR. Forward and reverse primers were selected and optimized from the standardized primer collection of Sigma-Aldrich (Cat. No. KSPQ12012G).

Statistical analysis was performed with the R Core Team programme (2017). (R: A language and environment for statistical computing. R Foundation for Statistical Computing,Vienna, Austria. URL (https://www.R-project.org/).

Processing of the QuantiGene data:

For the quantitative evaluation of the QuantiGene measurement, the measured raw data (MFI = median fluorescent intensity) was modified as follows:

1. Subtraction of the background (mean value of the three negative controls that are included in each measurement)

2. Normalization to ACTB

3. Multiplication by 100 for easier handling of the data (unit of the data now rMFI)

If the measured ACTB values of the sample was below 100 MFI (ACTB<100 MFI) the sample was excluded from the evaluation, due to insufficient cellular content.

Processing of the RT-qPCR data:

For the quantitative evaluation of the RT-qPCR data, the Ct value of each sample was determined using the measurement software (7500 SDS v1.4.1, ThermoFisher). Each value was measured in duplets, i.e. twice, and the mean value was calculated. If the Ct values of the duplet was more than 10% apart, the measurement was repeated. If the variation during the repetition was still more than 10%, the sample was excluded from the evaluation. To check the specificity of the primer pairs, a melting curve analysis was also performed after each measurement. The samples were also excluded, if the melting curve analysis yields more than one amplification product. In addition, the Ct values were subsequently normalized to ACTB (Δ Ct value).

Primer sequences employed for the RT-qPCR analysis:

Marker	Forward Primer (5´-3´)	Reverse Primer (5´-3´)	Origin
**ACTB**	GACGACATGGAGAAAATCTG	ATGATCTGGGTCATCTTCTC	Sigma Aldrich
**MCM2**	ATCTACAGAACTACCAGCG	GAGCCATCATAGTTGTTGTG	Sigma Aldrich

**STMN1**	AAATGGCAGAAGAGAAACTG	CTTCAATGTGCTTATCCTTCTC	Sigma Aldrich
**HPV16-E7**	CAAGTGTGACTCTACGCTTCGG	GTGCCCATTAACAGGTCTTCCAA	Cattani et a. 2009*
**HPV18-E7**	TAATCATCAACATTTACCAGCCC G	CGTCTGCTGAGCTTTCTACTACTA	Cattani et a. 2009*
**MKI67**	CAAGAAAGACAAAAAGCCA	CTTGACACACACATTGTCCT	Sigma Aldrich
**ALDH1A1**	GACAATGGAGTCAATGAATGG	ATCAATTGGTATTGTACGGC	Sigma Aldrich
**P16/CDKN2 A**	GGGGGCACCAGAGGCAGT	GGTTGTGGCGGGGGCAGTT	von Keyserlin g et al. 2011**
**BIRC5**	TGCCCCGACGTTGCC	CAGTTCTTGAATGTAGAGATGCG GT	Sigma Aldrich
**TERT**	TTATGTCACGGAGACCAC	CAAGTGCTGTCTGATTCC	Sigma Aldrich

*Cattani, P., et al. (2009). "RNA (E6 and E7) assays versus DNA (E6 and E7) assays for risk evaluation for women infected with human papillomavirus." J Clin Microbiol 47(7): 2136-2141.

**von Keyserling, H., et al. (2012). "p16INK(4)a and p14ARF mRNA expression in Pap smears is age-related." Mod Pathol 25(3): 465-470.

<u>**Comparison of the RT-qPCR data with the QuantiGene data:**</u>

Comparing the RT-qPCR data with the QuantiGene data, correlation coefficients between -0.33 and -0.9 (r_S) were observed (Table 26, Figure 17). The best correlation between RT-qPCR data and QuantiGene data was observed for Ki67 (-0.81), the worst for TERT (-0.33). r_S is negative because in RT-PCR the lower the Δct value the more RNA is in the sample. In QuantiGene the higher the rMFI, the higher is the RNA content.

Tab. 16 Spearman´s rank correlation coefficients for the tested biomarkers

Marker	r_S	p-Value
**STMN1**	-0.75	< 0.0001
**MCM2**	-0.73	< 0.0001
**Ki67**	-0.81	< 0.0001
**BIRC5**	-0.57	< 0.0001

**UBC**	-0.51	< 0.0001
**ALDH1A1**	-0.76	< 0.0001
**CDKN2A/p16**	-0.67	< 0.0001
**TERT**	-0.33	0.0202
**HPV16**	-0.61	< 0.0001
**HPV18**	-0.58	< 0.0001

Correlation of the markers and the clinical stages:

The expression of the biomarkers STMN1, MCM2, Ki67 and p16 increases over the clinical stages CIN I to CxCa (Figure 18). The leading HPV increases until CIN III and is then steady. The expression of HPV16, HPV18, ALDH1A1 and BIRC5 increases until CIN III and then drops a bit for the CxCa.

Using the Spearman rank correlation coefficient (r_S), the data set was tested for the existence of a continuous correlation between the expression strength of the biomarkers and the disease stages. The best correlation was found for the leading HPV (r_S = -0,623) followed by MCM2 (r_S = -0,459). The worst correlation was found for HPV18-E7 (r_S = -0,299) (Table 27) (due to restricted numbers of positive samples available).

Tab. 27: Spearman´s rank correlation for Biomarker expression against the clinical group

**Biomarker**	r_s	**p-value**	**n=**
**Leading HPV**	-0,623	<0,001	75
**MCM2**	-0,459	<0,001	73
**P16**	-0,448	<0,001	66
**BIRC5**	-0,441	<0,001	74
**TERT**	-0,432	<0,001	53
**HPV16-E7**	-0,407	<0,001	75
**Ki67**	-0,401	<0,001	72
**STMN1**	-0,385	<0,001	75
**ALDH1A1**	-0,347	<0,001	69
**HPV18-E7**	-0,299	0,009	75

Receiver operating characteristic (ROC Analysis):

ROC analyses were performed to decide how well the biomarkers are suited to differentiate between the clinical stages.

Clinical Stage CIN2+:

For the CIN2+ threshold, the leading HPV (AUC= 0,885) followed by BIRC5 (AUC= 0,771) is best for differentiating between clinical groups. The lowest AUC has HPV18-E7 (0,674). The best Cut Off was calculated using the Youden-method, with the sensitivity and specificity of each biomarker (Table 28). All ROC curves have a statistical significance (p<0.05) for this level (Figure 19).

Tab. 28: AUC, Cut Off and associated sensitivity and specificity of the ROC curves for all biomarkers regarding the disease threshold CIN2+

Biomarker	AUC	CI (95%)	CutOff (Youden)	Sensitivity	Specificity
ALDH1A1	0.6951014	0.570-0.820	9.785375	0.4594595	0.875
MCM2	0.7356061	0.621-0.850	8.651925	0.575	0.8787879
BIRC5	0.7713235	0.666-0.877	10.69365	0.7	0.7058824
STMN1	0.6921429	0.572-0.812	5.98235	0.55	0.8
P16	0.723206	0.599-0.848	6.984925	0.9459459	0.4482759
Ki67	0.69375	0.572-0.816	11.61225	0.75	0.59375
TERT	0.7378788	0.603-0.873	15.22915	0.8484848	0.55
HPV16-E7	0.7264286	0.612-0.841	9.614725	0.575	0.8285714
HPV18-E7	0.6742857	0.553-0.796	13.45402	0.375	0.9142857
Strongest HPV	0.8557143	0.771-0.941	9.614725	0.85	0.7714286

Clinical Stage CIN3+:

For the CIN3+ threshold, the MCM2 (AUC= 0,783) followed by the leading HPV (AUC= 0,779) is best for differentiating between clinical groups. The lowest AUC has HPV18-E7 (0,609). The best Cut Off was calculated using the Youden-method, with the sensitivity and specificity (Table 29). All ROC curves except HPV18-E7 have a statistical significance (p<0.05) for this level (Figure 20).

Tab. 29: AUC, Cut Off and associated sensitivity and specificity of the ROC curves of all biomarkers for the disease threshold CIN3+

Biomarker	AUC	CI (95%)	CutOff (Youden)	Sensitivity	Specificity
ALDH1A1	0.7045855	0.569-0.840	9.785375	0.5555556	0.8571429
MCM2	0.7826087	0.670-0.896	8.321115	0.6666667	0.8478261

**BIRC5**	0.750985	0.632-0.870	8.8582	0.4814815	0.9148936
**STMN1**	0.7731481	0.653-0.894	5.8283	0.6666667	0.7916667
**P16**	0.7717073	0.654-0.889	3.59605	0.56	0.8780488
**Ki67**	0.69375	0.572-0.816	11.61225	0.7777778	0.6444444
**TERT**	0.7521739	0.613-0.891	9.4626	0.4782609	0.9666667
**HPV16-E7**	0.6666667	0.534-0.799	15.74852	0.7407407	0.5833333
**HPV18-E7**	0.6087963	0.470-0.748	8.094825	0.3333333	0.9375
**Strongest HPV**	0.7785494	0.666-0.891	7.75955	0.7777778	0.75

<u>**Clinical Stage CxCa:**</u>

For the CxCa border, p16 (AUC= 0,735) followed by the leading HPV (AUC= 0,727) is best for differentiating between clinical groups. The lowest AUC has ALDH1A1 (0,452). The best Cut Off was calculated using the Youden-method, with the sensitivity and specificity (Table 30). Only the ROC curve of ALDH1A1 is uninformative for this stage (Figure 21), although this may be improved simply by assessing more samples.

Tab. 30: AUC, CutOff and associated sensitivity and specificity of the ROC curves of all biomarkers for the disease threshold CxCa

**Biomarker**	**AUC**	**CI (95%)**	**CutOff (Youden)**	**Sensitivity**	**Specificity**
**ALDH1A1**	0.4518519	0.164-0.740	16.85283	0.3333333	0.916667
**MCM2**	0.6597222	0.413-0.906	6.906	0.5555556	0.875
**BIRC5**	0.6068376	0.359-0.854	7.271725	0.4444444	0.9384615
**STMN1**	0.6750842	0.459-0.891	4.557675	0.5555556	0.8030303
**P16**	0.7349138	0.529-0.941	1.95255	0.5	0.9310345
**Ki67**	0.6719577	0.486-0.858	11.03637	0.7777778	0.5555556
**TERT**	0.675	0.419-0.931	9.742725	0.625	0.7777778
**HPV16-E7**	0.6195286	0.444-0.795	15.74852	0.8888889	0.5151515
**HPV18-E7**	0.6144781	0.369-0.860	6.121375	0.4444444	0.9393939
**Strongest HPV**	0.7272727	0.589-0.865	9.614725	1	0.5

Conclusion:

mRNA quantification by QuantiGene 2.0 plex assay is reliable and reproducible by RT-qPCR and both assays could be alternative methods for mRNA expression profiling for cervical dysplasia diagnosis. The biomarkers correlate well with the clinical stages and ROC curves show that most of them individually are informative for the differentiation of the clinical groups. This also validates the QuantiGene data. Logistic regression analyses with the RT-qPCR data, in order to develop risk scores, are expected to show comparable usefulness to the QuantiGene data.

<u>Prognostic value of Risk scores</u>

The following describes the evaluation of a prognostic value of the molecular profiling by biomarkers of clinical samples by QuantiGene for their future development. This is termed QuantiGene molecular profiling histology (QG-MPH).

Women referred to colposcopy were recruited. Clinical data were collected from medical health records including cytology, histology and standard HPV-test results.

A QuantiGene assay (QuantiGene 2.0 platform, ThermoFisher) was performed on cervical smear samples. Risk scores for CIN2+ and CIN3+ from QuantiGene assay were calculated and compared to actual histological endpoints CIN2+ and CIN3+. The risk scores derived from samples at baseline were compared to histological diagnoses obtained >3 months after initial samples in patients who had not had any intervention.

Comparison of QG-MPH risk scores to diagnosis in this referral population had a sensitivity/specificity for CIN2+ of 76.1%/56.1% and for CIN3+ of 86.5%/37.9%, respectively. Regarding prospective histologic findings as endpoints, sensitivity and specificity was similar with 77.0% /55.4% for CIN2+, 90.2%/39.2% for CIN3+, respectively. The sub-analysis of 31 follow-ups after initial CIN2 diagnosis showed that the QuantiGene risk score from the first visit corresponded with CIN2 lesion development. In progressive/stable/regressive lesions the QG-MPH risk score CIN2+ was positive in 92.9%/70.6%/58.3%, respectively. It was negative in progressive/stable/regressive lesions in 7.1%/29.4%/41.7%, respectively.

QuantiGene risk scores correlated with development of untreated dysplastic lesions and therefore show a prognostic value.

<u>CIN2-Analysis:</u>	**V2 (>3 months)**		
	**<CIN2**	**CIN2**	**>CIN2**
**V1 CIN2 (baseline)**	**n=24**	**n=17**	**n=14**
	Regression	*Persistency*	*Progression*
**RS CIN2+ positive (n)**<br>*Proportion in histologic group*	**10**<br>**58,3%**	**12**<br>**70,6%**	**13**<br>**92,9%**
RS CIN2+ neg. (n)<br>*Proportion in histologic group*	**14**<br>**41,7%**	**5**<br>**29,4%**	**1**<br>**7,1%**
**RS CIN3+ pos. (n)**	**15**	**12**	**13**

Proportion in histologic group	**62,5%**	**70,6%**	**92,9%**
RS CIN3+ neg. (n)<br>*Proportion in histologic group*	**9**<br>**37,5%**	**5**<br>**29,4%**	**1**<br>**7,1%**

CLAIMS

1. An in vitro method for determining the severity or a grade of a human papillomavirus (HPV)-induced dysplasia or the presence of cervical carcinoma in a subject, comprising:

   a) quantitatively determining an expression level of (i) viral and (ii) cellular messenger RNA (mRNA) in a sample obtained from the subject, wherein

      i) the determined viral mRNA encodes an HPV oncoprotein E6 and/or E7, and

      ii) wherein the determined cellular mRNA comprises:

         - mRNA of at least one cellular proliferation marker, and
         - mRNA of at least one cancer stem cell marker, and
         - mRNA of at least one tumor marker, and

   b) deducing from the quantity of said viral mRNA and said cellular mRNA the severity or a grade of the dysplasia or whether cervical carcinoma is present in the subject.

2. The in vitro method according to claim 1, wherein the method comprises additionally quantitatively determining an expression level of the mRNA of at least one housekeeping gene and normalizing the determined expression level of the viral and cellular mRNA to the expression level of the at least one housekeeping gene.

3. The in vitro method according to any one of claims 1 or 2, wherein the severity or grade of dysplasia is determined by comparing the quantified (absolute and/or normalized) expression levels of viral and cellular mRNA to predetermined threshold values for severities or grades of HPV-induced dysplasia and the presence of cervical carcinoma.

4. The in vitro method according to the preceding claim, wherein quantified (absolute and/or normalized) expression levels of viral and cellular mRNA indicate a severity or grade of dysplasia or the presence of cervical carcinoma when said levels of mRNA are above predetermined statistically established threshold values, wherein an expression level

   - above a first threshold corresponds to stage CIN2,
   - above a second threshold corresponds to stage CIN3, and
   - above a third threshold corresponds to cervical carcinoma.

5. The in vitro method according to any one of the preceding claims, wherein the viral mRNA encoding an HPV oncoprotein E6 and/or E7 is selected from the group consisting of spliced mRNA E6*I, E1C, and E1^4.

6. The in vitro method according to any one of the preceding claims, wherein the viral mRNA encoding an HPV oncoprotein E6 and/or E7 is selected from the group consisting of a HPV genotype selected from HPV 6, 16, 18, 26, 31, 33, 35, 39, 45, 51, 52, 53, 56, 58, 59, 66, 68, 73 and 82.

7. The in vitro method according to any one of the preceding claims, wherein viral mRNA encoding HPV E6 and/or E7 is determined for multiple potential HPV genotypes (infections), and the strongest HPV genotype is employed in deducing the severity or grade of the dysplasia, wherein the strongest HPV genotype is the only HPV genotype or the HPV genotype with the highest expression when multiple HPV genotypes (infections) are present.

8. The in vitro method according to any one of the preceding claims, wherein

   a) the at least one cellular proliferation marker is selected from the group consisting of P16, MCM2, Topo2a, STMN1, Ki-67 and mKi-67, preferably consisting of P16, STMN1 and MCM2,

   b) the at least one cancer stem cell marker is selected from the group consisting of Sox, Nanog, POU5FI/Oct3/4, ALDH1A1 and ALDH1L1, preferably ALDH1A1, and/or

   c) the at least one tumor marker is selected from the group consisting of BIRC5, TERT and p53, preferably BIRC5 and TERT.

9. The in vitro method according to any one of the preceding claims, wherein the following markers are employed to determine a CIN2+ grade of dysplasia: strongest HPV genotype, HPV16 E6*I, HPV16 E1^E4, CDKN2A/P16, and STMN1, wherein the strongest HPV genotype is the only HPV genotype or the HPV genotype with the highest expression level when multiple HPV genotypes (infections) are present.

10. The in vitro method according to any one of the preceding claims, wherein the following markers are employed to determine a CIN3+ grade of dysplasia: strongest HPV genotype, HPV16 E6*I, HPV 16 E1^E4, P16 and MCM2.

11. The in vitro method according to any one of the preceding claims, wherein the following markers are employed to determine the presence of cervical carcinoma: strongest HPV genotype, BIRC5, TERT, ALDH1A1 and MCM2.

12. The in vitro method according to any one of the preceding claims, wherein said in vitro method comprises the following steps:

   a) quantitatively determining an expression level of (i) viral and (ii) cellular messenger RNA (mRNA) in a sample obtained from the subject, wherein

      i. the determined viral mRNA encodes an HPV oncoprotein E6 and/or E7, selected from the group consisting of the strongest HPV genotype, HPV16 E6*I and HPV16 E1^E4, wherein the strongest HPV genotype is the only HPV genotype or the HPV genotype with the highest expression level when multiple HPV genotypes (infections) are present, and

      ii. the determined cellular mRNA comprises:

- mRNA of at least one cellular proliferation marker, selected from the group consisting of P16, STMN1 and MCM2, and
- mRNA of at least one cancer stem cell marker, including ALDH1A1, and
- mRNA of at least one tumor marker, selected from the group consisting of BIRC5 and TERT, and
- mRNA of at least one housekeeping gene, and

b) deducing from the quantity of said viral mRNA and said cellular mRNA the severity or a grade of the dysplasia or whether cervical carcinoma is present in the subject, wherein

i. the following markers are employed to determine a CIN2+ grade of dysplasia: strongest HPV genotype, HPV16 E6*I, HPV16 E1^E4, P16, and STMN1,

ii. the following markers are employed to determine a CIN3+ grade of dysplasia: strongest HPV genotype, HPV16 E6*I, HPV 16 E1^E4, P16 and MCM2, and

iii. the following markers are employed to determine the presence of cervical carcinoma: strongest HPV genotype, BIRC5, TERT, ALDH1A1 and MCM2,

wherein

c) the severity or grade of dysplasia is determined by comparing the quantified expression levels of viral and cellular mRNA to predetermined thresholds for severities or grades of HPV-induced dysplasia and the presence of cervical carcinoma.

13. The in vitro method according to any one of the preceding claims, wherein the quantitative determining of the expression of viral and cellular mRNA in the sample of a subject is performed as single step method.

14. The in vitro method according to any one of the preceding claims, wherein the quantitative determining of the expression of viral and cellular mRNA in the sample of a subject is performed using solid phase-bound probe-directed capture of a target mRNA, preferably QuantiGene, or RT-qPCR.

15. The in vitro method according to any one of the preceding claims, wherein the method comprises additionally determining the HPV type in said subject having a HPV infection.

16. The in vitro method according to any one of the preceding claims, wherein the method comprises additionally predicting the risk of the subject developing cervical carcinoma.

17. The in vitro method according to any one of the preceding claims, wherein the amount of viral and cellular mRNA, preferably the normalized expression level of said viral and cellular mRNA, is introduced into a mathematical algorithm that combines said amounts and provides a score value suitable for determining the severity and/or a grade of the dysplasia or whether cervical carcinoma is present in the subject.

18. A computer readable storage medium comprising instructions to configure a processor to perform a method and/or algorithm of mathematical evaluation for the generation of a mathematical model based on the quantitative mRNA expression levels determined in a method according to any one of the preceding claims for the characterization of a sample based on evaluation of the mRNA expression of the markers, wherein characterization means analysis and/or retrieving predictive information and/or profiling based on molecular mRNA expression and/or comparing results of quantitative mRNA expression analysis according to a standard.

19. A computer readable storage medium according to the preceding claim, wherein dichotomization is used to determine cut-off values for the severities or grades of HPV-induced dysplasia or the presence of cervical carcinoma according to the method of any one of claims 1 to 17, comprising grouping samples into clinical groups/clinical scores.

20. A computer readable storage medium according to the preceding claim, suitable for analyzing data obtained from cervical smear samples, and wherein the clinical groups/clinical scores comprise:

- Clinical group 0, is defined as HPV negative and histologically without pathological findings,
- Clinical group 1 is defined as HPV positive and histologically without pathological findings,
- Clinical group 2 is defined as HPV positive and histologically CIN1 and
- Clinical group 3 is defined as HPV positive and histologically CIN2 and
- Clinical group 4 is defined as HPV positive and histologically CIN3 and
- Clinical group 5 is defined as HPV positive and histologically cancerous,

wherein these clinical groups are used for dichotomization into clinical thresholds,

- particularly wherein clinical threshold CIN2+ is defined as separating the clinical groups 0-2 from the clinical groups 3-5, and
- clinical threshold CIN3+ is defined as separating the clinical groups 0-3 from the clinical groups 4-5, and
- the clinical threshold of carcinomas is defined as separating the clinical groups 0-4 from the clinical group 5.

21. A computer readable storage medium comprising instructions to configure a processor to perform a method and/or algorithm of mathematical evaluation for the processing of quantitative mRNA expression levels (based on raw or relativized data) obtained by a method according to any of claims 1-17, wherein predictive values are calculated for the respective markers that exceed the predictive value of single marker cut-offs, and a risk stratification score is calculated based on the quantities of the cellular and viral mRNA and a mathematical evaluation combining the predictive values of analyzed cellular and viral markers, preferably wherein said marker values are dichotomized into cut-off values for the stratification of the risk of the subject to develop a malignant transformation.

Figure 1

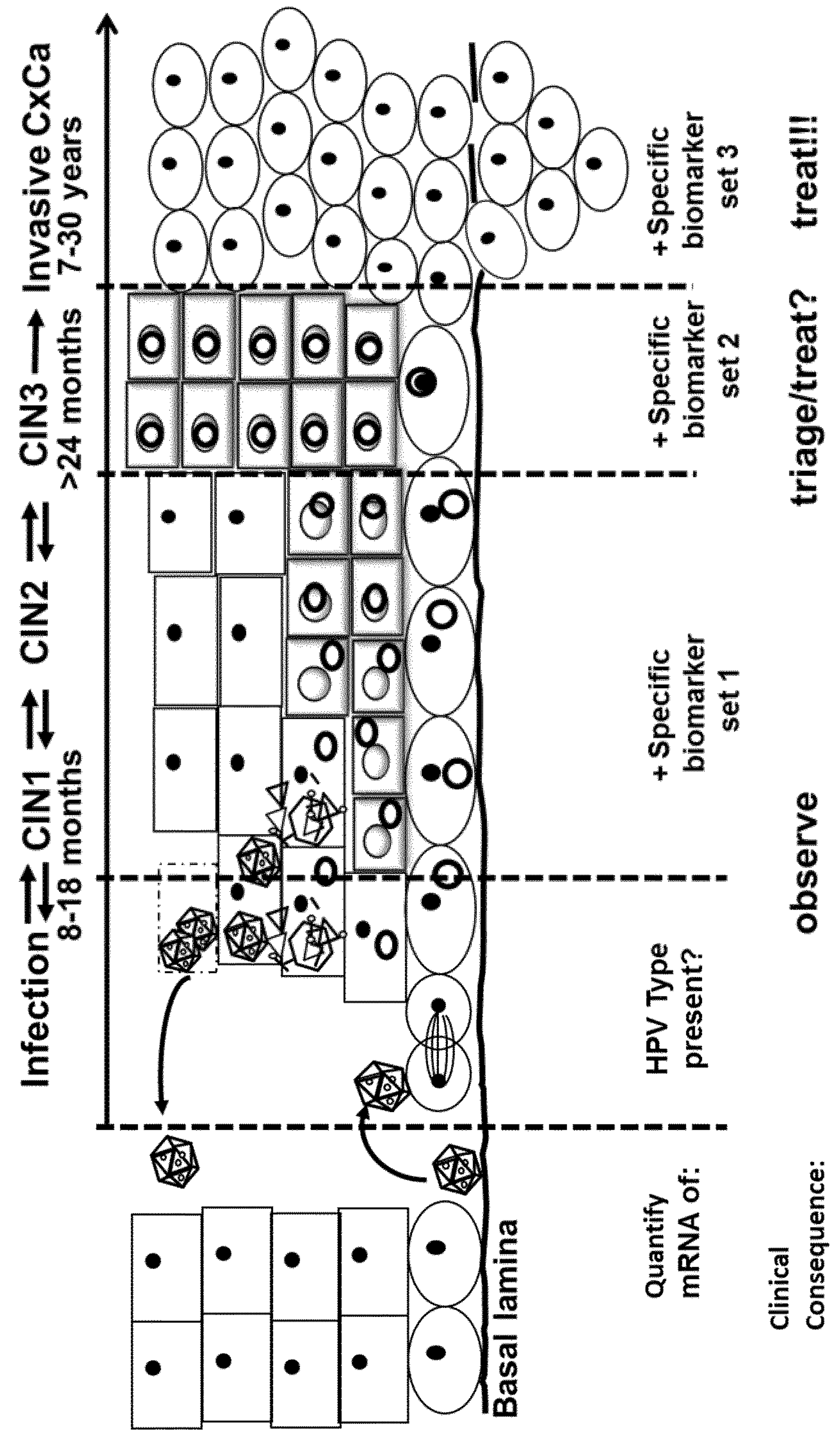
Infection
CIN1
CIN2
CIN3
Invasive CxCa
8-18 months
>24 months
7-30 years
Basal lamina
Quantify mRNA of:
HPV Type present?
+ Specific biomarker set 1
+ Specific biomarker set 2
+ Specific biomarker set 3
Clinical Consequence:
observe
triage/treat?
treat!!!

Figure 2:

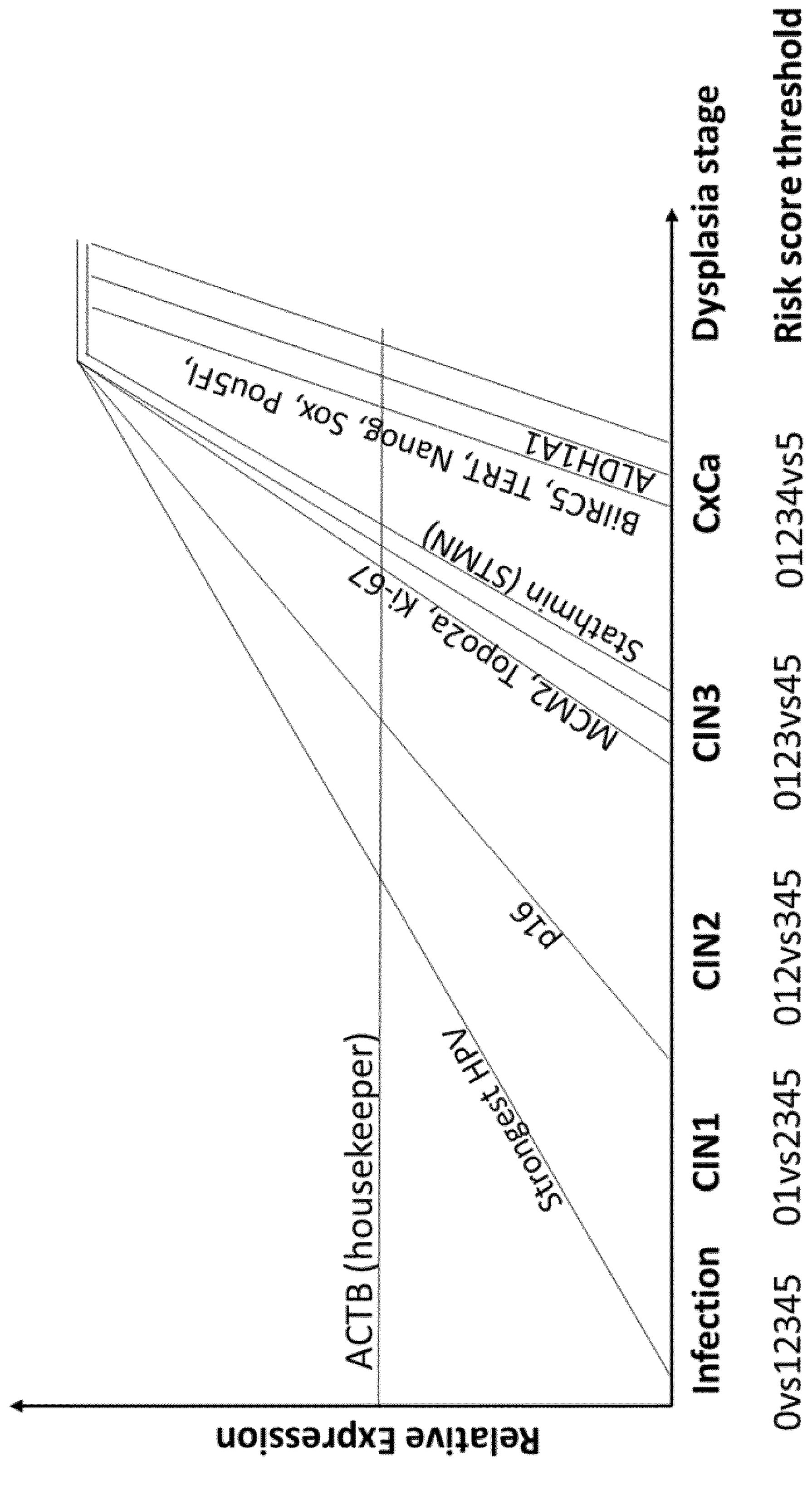
Relative Expression
Dysplasia stage
Risk score threshold
Infection
CIN1
CIN2
CIN3
CxCa
0vs12345
01vs2345
012vs345
0123vs45
01234vs5
ACTB (housekeeper)
Strongest HpV
p16
MCM2, Topo2a, Ki-67
Stathmin (STMN)
BiIRC5, TERT, Nanog, Sox, Pou5Fl,
ALDH1A1

Figure 3:

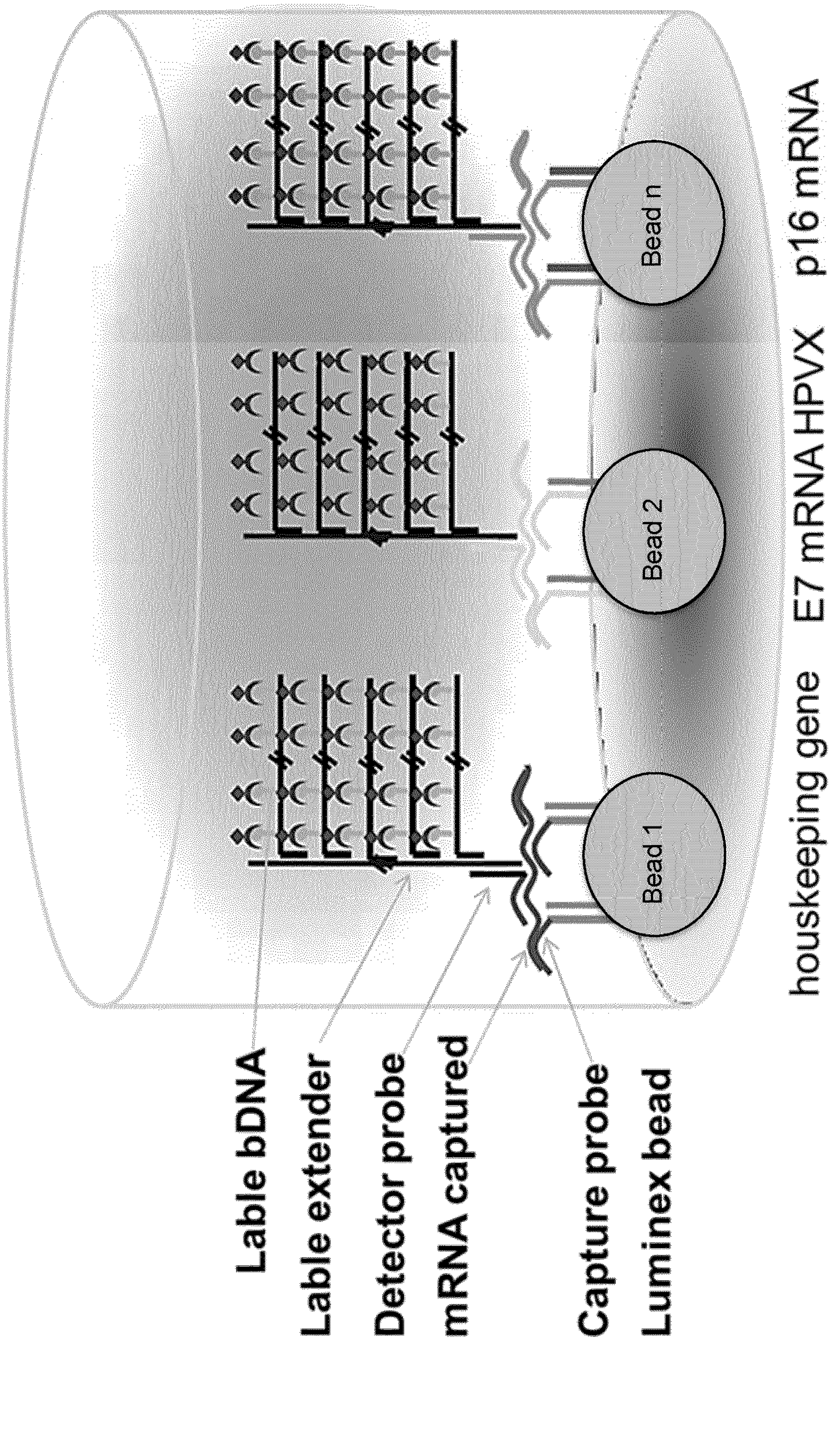
Bead 1
Bead 2
Bead n
housekeeping gene
E7 mRNA HPVX
p16 mRNA
Lable bDNA
Lable extender
Detector probe
mRNA captured
Capture probe
Luminex bead

Figure 4:

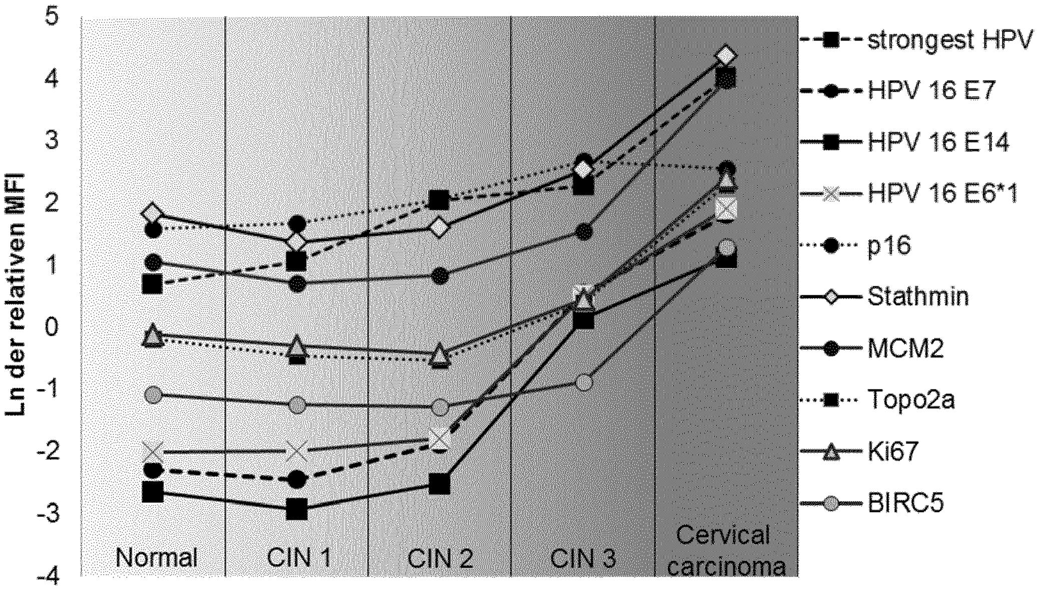

Figure 5:

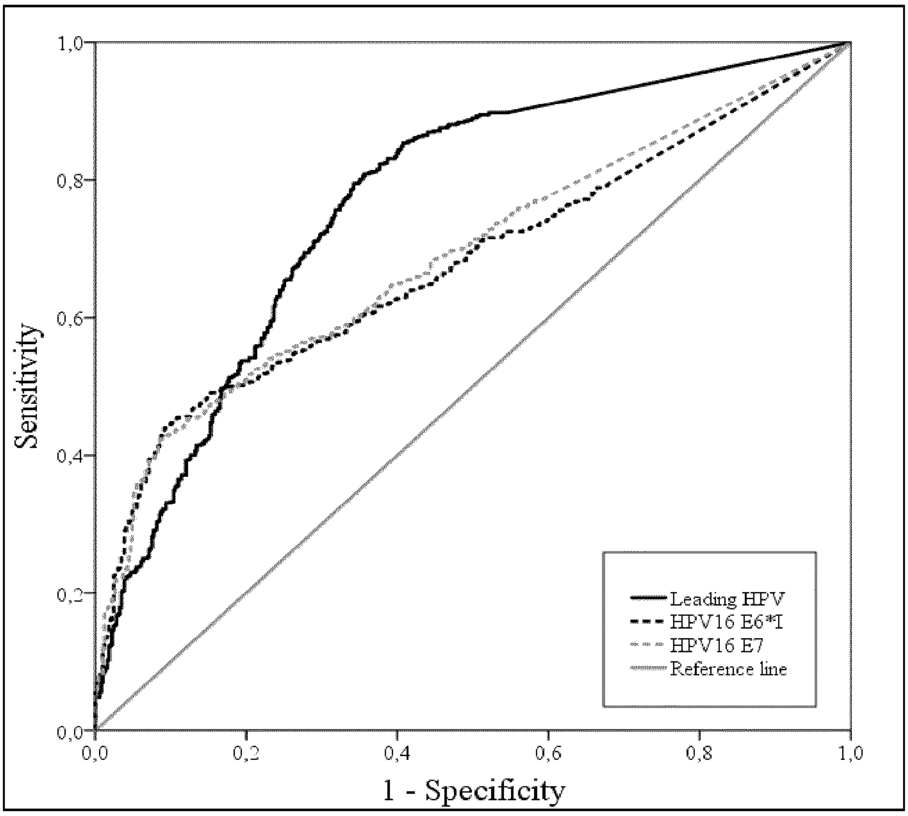

Figure 6:

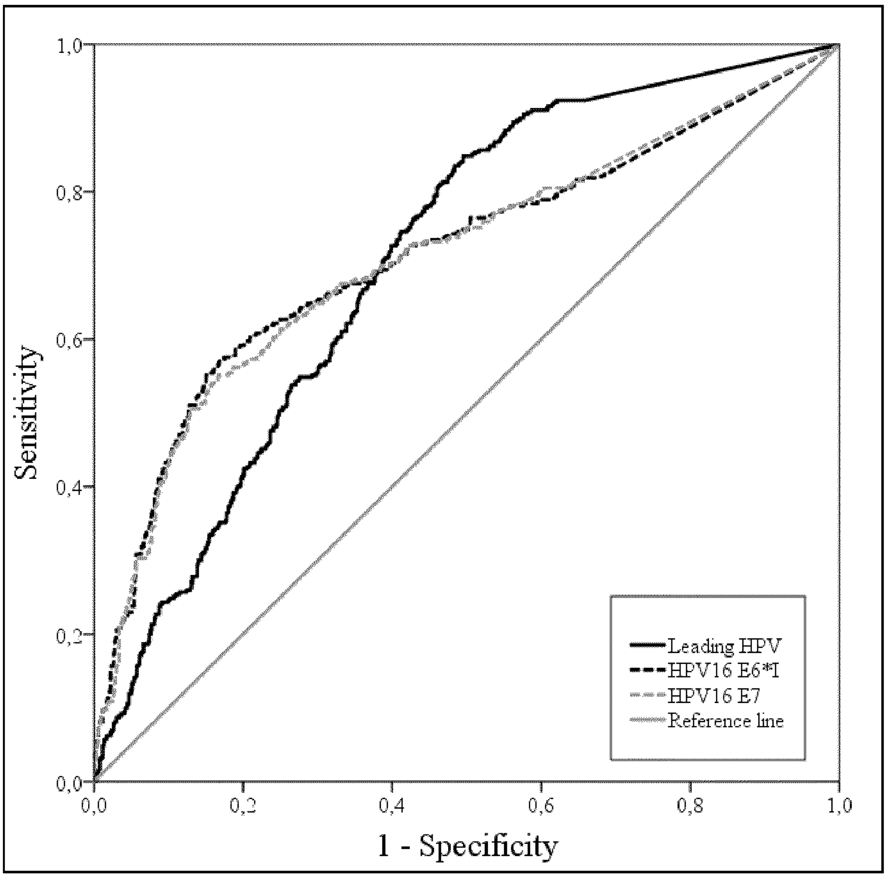

Figure 7:

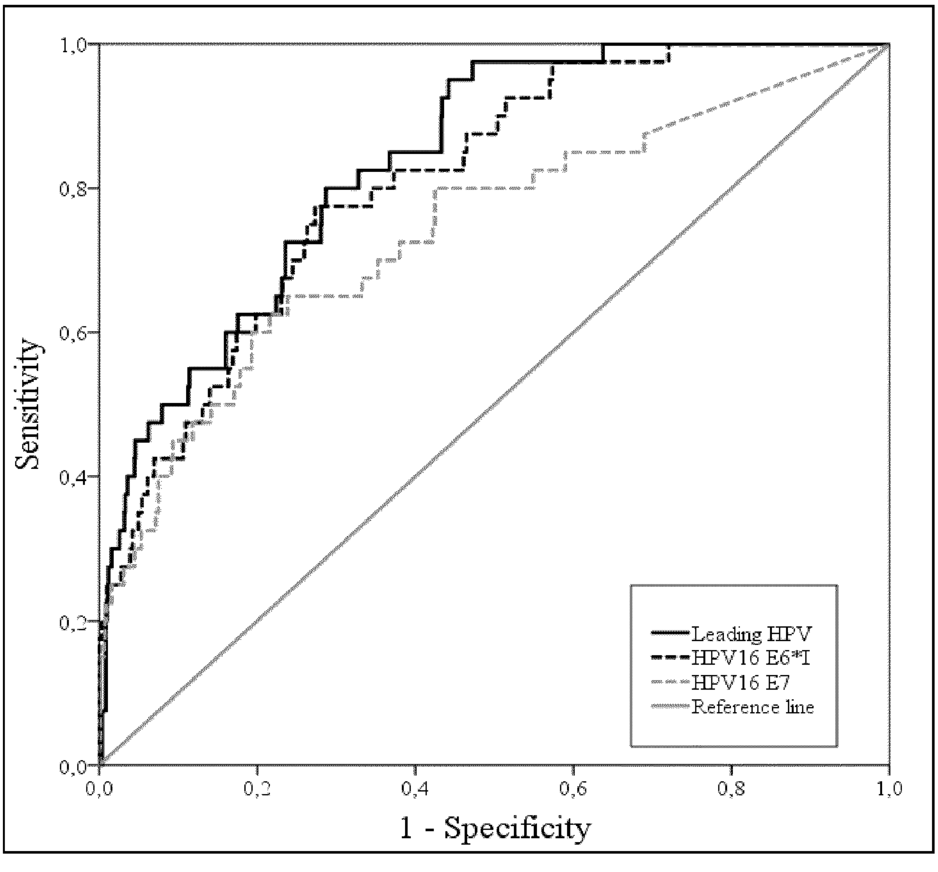

Figure 8:

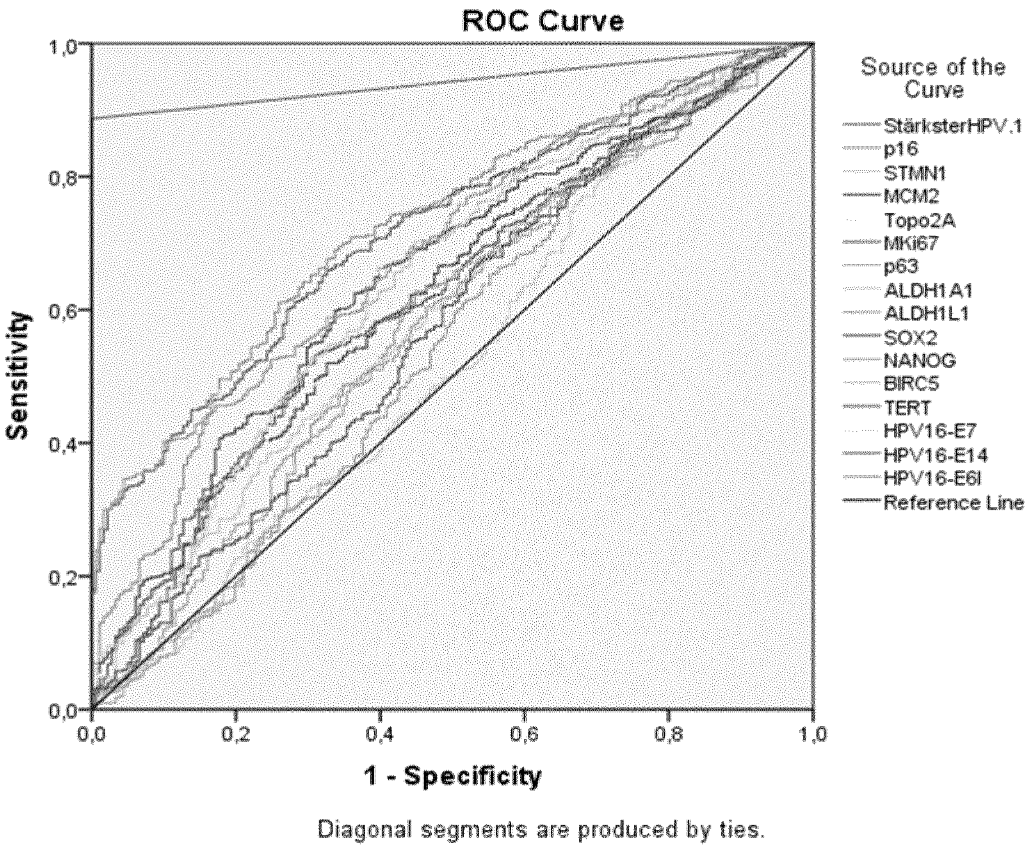

Figure 9:

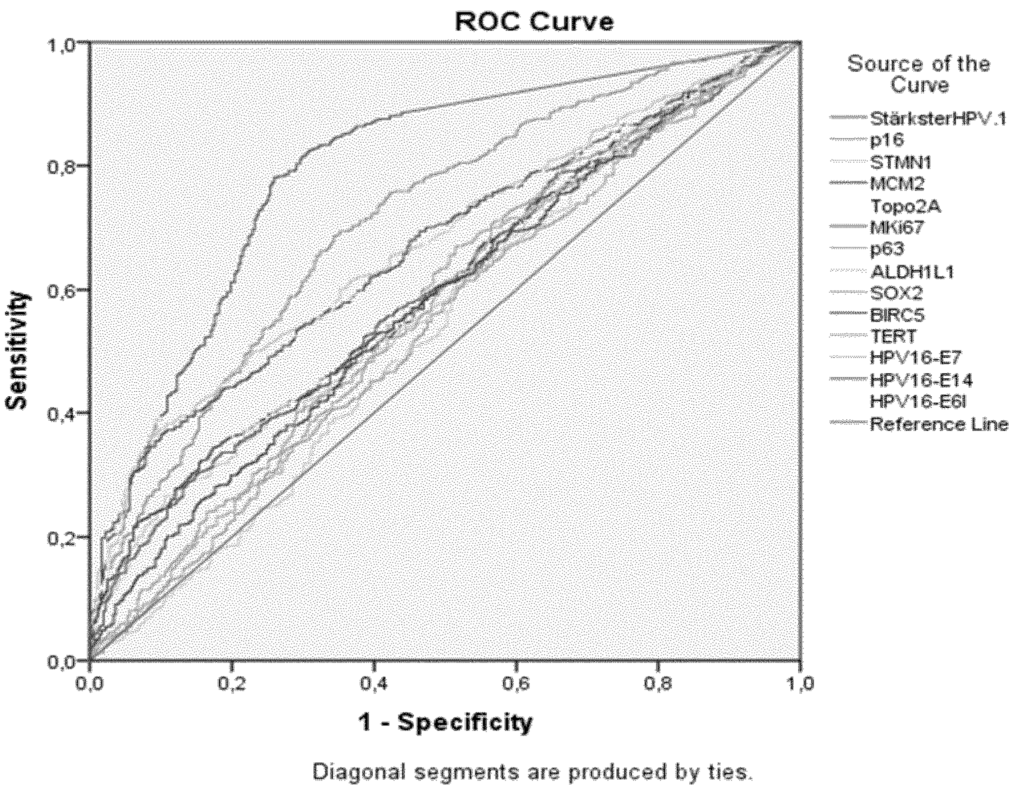

Figure 10:

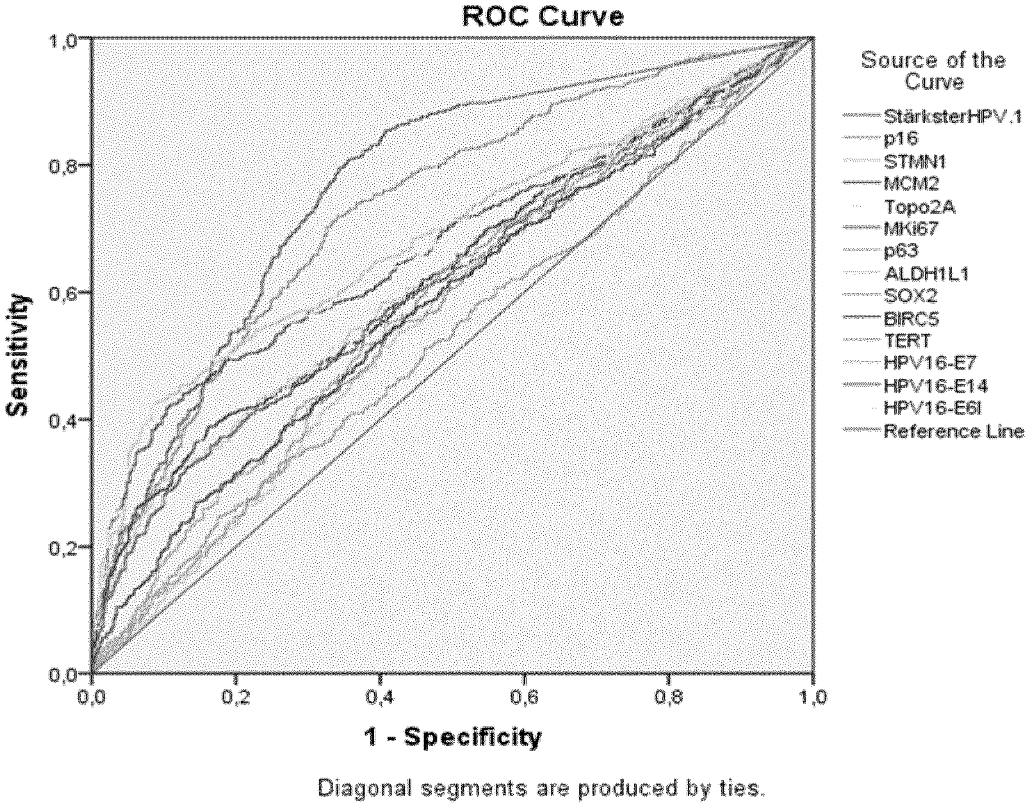

Figure 11:

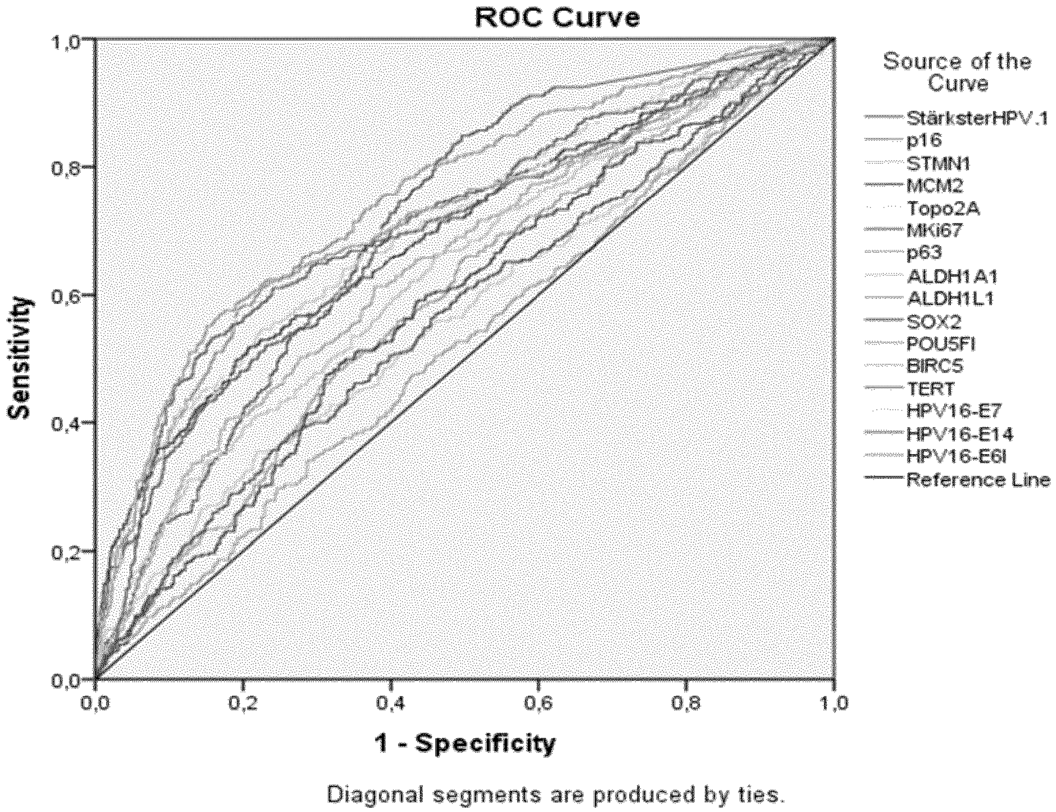

Figure 12:

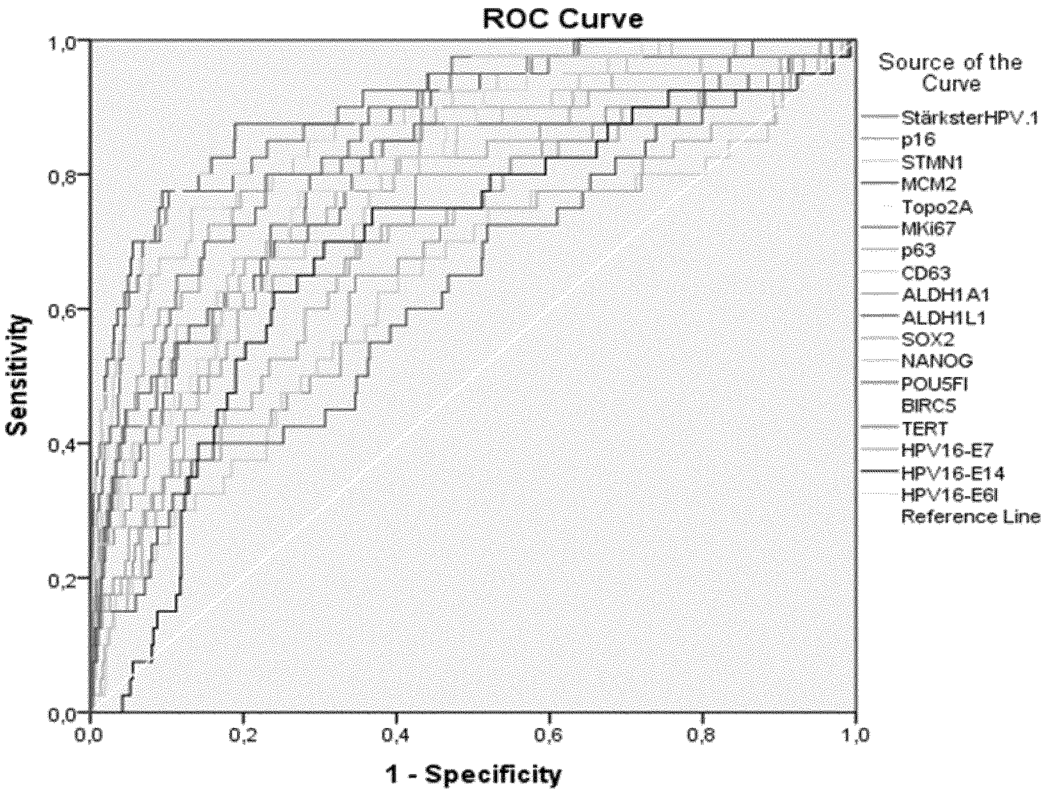

Figure 13:

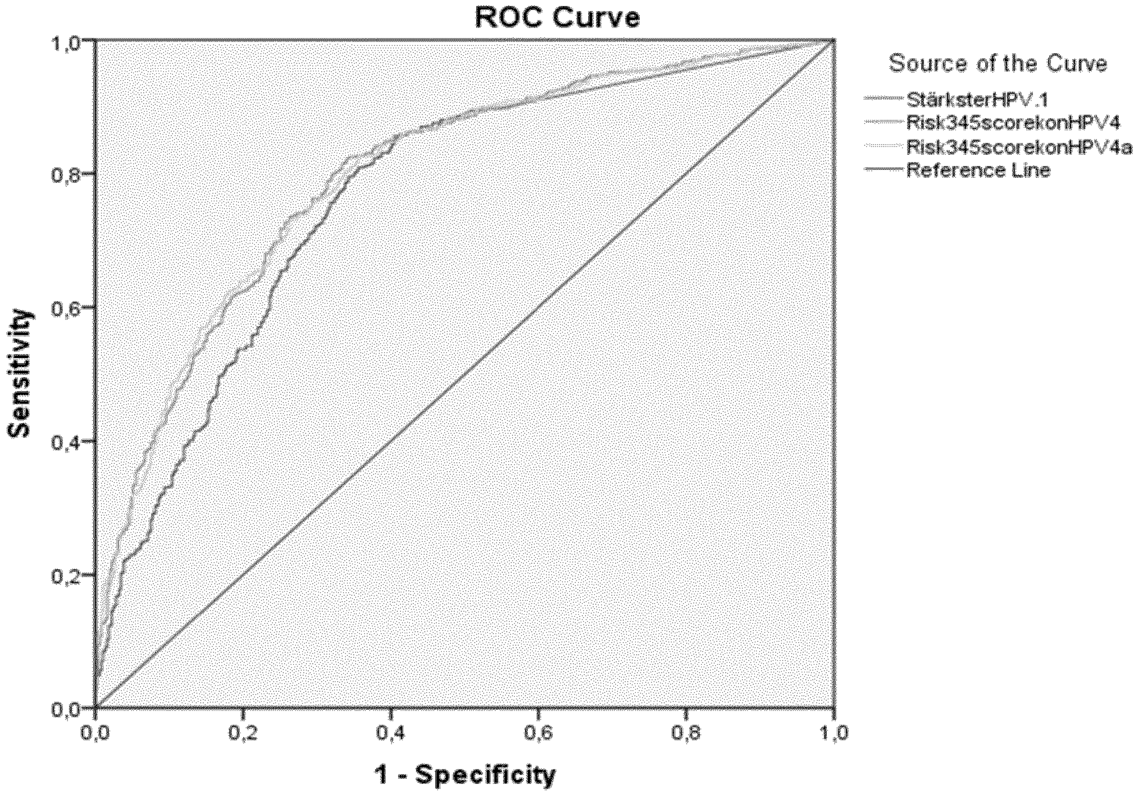

Figure 14:

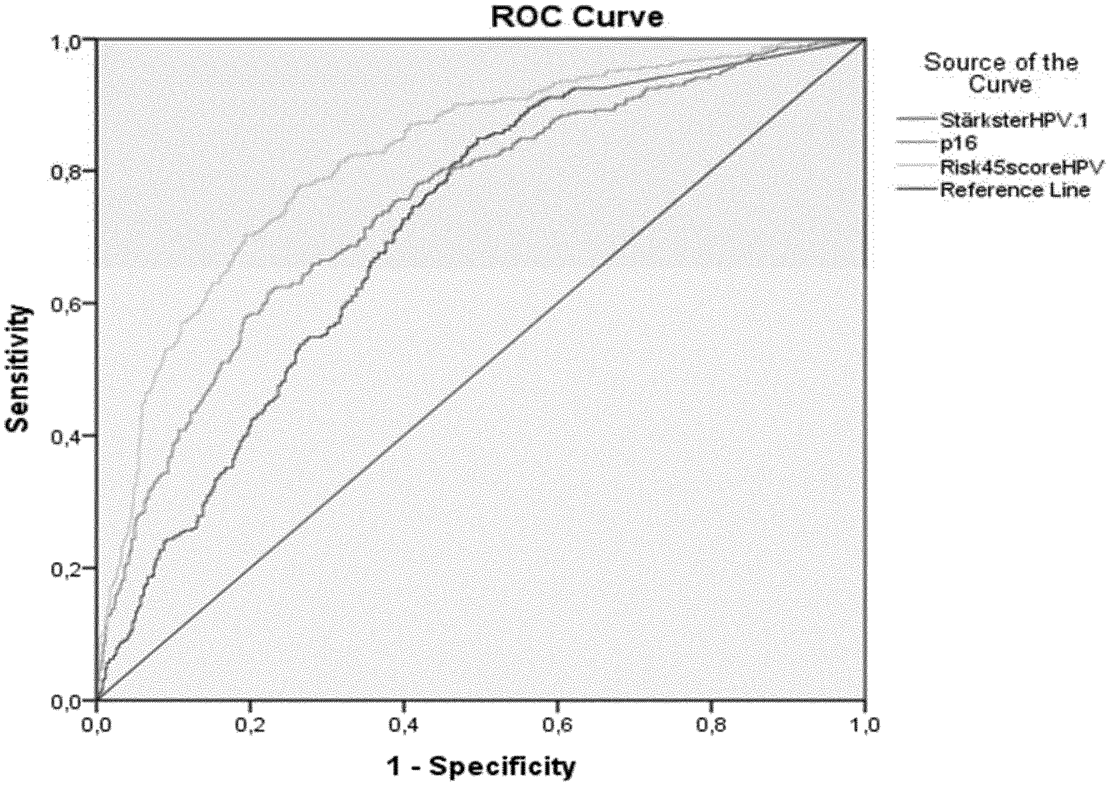

Diagonal segments are produced by ties.

Figure 15:

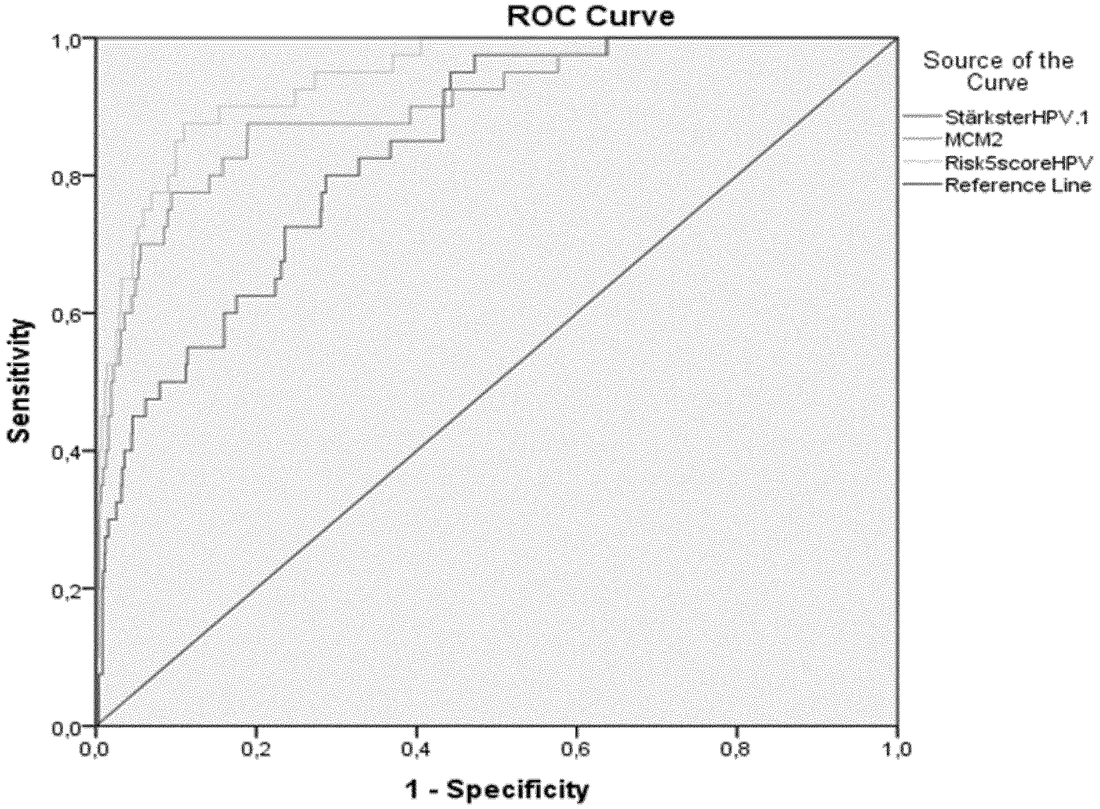

Figure 16

A

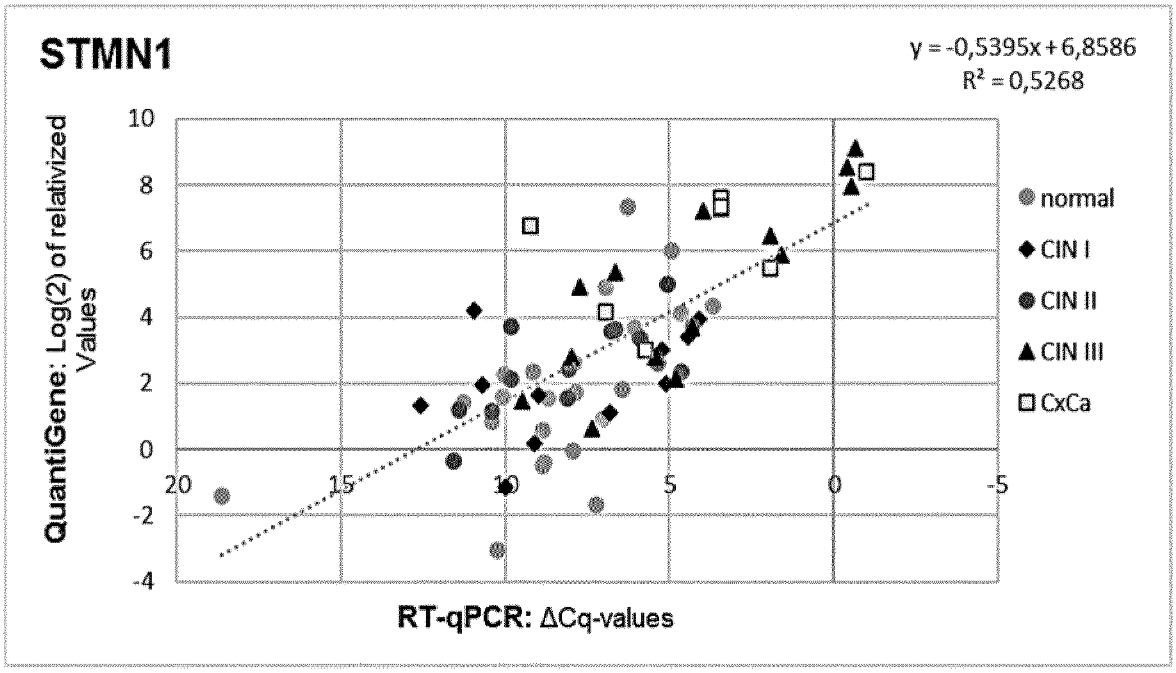
STMN1
y = -0,5395x + 6,8586
R² = 0,5268
QuantiGene: Log(2) of relativized Values
RT-qPCR: ΔCq-values
normal
CIN I
CIN II
CIN III
CxCa
10
8
6
4
2
0
-2
-4
20
15
10
5
0
-5

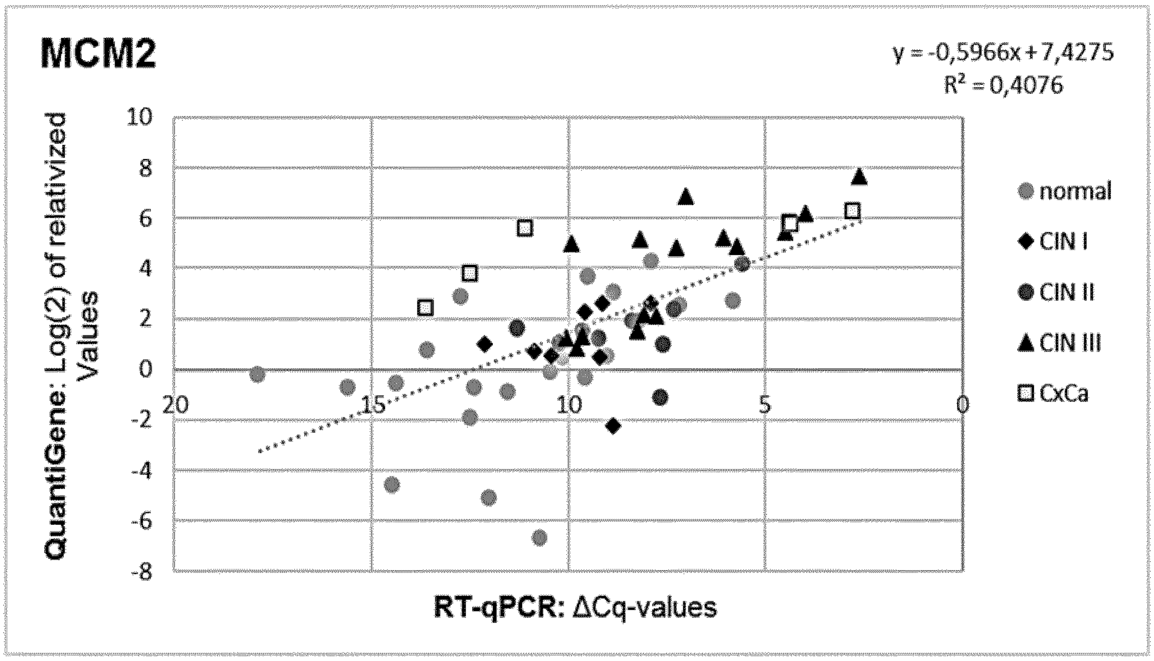
MCM2
y = -0,5966x + 7,4275
R² = 0,4076
QuantiGene: Log(2) of relativized Values
RT-qPCR: ΔCq-values
normal
CIN I
CIN II
CIN III
CxCa
10
8
6
4
2
0
-2
-4
-6
-8
20
15
10
5
0

Fig. 16 (cont.)

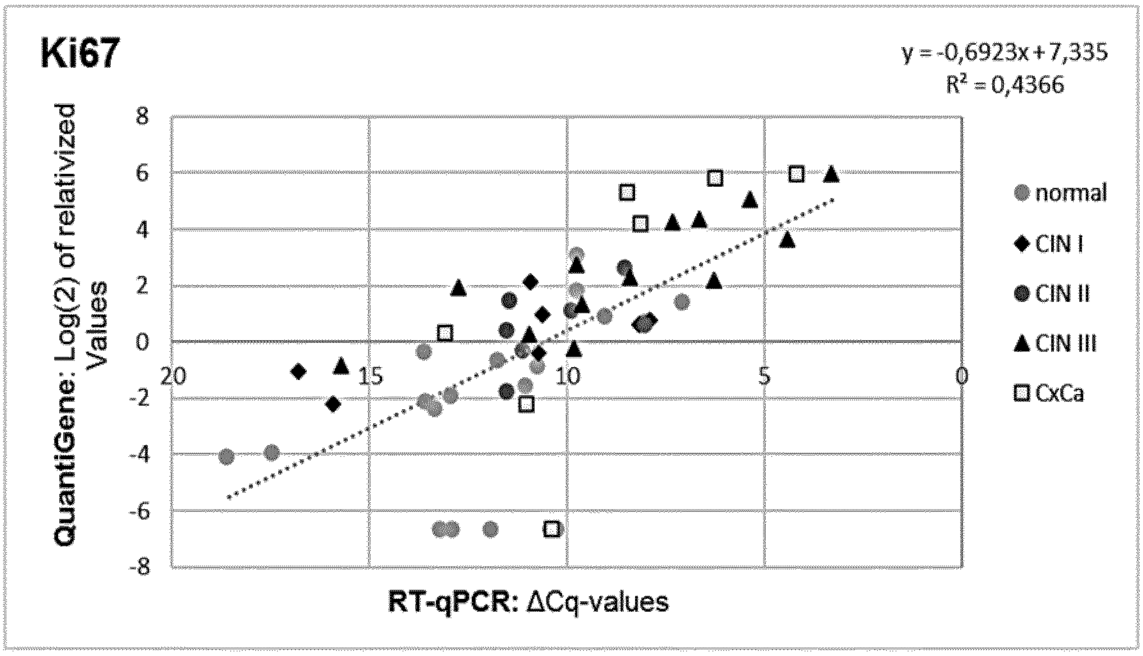
Ki67
y = -0,6923x + 7,335
R² = 0,4366
QuantiGene: Log(2) of relativized Values
RT-qPCR: ΔCq-values
8
6
4
2
0
-2
-4
-6
-8
20
15
10
5
0
normal
CIN I
CIN II
CIN III
CxCa

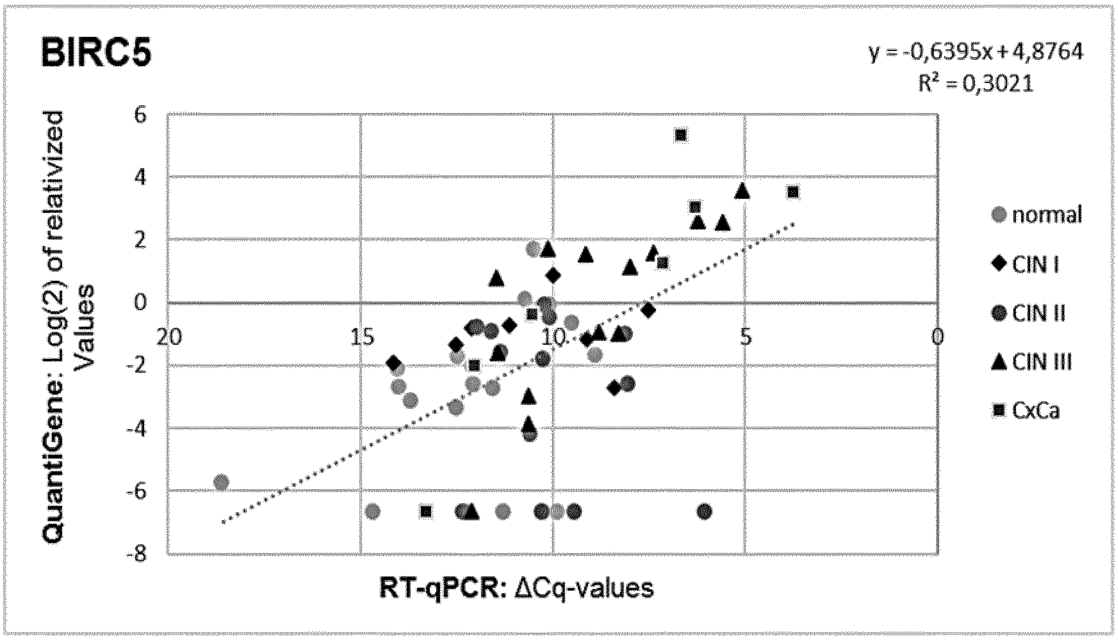
BIRC5
y = -0,6395x + 4,8764
R² = 0,3021
QuantiGene: Log(2) of relativized Values
RT-qPCR: ΔCq-values
6
4
2
0
-2
-4
-6
-8
20
15
10
5
0
normal
CIN I
CIN II
CIN III
CxCa

Fig. 16 (cont.)

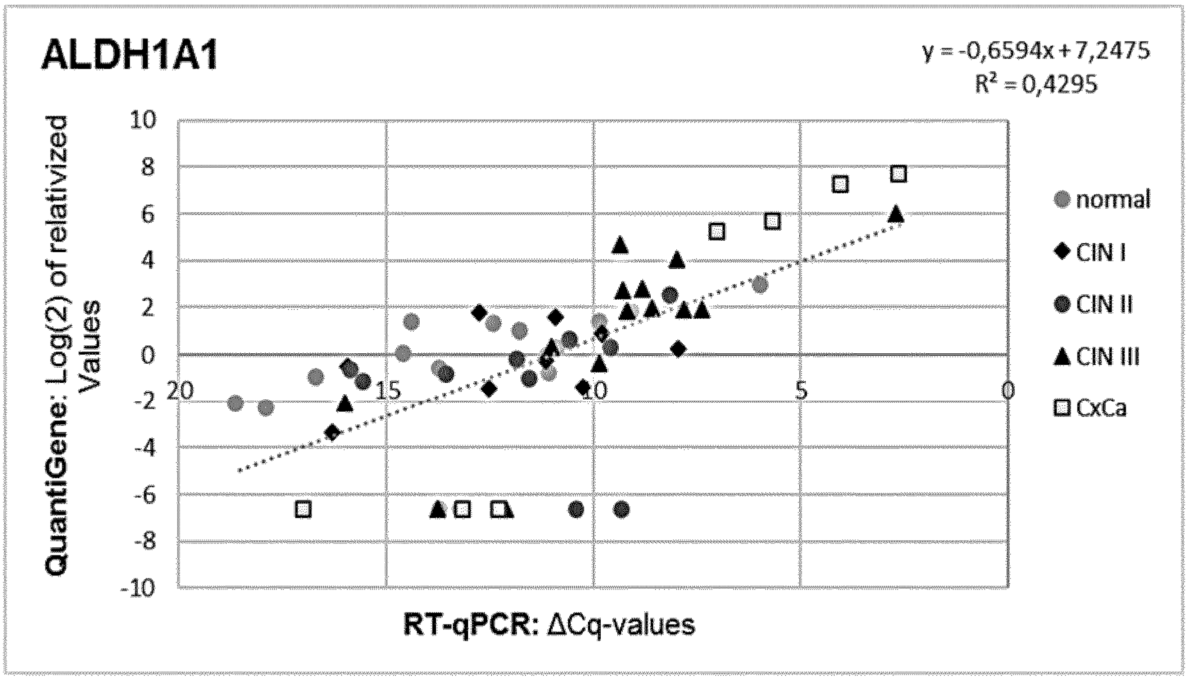
ALDH1A1
y = -0,6594x + 7,2475
R² = 0,4295
QuantiGene: Log(2) of relativized Values
RT-qPCR: ΔCq-values
normal
CIN I
CIN II
CIN III
CxCa

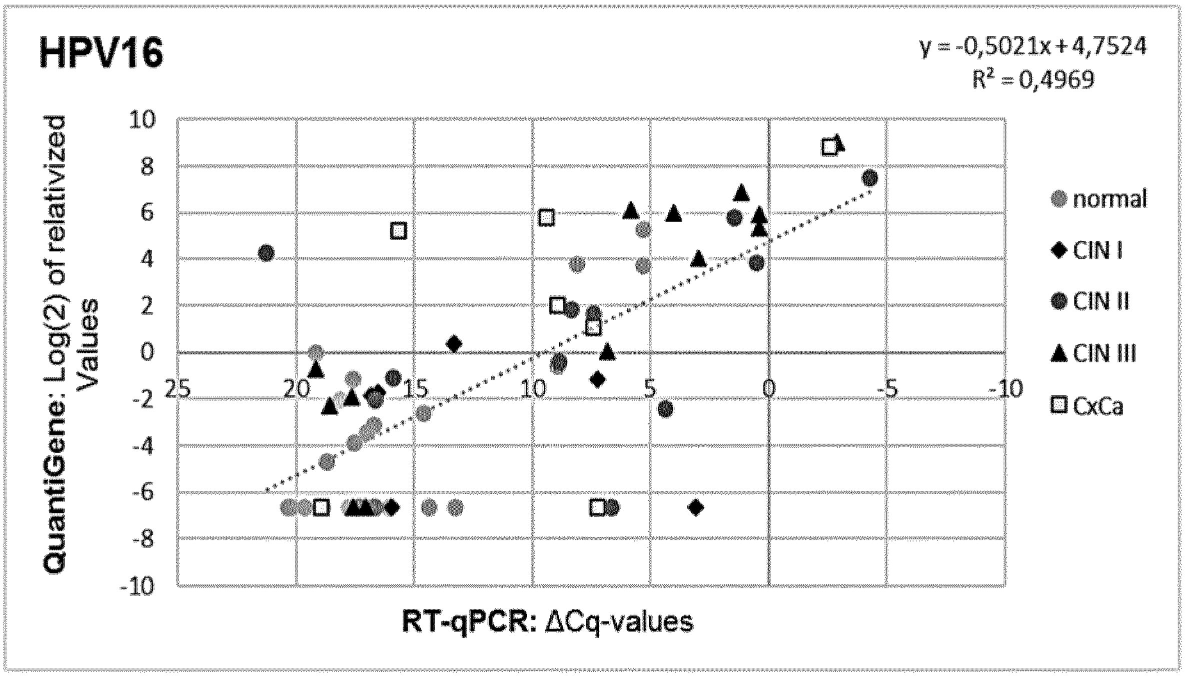
HPV16
y = -0,5021x + 4,7524
R² = 0,4969
QuantiGene: Log(2) of relativized Values
RT-qPCR: ΔCq-values
normal
CIN I
CIN II
CIN III
CxCa

Fig. 16 (cont.)

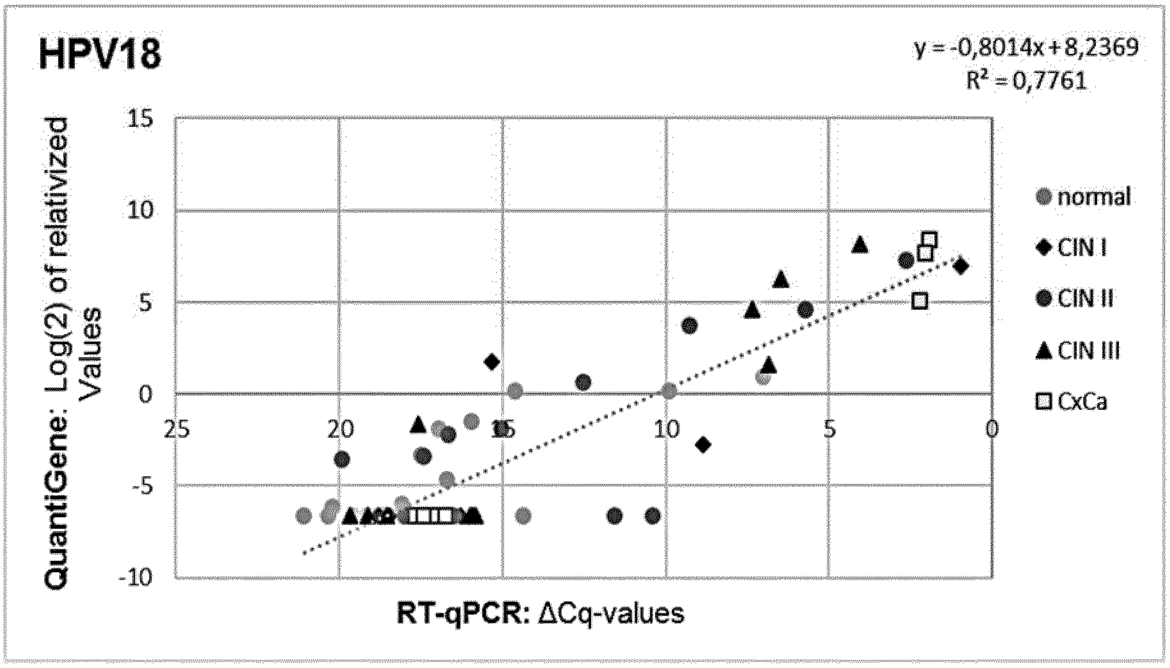

B

marker	correlation coefficient
STMN1	-0,63
MCM2	-0,64
Ki67	-0,65
BIRC5	-0,55
ALDH1A1	-0,66
HPV16-E7	-0,70
HPV18-E7	-0,88

Fig 17

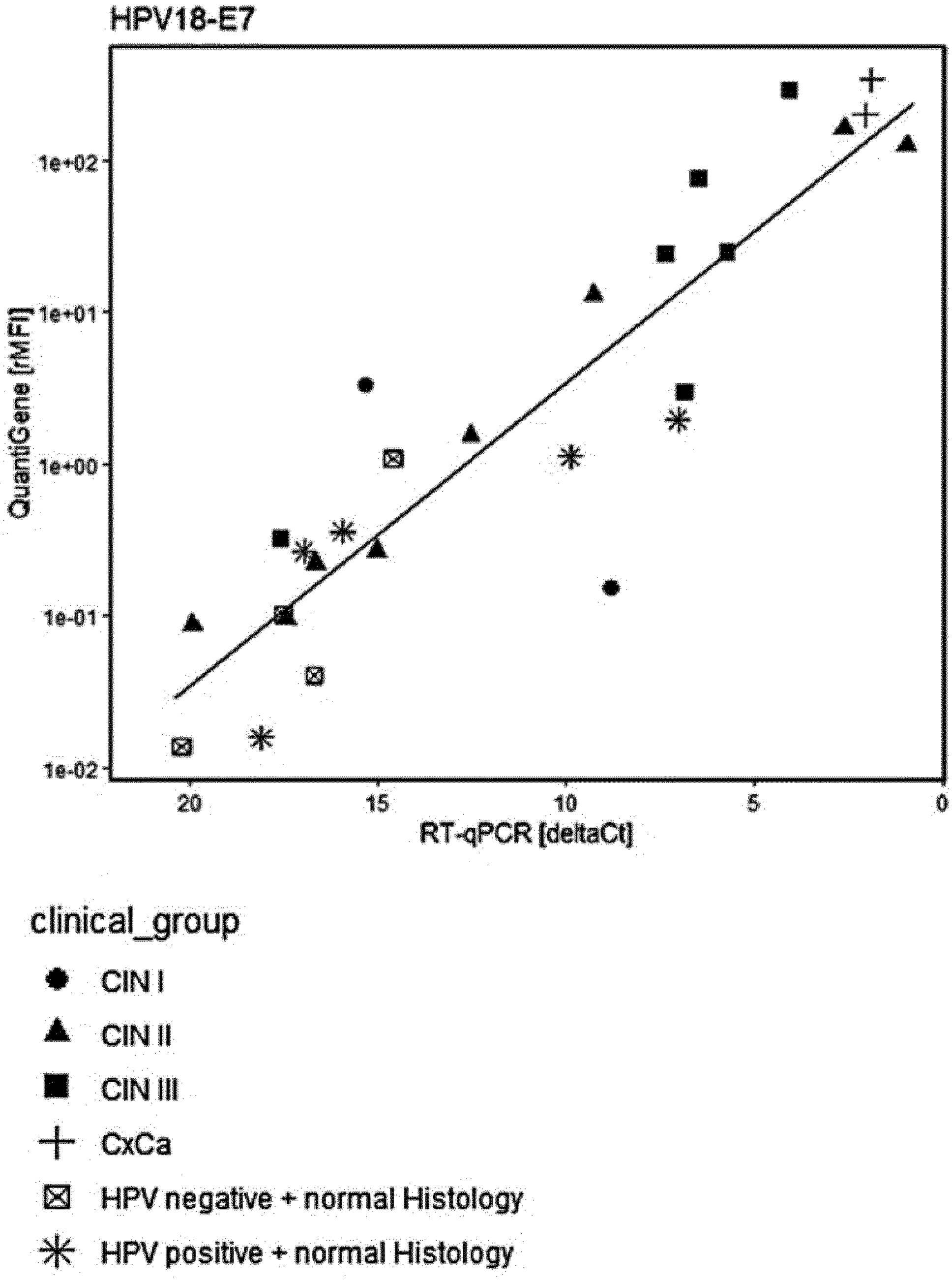
HPV18-E7
1e+02
1e+01
1e+00
1e-01
1e-02
QuantiGene [rMFI]
20
15
10
5
0
RT-qPCR [deltaCt]
clinical_group
CIN I
CIN II
CIN III
CxCa
HPV negative + normal Histology
HPV positive + normal Histology

Fig. 17 (cont.)

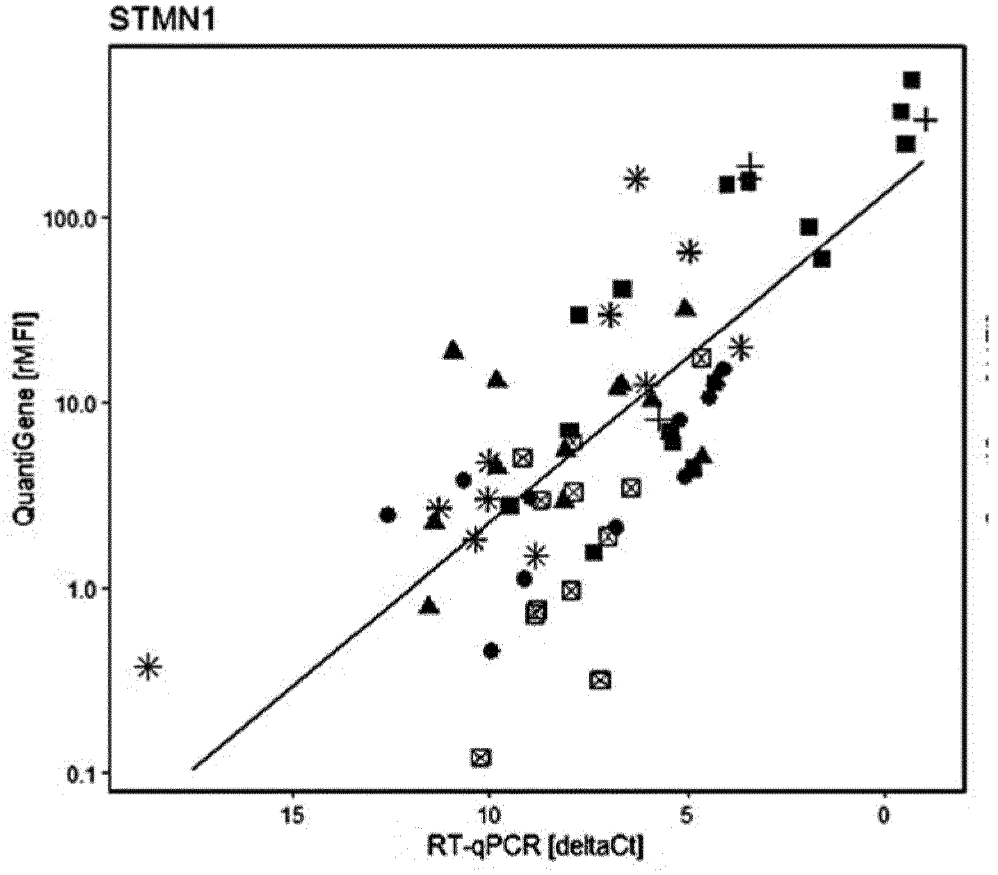
STMN1
QuantiGene [rMFI]
100.0
10.0
1.0
0.1
15
10
5
0
RT-qPCR [deltaCt]

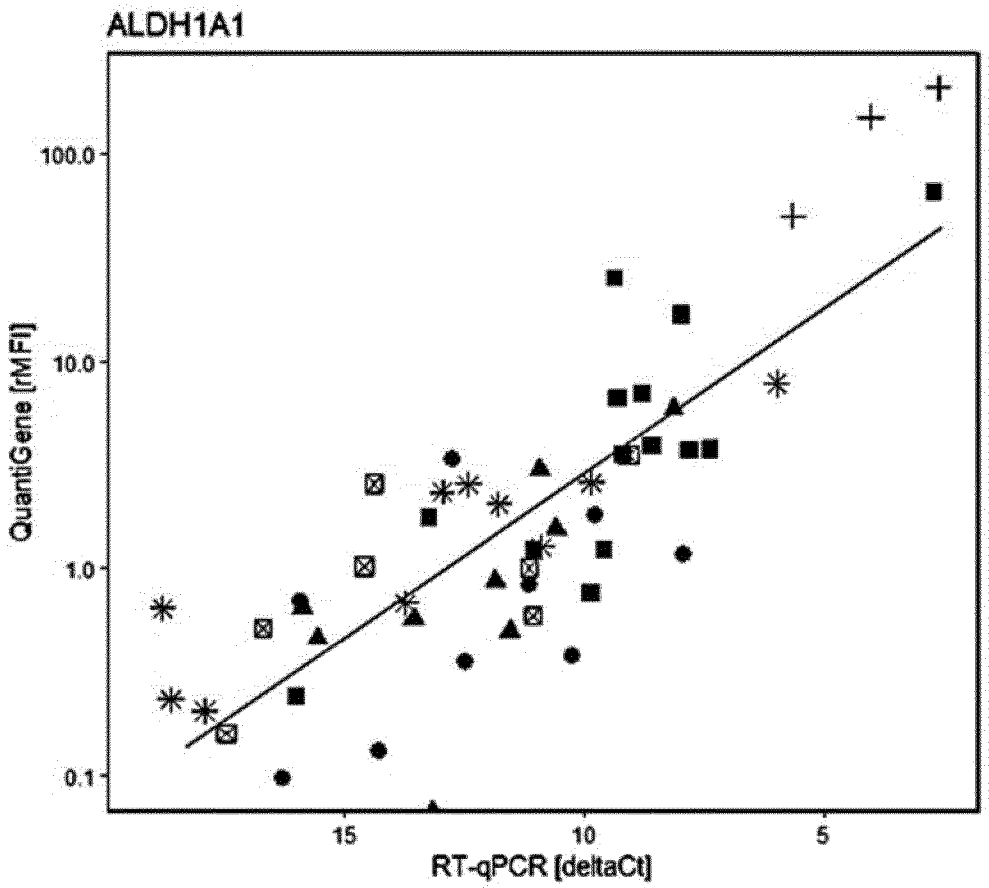
ALDH1A1
QuantiGene [rMFI]
100.0
10.0
1.0
0.1
15
10
5
RT-qPCR [deltaCt]

Fig 17 (cont.)

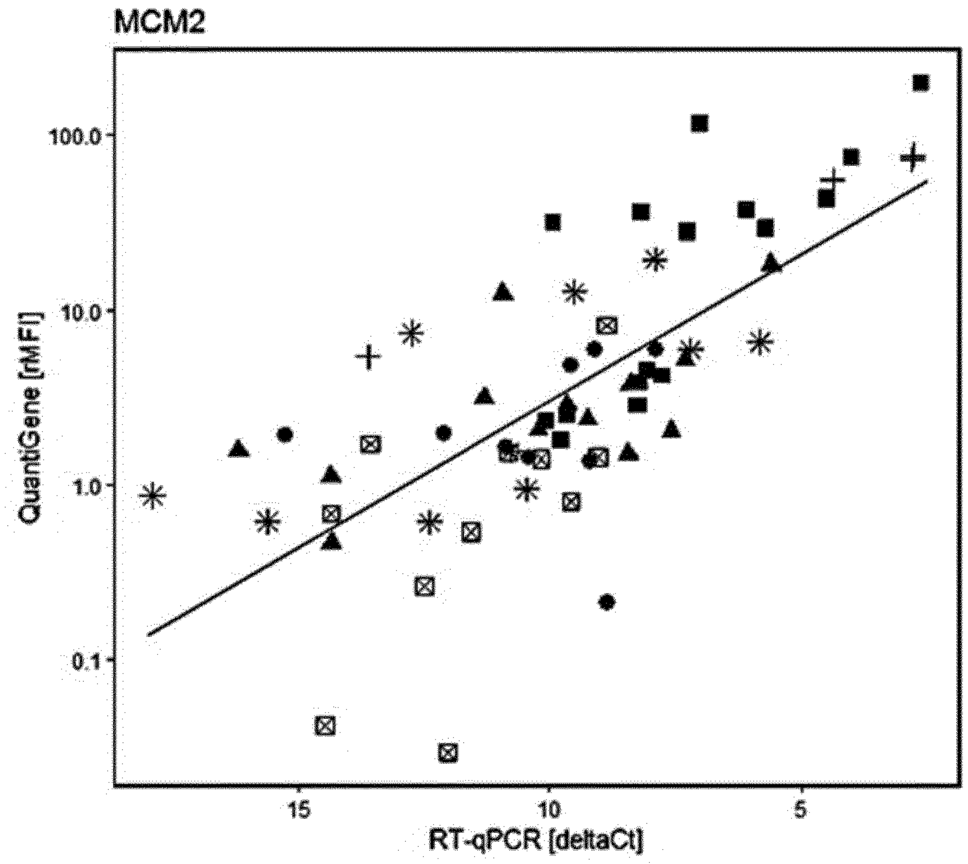
MCM2
100.0
10.0
1.0
0.1
QuantiGene [rMFI]
15
10
5
RT-qPCR [deltaCt]

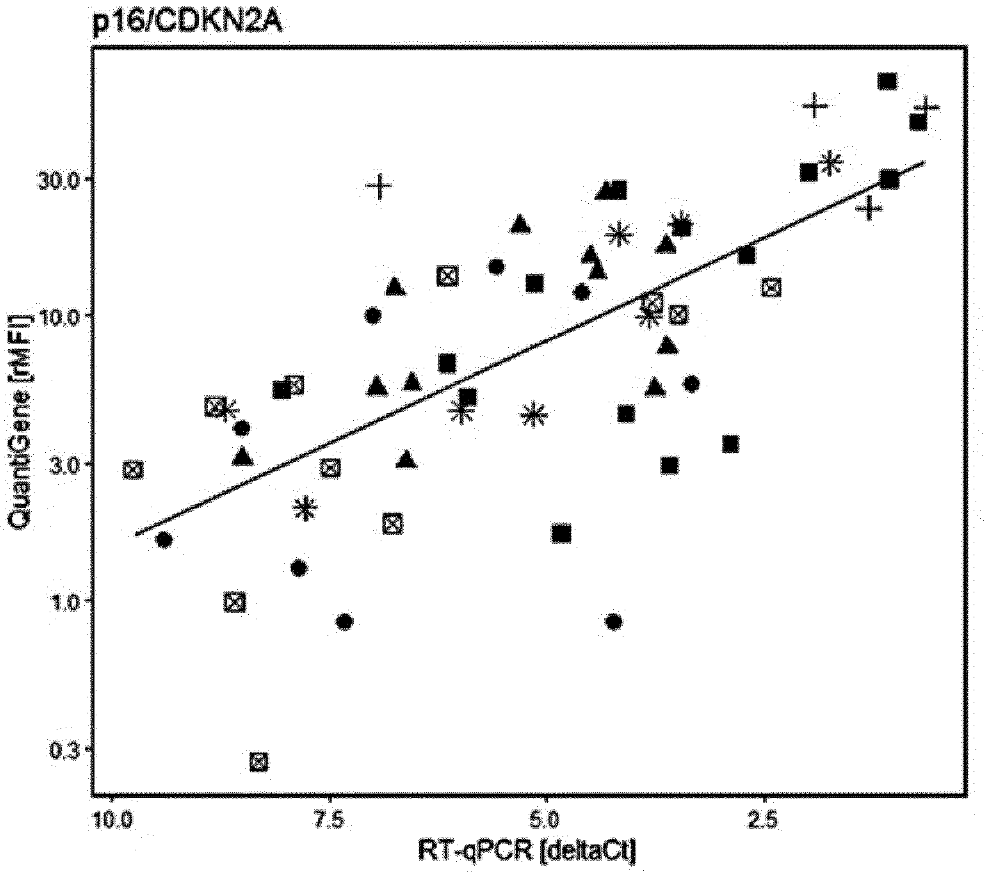
p16/CDKN2A
30.0
10.0
3.0
1.0
0.3
QuantiGene [rMFI]
10.0
7.5
5.0
2.5
RT-qPCR [deltaCt]

Fig 17 (cont.)

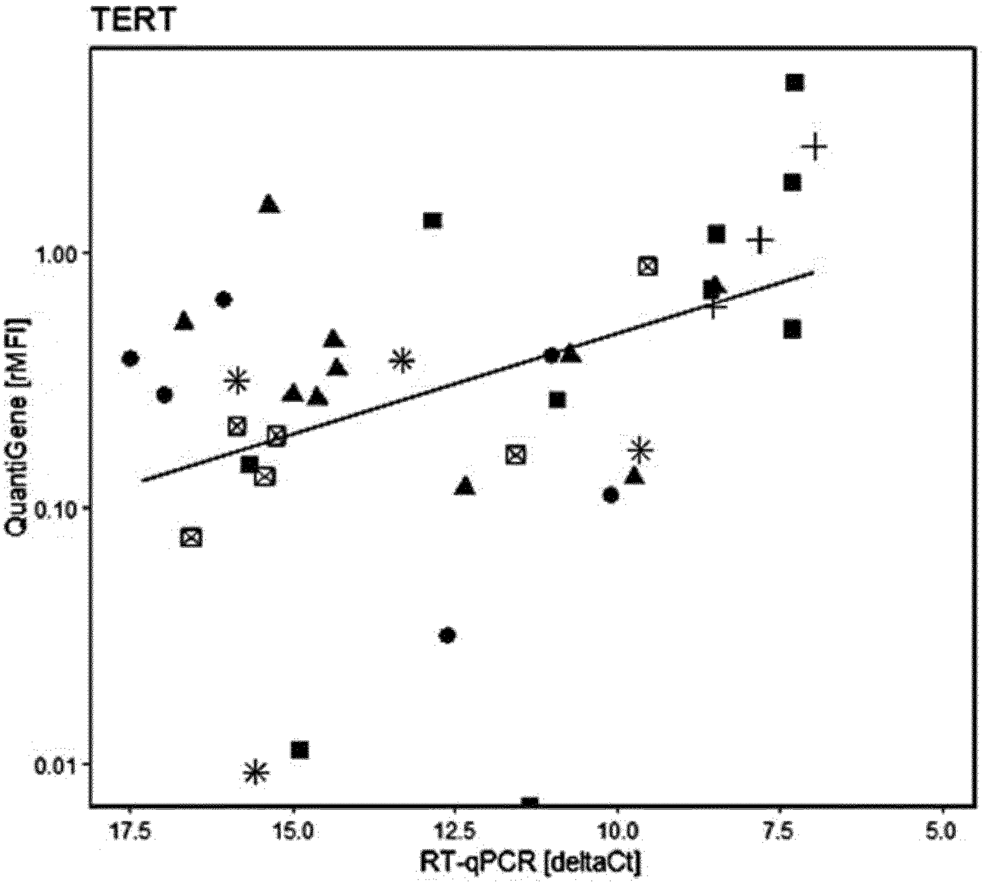
TERT
QuantiGene [rMFI]
1.00
0.10
0.01
17.5
15.0
12.5
10.0
7.5
5.0
RT-qPCR [deltaCt]

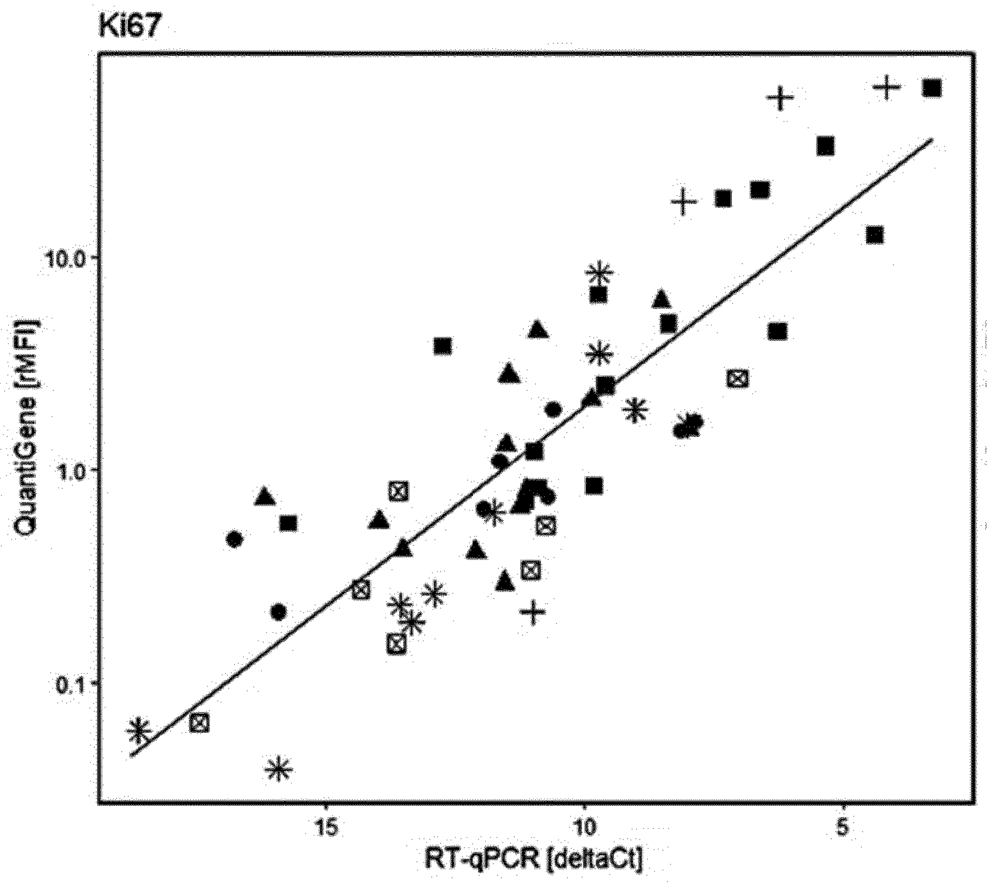
Ki67
QuantiGene [rMFI]
10.0
1.0
0.1
15
10
5
RT-qPCR [deltaCt]

Fig 17 (cont.)

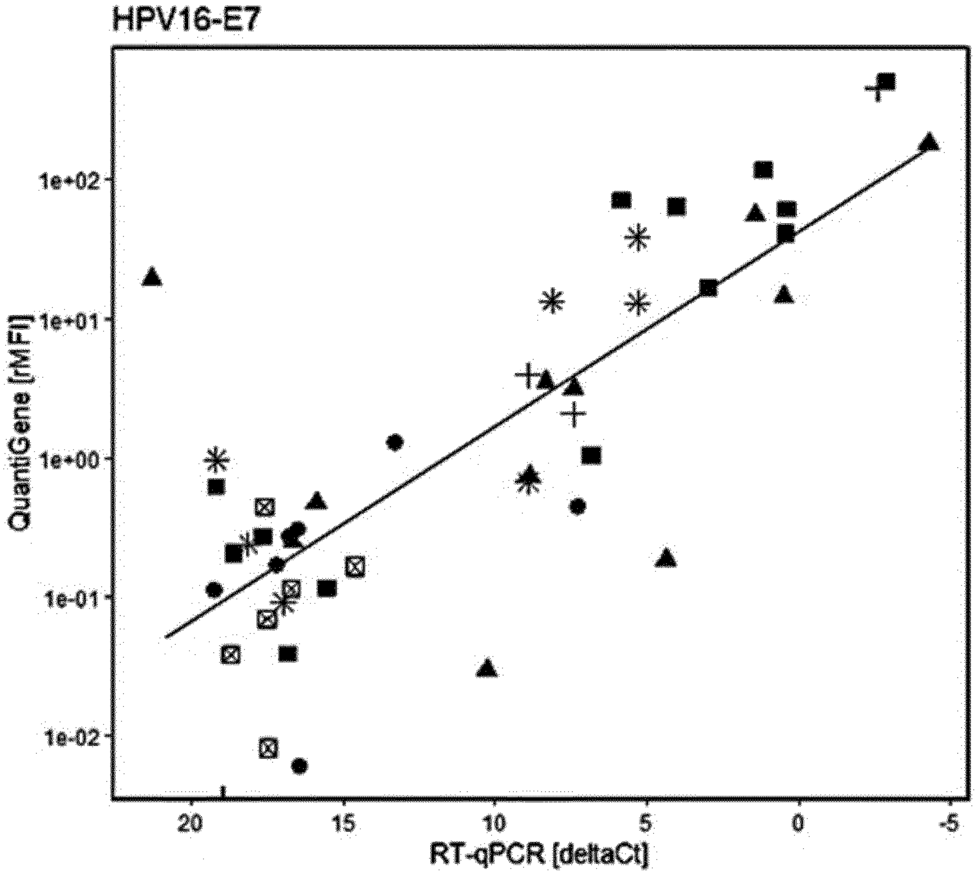
HPV16-E7
QuantiGene [rMFI]
1e+02
1e+01
1e+00
1e-01
1e-02
20
15
10
5
0
-5
RT-qPCR [deltaCt]

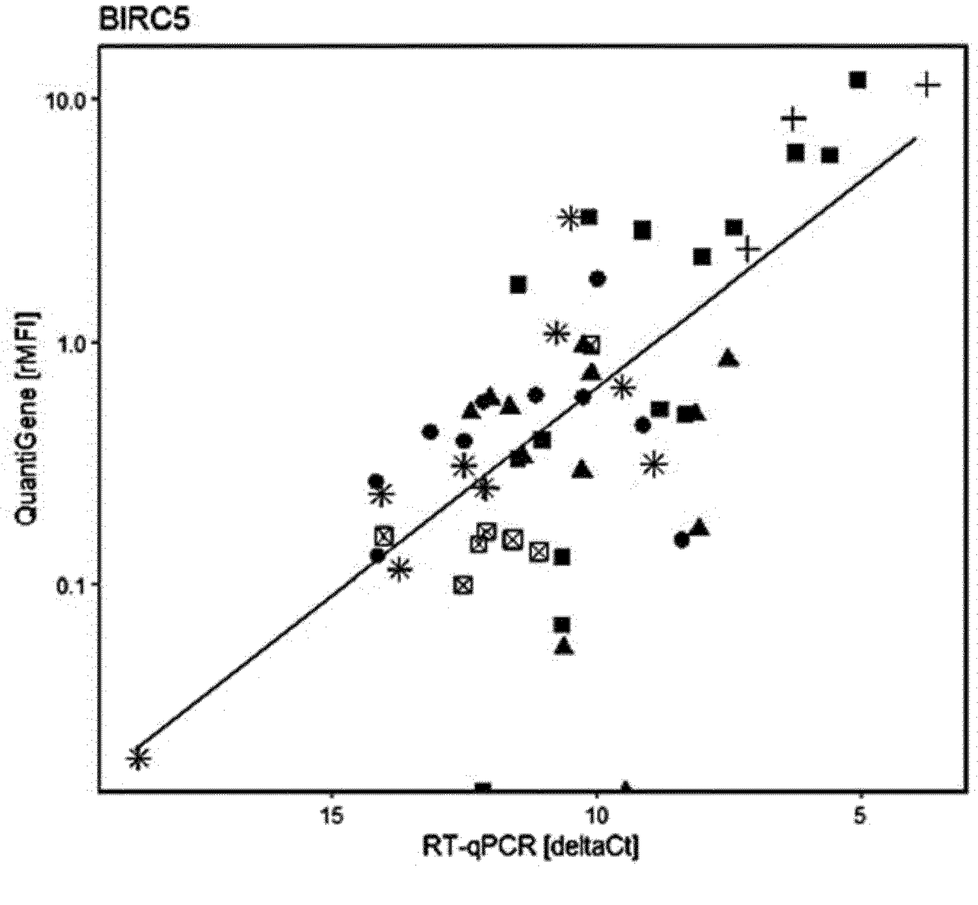
BIRC5
QuantiGene [rMFI]
10.0
1.0
0.1
15
10
5
RT-qPCR [deltaCt]

5

Fig. 18

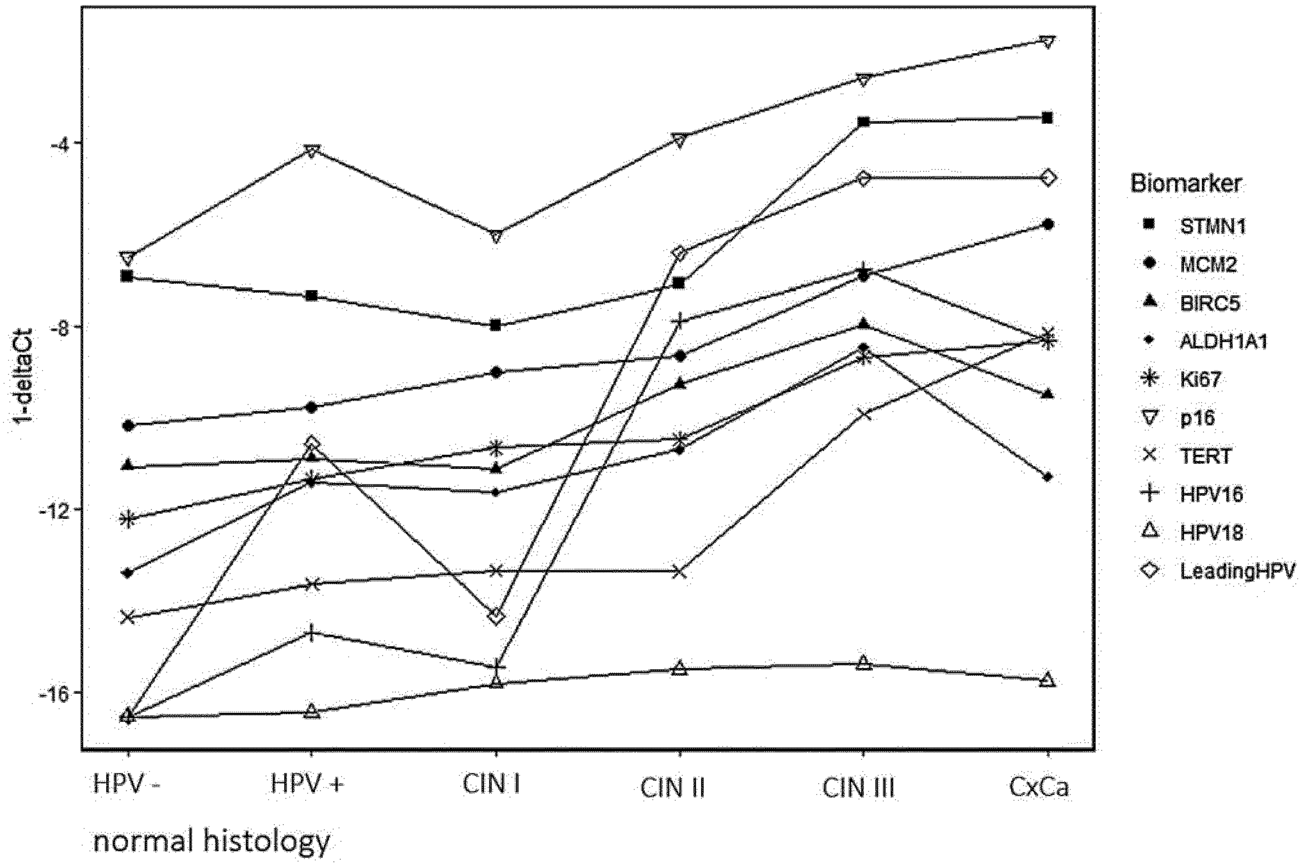
1-deltaCt
-4
-8
-12
-16
HPV -
HPV +
CIN I
CIN II
CIN III
CxCa
normal histology
Biomarker
STMN1
MCM2
BIRC5
ALDH1A1
Ki67
p16
TERT
HPV16
HPV18
LeadingHPV

Fig. 19

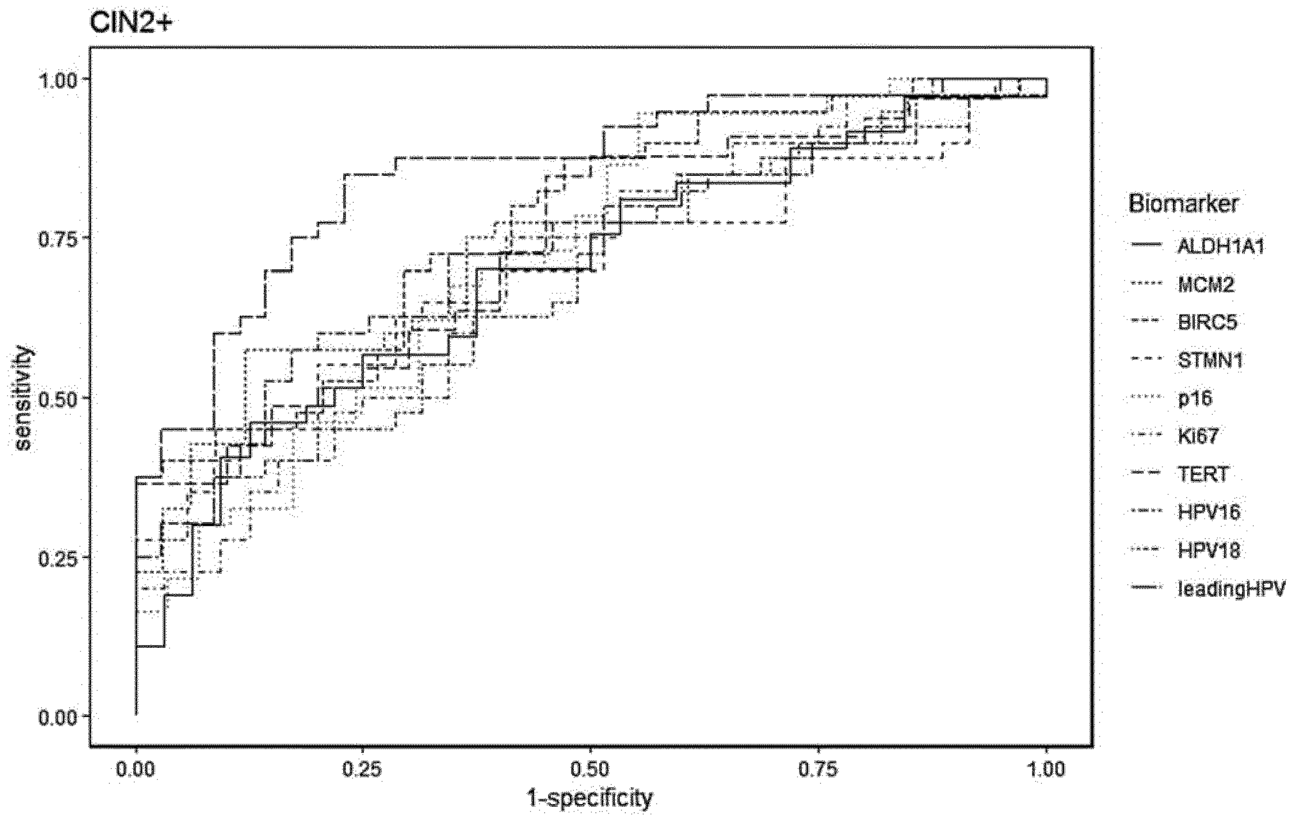
CIN2+
sensitivity
1.00
0.75
0.50
0.25
0.00
0.00
0.25
0.50
0.75
1.00
1-specificity
Biomarker
ALDH1A1
MCM2
BIRC5
STMN1
p16
Ki67
TERT
HPV16
HPV18
leadingHPV

Fig. 20

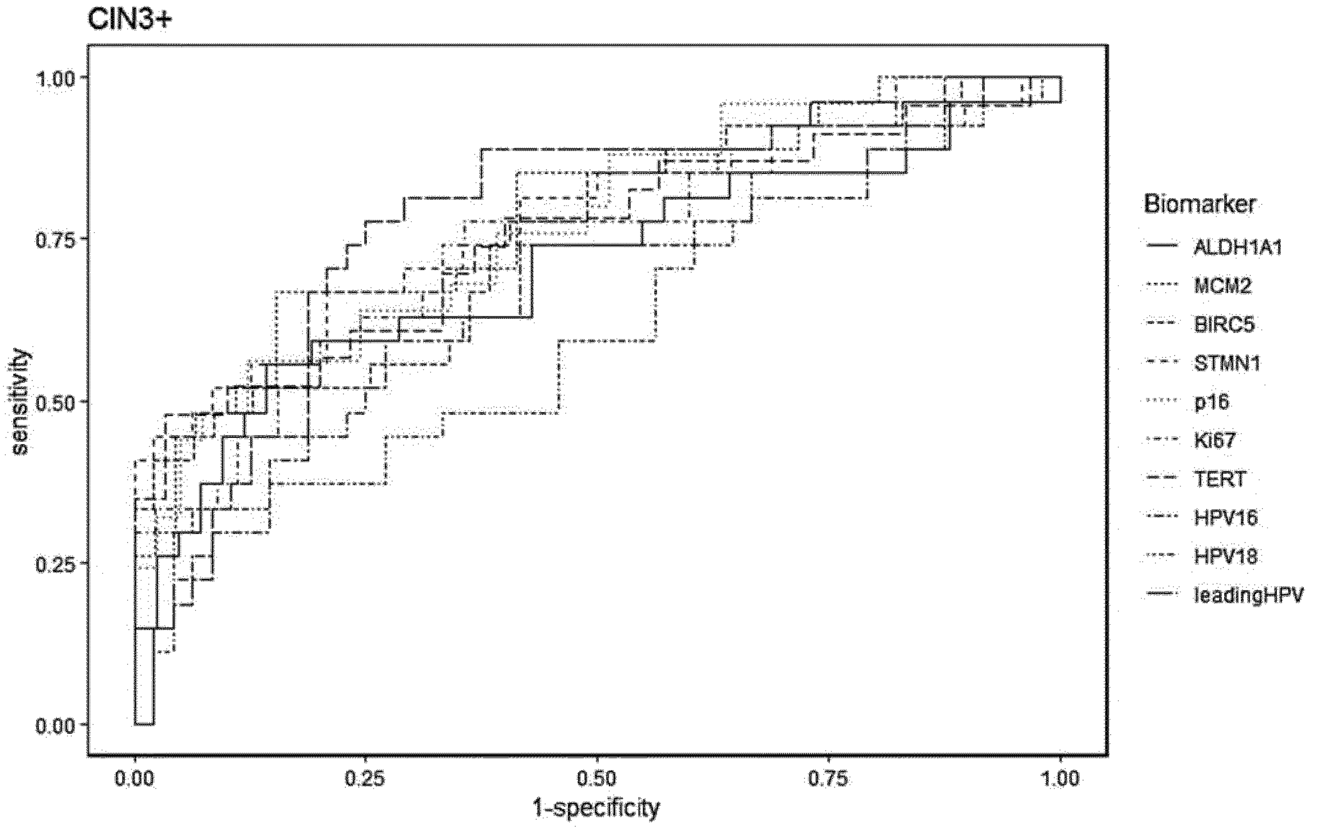
CIN3+
1.00
0.75
0.50
0.25
0.00
sensitivity
0.00
0.25
0.50
0.75
1.00
1-specificity
Biomarker
ALDH1A1
MCM2
BIRC5
STMN1
p16
Ki67
TERT
HPV16
HPV18
leadingHPV

Fig. 21

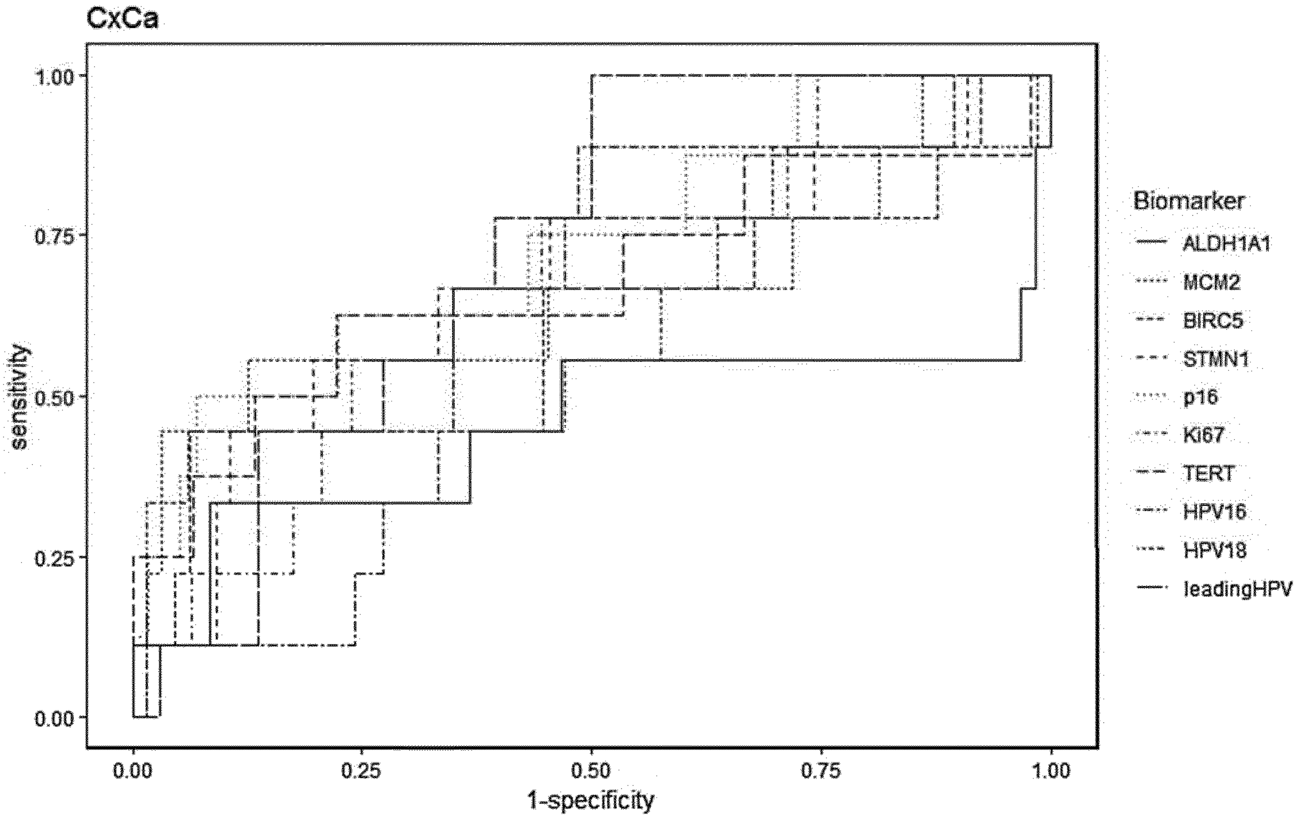
CxCa
1.00
0.75
0.50
0.25
0.00
sensitivity
0.00
0.25
0.50
0.75
1.00
1-specificity
Biomarker
ALDH1A1
MCM2
BIRC5
STMN1
p16
Ki67
TERT
HPV16
HPV18
leadingHPV

INTERNATIONAL SEARCH REPORT

International application No
PCT/EP2020/053095

A. CLASSIFICATION OF SUBJECT MATTER
INV. C12Q1/6886 C12Q1/70
ADD.

According to International Patent Classification (IPC) or to both national classification and IPC

B. FIELDS SEARCHED

Minimum documentation searched (classification system followed by classification symbols)
C12Q

Documentation searched other than minimum documentation to the extent that such documents are included in the fields searched

Electronic data base consulted during the international search (name of data base and, where practicable, search terms used)

EPO-Internal, BIOSIS, EMBASE, WPI Data

C. DOCUMENTS CONSIDERED TO BE RELEVANT

Category*	Citation of document, with indication, where appropriate, of the relevant passages	Relevant to claim No.
X	US 2004/202996 A1 (WILLIAMS INNA R [US] ET AL) 14 October 2004 (2004-10-14) paragraph [0034]; claim 13 -----	1-8, 13-21
A	MARK F. EVANS ET AL: "HPV E6/E7 RNA In Situ Hybridization Signal Patterns as Biomarkers of Three-Tier Cervical Intraepithelial Neoplasia Grade", PLOS ONE, vol. 9, no. 3, 13 March 2014 (2014-03-13), page e91142, XP055311296, DOI: 10.1371/journal.pone.0091142 figures 1-3; table 2 -----	1-21
A	US 2017/204481 A1 (RABBANI ELAZAR [US] ET AL) 20 July 2017 (2017-07-20) examples 8, 22 -----	1-21
	-/--	

[X] Further documents are listed in the continuation of Box C.

[X] See patent family annex.

* Special categories of cited documents :

"A" document defining the general state of the art which is not considered to be of particular relevance

"E" earlier application or patent but published on or after the international filing date

"L" document which may throw doubts on priority claim(s) or which is cited to establish the publication date of another citation or other special reason (as specified)

"O" document referring to an oral disclosure, use, exhibition or other means

"P" document published prior to the international filing date but later than the priority date claimed

"T" later document published after the international filing date or priority date and not in conflict with the application but cited to understand the principle or theory underlying the invention

"X" document of particular relevance; the claimed invention cannot be considered novel or cannot be considered to involve an inventive step when the document is taken alone

"Y" document of particular relevance; the claimed invention cannot be considered to involve an inventive step when the document is combined with one or more other such documents, such combination being obvious to a person skilled in the art

"&" document member of the same patent family

Date of the actual completion of the international search	Date of mailing of the international search report
4 May 2020	14/05/2020

Name and mailing address of the ISA/	Authorized officer
European Patent Office, P.B. 5818 Patentlaan 2<br>NL - 2280 HV Rijswijk<br>Tel. (+31-70) 340-2040,<br>Fax: (+31-70) 340-3016	Santagati, Fabio

1

International application No
PCT/EP2020/053095

C(Continuation). DOCUMENTS CONSIDERED TO BE RELEVANT

Category*	Citation of document, with indication, where appropriate, of the relevant passages	Relevant to claim No.
A	WANG HYE-YOUNG ET AL: "Diagnostic performance of HPV E6/E7, hTERT, and Ki67 mRNA RT-qPCR assays on formalin-fixed paraffin-embedded cervical tissue specimens from women with cervical cancer",<br>EXPERIMENTAL AND MOLECULAR PATHOLOGY, ACADEMIC PRESS, US,<br>vol. 98, no. 3, 30 March 2015 (2015-03-30), pages 510-516, XP029575359,<br>ISSN: 0014-4800, DOI: 10.1016/J.YEXMP.2015.03.036<br>the whole document<br>-----	1-21
A	HAI-RUI WANG ET AL: "A cocktail of p16^{INK4a} and Ki-67, p16^{INK4a} and minichromosome maintenance protein 2 as triage tests for human papillomavirus primary cervical cancer screening",<br>ONCOTARGET,<br>vol. 8, no. 48,<br>13 October 2017 (2017-10-13), pages 83890-83899, XP055691496,<br>DOI: 10.18632/oncotarget.19870<br>tables 4-6<br>-----	1-21
A	EP 1 369 694 A1 (MTM LAB AG [DE])<br>10 December 2003 (2003-12-10)<br>the whole document<br>-----	1-21

1

INTERNATIONAL SEARCH REPORT

Information on patent family members

International application No
PCT/EP2020/053095

Patent document cited in search report	Publication date	Patent family member(s)	Publication date
US 2004202996 A1	14-10-2004	EP 1616177 A2	18-01-2006
		US 2004202996 A1	14-10-2004
		US 2007243552 A1	18-10-2007
		WO 2004092734 A2	28-10-2004
US 2017204481 A1	20-07-2017	NONE	
EP 1369694 A1	10-12-2003	AT 382863 T	15-01-2008
		AT 497169 T	15-02-2011
		AU 2003274105 A1	23-01-2004
		CN 1643378 A	20-07-2005
		DE 60318415 T2	08-01-2009
		EP 1369694 A1	10-12-2003
		EP 1493032 A1	05-01-2005
		EP 1821102 A1	22-08-2007
		ES 2297209 T3	01-05-2008
		ES 2360342 T3	03-06-2011
		JP 4290118 B2	01-07-2009
		JP 4809408 B2	09-11-2011
		JP 2005523039 A	04-08-2005
		JP 2009000127 A	08-01-2009
		MX PA04009584 A	11-01-2005
		US 2005255468 A1	17-11-2005
		US 2009104609 A1	23-04-2009
		US 2012052510 A1	01-03-2012
		US 2014308660 A1	16-10-2014
		US 2017268068 A1	21-09-2017
		US 2019177804 A1	13-06-2019
		WO 2004005927 A1	15-01-2004
